# Cross-national inequalities and public health policy effects of the global retinoblastoma burden from 1990 to 2021 and projections to 2035

**DOI:** 10.3389/fpubh.2026.1753390

**Published:** 2026-01-26

**Authors:** Zutong Huang, Na Zhou, Binwei Yu, Yaqun Wang, Wei Shao, Hanxiang Chen, Min Li, Ji Luo, Ni Yuan

**Affiliations:** 1 School of Public Health, Dalian Medical University, Dalian, Liaoning, China; 2 School of Public Health, Peking University, Beijing, China; 3 Dong Fureng Institute of Economic and Social Development, Wuhan University, Wuhan, Hubei, China; 4 Liaoning Institute of Basic Medical Sciences, Shenyang, Liaoning, China; 5 Tianfu College of SWUFE, Chengdu, Sichuan, China; 6 School of Public Health, Jiamusi University, Jiamusi, Heilongjiang, China; 7 The First Hospital of China Medical University, Shenyang, Liaoning, China

**Keywords:** Global Burden of Disease, health inequality, rare disease policy, retinoblastoma, socio-demographic index

## Abstract

**Objective:**

To examine global retinoblastoma(RB) burden from 1990 to 2021, its cross-national inequalities, the effect of public health policies, and to project disease burden trends to 2035.

**Methods:**

The study examined the global, regional, and national disease burden of RB from 1990 to 2021, applying data from the Global Burden of Disease (GBD) to assess incidence, prevalence, disability-adjusted life years (DALYs), and death. Estimated annual percentage change (EAPC), connection point regression, and autoregressive moving average (ARIMA) models were used to evaluate the overall, segmented, and projection trend of the disease burden. In addition, we also examined the inequity of disease burden at various levels of the socio-demographic index (SDI) and compared the policy effects for rare diseases in countries with differing levels of socio-economic development.

**Results:**

From 1990 to 2021, the global incidence and prevalence of RB continued to rise, while the death rate and DALYs generally trended downward; however, significant differences were observed between regions and countries. By 2035, the number of incidence and prevalence is expected to decline slightly year by year, while the number of DALYs and deaths is expected to continue to rise. Countries with low Socio-demographic Index (SDI) bear a disproportionately higher disease burden. Moreover, between 1990 and 2021, absolute inequality decreased, while relative inequality increased. In addition, the impact analysis on the policy of rare diseases in countries at different levels of development shows that there is a clear correlation between a more comprehensive policy framework and the reduction of the burden of RB, among which the improvement in Central Europe is the most significant.

**Conclusion:**

The study reveals that the global burden of RB is generally in a downward trend; however, low SDI regions continue to bear a disproportionately higher burden, underscoring persistent health inequalities. Policy improvement plays a key role in reducing the burden of disease. To further narrow regional disparities and reduce the overall burden, policy building needs to be strengthened globally, particularly in underdeveloped areas. The findings of this study may also provide valuable references for the prevention and treatment of other rare diseases and the promotion of health equity.

## 1 Introduction

Retinoblastoma (RB) is the most common malignant tumor in children, accounting for 2% of all pediatric cancers (1), although it is still considered a rare disease (2). The disease mainly occurs in children under the age of five, with approximately 90% of cases diagnosed before the age of three (3). Its incidence is about 1 case per 15,000 to 18,000 babies born alive, and no significant differences have been observed between different races or genders (4). RB is an invasive eye cancer that can lead to death within 1 to 2 years if not treated (5). In addition, delayed or inadequate treatment may lead to vision loss, which seriously affects the patient’s quality of life (6).

In May 2025, the World Health Assembly adopted a resolution on rare diseases, emphasizing the burden faced by patients with rare diseases, especially children, and the importance of addressing the issue of health equity (7, 8). However, due to the uneven distribution of medical resources (9, 10), there is a significant variation in the global burden of RB. In high-income countries, there are advanced treatment options, including techniques like intravitreal or intra-arterial chemo (11), and the overall 5-year survival rate can reach 94–95% (12). A small number of early cases can preserve eyeballs and vision through local treatment (13). Conversely, in low and middle income countries, insufficient screening and delayed diagnosis frequently result in patients receiving treatment at advanced stages, leading to a poor prognosis and an approximate survival rate of 50% (14).

Following established disease burden research frameworks (15, 16), this study uses data from the Global Disease Burden (GBD) database to systematically analyze the incidence, prevalence, disability-adjusted life years (DALYs), and death of RB from 1990 to 2021. Building on previous studies, we further examine cross-national inequalities in RB burden and explore the effect of public health policies and project disease burden trends to 2035.

## 2 Methods

### 2.1 Data sources

This study was based on the GBD database, from which epidemiological data on RB between 1990 and 2021 were collected, including incidence, prevalence, DALYs, and death as the core indicators. The GBD database provides age-standardized rates (ASRs) at the global, regional, and national levels. It categorizes countries into five Socio-demographic Index (SDI) strata (high, high–middle, middle, low–middle, and low) as well as 21 GBD geographic regions. Given the diversity of data sources and uncertainties due to missing data and modeling assumptions, the GBD database employs Bayesian modeling for data integration and reports 95% uncertainty intervals (95% UI) to reflect the reliability and variability of the estimates (17).

The policy documents included in the impact analysis were retrieved from the official websites of health-related governmental authorities in the target countries. Inclusion criteria: (1) Policy documents formally issued by national-level authoritative institutions; (2) Documents that explicitly address the field of rare diseases, including policies, plans, guidelines, notices, or administrative regulations; (3) Documents released up to September 2025. Exclusion criteria: (1) Policy documents that do not specifically target the field of rare diseases; (2) Documents issued by subnational governments, industry associations, academic societies, or other non-national institutions; (3) Duplicated documents; (4) Non-official documents. Supplementary Table S12 shows the specific policy materials.

### 2.2 Statistical analysis

#### 2.2.1 Descriptive analysis

Descriptive analyzes were conducted at global, regional, and national levels. The numbers and ASRs of incidence, prevalence, DALYs, and death for RB were examined worldwide from 1990 to 2021. Furthermore, changes in the numbers and ASRs of incidence, prevalence, DALYs, and death were compared between 1990 and 2021 across the global, 21 GBD regions, 204 countries and regions, and five SDI strata.

Among these indicators, DALYs are used as a comprehensive indicator to measure the overall health loss of the population. DALY is equivalent to the loss of 1 year of completely healthy life. It comprehensively considers the number of years of life lost as a result of impairment and the number of years lost due to early death (18).

#### 2.2.2 Trend analysis

##### 2.2.2.1 Overall trends

The estimated annual percentage change (EAPC) was used to evaluate temporal trends in RB burden from 1990 to 2021. A linear regression model was fitted as y = *α* + *β*x, where y = ln(ASR) and x = calendar year. EAPC was calculated as (exp(β) − 1) × 100%. The 95% confidence interval (95% CI) of EAPC was derived from the model to reflect uncertainty in trend estimates. An increasing trend was defined as one in which the EAPC estimate and its 95% CI lower bound were both greater than 0. Conversely, a decreasing trend was determined if the EAPC estimate and its 95% CI upper bound were both <0. Otherwise, the ASR was considered stable.

##### 2.2.2.2 Segmented trends

To further identify segmented variations in RB burden during the study period, joinpoint regression analysis was applied. The method identifies the inflection points in the time trend, divides the data from 1990 to 2021 into multiple intervals, and estimates the annual percentage change (APC) of each interval. The average annual percentage change (AAPC) is calculated as the weighted average (19) of the regression slope of each interval. Monte Carlo permutation tests were conducted with 4,499 randomly permuted datasets to estimate the 95% CI for APC and AAPC, with Bonferroni correction applied to control the overall significance level (20). The trend assessment standard is consistent with EAPC.

##### 2.2.2.3 Projection trends

We utilized the autoregressive moving average (ARIMA) model to project the future burden of RB from 2022 to 2035, employing historical data from 1990 to 2021 to fit the incidence, prevalence, DALYs, and death rates. This model can effectively explain long-term trends and stochastic fluctuations in time series, and has been widely used in epidemiology and public health projection (21). The results are reported in the form of a 95% reliable interval (95% CrI), which is based on Bayesian inference, indicating the probability that the real parameter value falls within the interval (22) under the given data and model assumptions.

#### 2.2.3 Health inequality analysis

##### 2.2.3.1 Cross-national inequality analysis based on SII and CI

Monitoring health inequality provides an evidence-based basis for health planning (23). We utilized two standard indicators to quantify the transnational inequality of the RB disease burden: the inequality slope index (SII) and the concentration index (CI) to measure absolute and relative disparities. The calculation method of SII involves regression analysis of the national DALY rate across all age groups and the relative position of countries on the SDI (24). SII > 0 indicates that as national development levels improve, the DALY rate also increases (the disease burden is concentrated in countries with high SDI), and vice versa. The calculation method of the concentration index is to numerically integrate the area under the Lorentz concentration curve (25), which is obtained by fitting the cumulative proportion of the DALY rate and the relative distribution of population accumulation sorted by SDI. CI > 0 indicates that the disease burden is concentrated in countries with low SDI. From 1990 to 2021, the increase in SII and the decrease in CI corresponded to the increase in absolute and relative health inequality.

##### 2.2.3.2 Policy effects analysis on the disease burden

**Figure 1:**
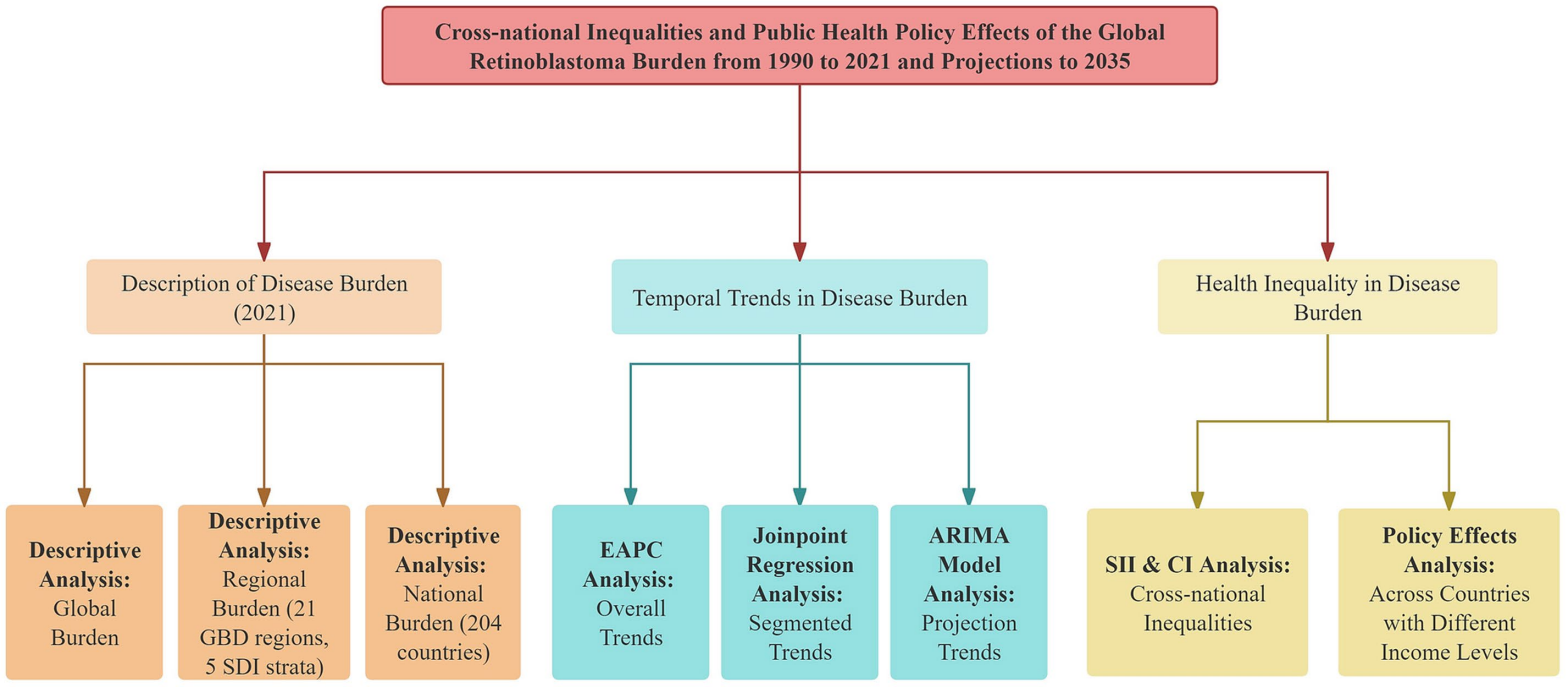
Research framework.

The study further examined the impact of policy effects on the disease burden. We refer to the World Bank’s 2022 classification (26) which categorizes countries into three groups: high-income countries, middle-income countries (encompassing both middle- and low-income countries), and low-income countries. Under this framework, six representative countries were selected: high-income countries (the United States, the United Kingdom), middle-income countries (China, Brazil), and low-income countries (Niger, Ethiopia). The selection of these countries is mainly based on two considerations: (1) covering countries with three levels of development to highlight the policy differences between countries at different levels of development; (2) covering multiple regions (Europe, Asia, Latin America, Africa) to reduce regional bias. Under the guidance of the WHO framework for the whole process of rare diseases, we systematically reviewed the relevant rare disease policies of these countries across six domains (management, prevention, diagnosis and treatment, research and development and registration, medical insurance, and other rare disease-related policies). The study compared the number and timing of policies introduced in various countries and combined the analysis of overall and segmented trends to explore the relationship between policy improvement and the reduction of RB disease burden (Figure 1).

## 3 Results

### 3.1 Global, regional, and national burden of RB

At the global level, in 2021 the numbers of incidence prevalence, DALYs, and deaths from RB were 6,275 (95% UI: 3,855–8,382), 57,333 (95% UI: 35,247–76,615), 243,204 (95% UI: 147,358–330,474), and 2,762 (95% UI: 1,666–3,761), respectively. The corresponding age-standardized incidence rate (ASIR), prevalence rate (ASPR), DALY rate (ASR-DALY), and death rate (ASDR) were 0.09 (95% UI: 0.06–0.13), 0.86 (95% UI: 0.53–1.15), 3.65 (95% UI: 2.21–4.96), and 0.04 (95% UI: 0.03–0.06), respectively (Table 1; Supplementary Tables S1–S3).

**Table 1:** The number of DALYs and ASR-DALY of RB by region in 1990 and 2021, along with its EAPC.

Location	1990		2021		EAPC (95%CI)1990–2021
	Number (95% UIs)	ASR (95% UIs)	Number (95% UIs)	ASR (95% UIs)	
Global	279066 (159656, 365150)	4.54 (2.60, 5.94)	243204 (330474, 147358)	3.65 (2.21, 4.96)	−0.42 (−0.53, −0.31)
SDI strata					
High SDI	3920 (3251, 4619)	0.63 (0.52, 0.74)	1445 (1,134, 1778)	0.26 (0.2, 0.32)	−2.05 (−2.51, −1.59)
High-middle SDI	18894 (10621, 28,899)	2.03 (1.14, 3.1)	5661 (2820, 8335)	0.78 (0.39, 1.16)	−2.49 (−2.79, −2.18)
Middle SDI	57028 (33775, 75468)	2.85 (1.69, 3.77)	27522 (15646, 37126)	1.51 (0.86, 2.04)	−1.7 (−1.88, −1.51)
Low-middle SDI	99163 (53558, 138758)	5.79 (3.13, 8.09)	80918 (48497, 111997)	4.2 (2.52, 5.82)	−0.68 (−0.79, −0.57)
Low SDI	99940 (55473, 137643)	11.38 (6.32, 15.64)	127543 (76895, 183903)	7.8 (4.7, 11.24)	−0.95 (−1.09, −0.81)
GBD regions					
Andean Latin America	4199 (2479, 7183)	8.09 (4.77, 13.88)	2310 (1427, 3761)	3.73 (2.31, 6.08)	−2 (−2.17, −1.82)
Australasia	24 (18, 30)	0.15 (0.12, 0.2)	4(2, 7)	0.02 (0.01, 0.04)	−3.64 (−5.37, −1.89)
Caribbean	486 (307, 694)	1.2 (0.76, 1.71)	160 (77, 290)	0.41 (0.2, 0.75)	−2.29 (−3, −1.58)
Central Asia	1395 (643, 2629)	1.52 (0.7, 2.86)	1263 (688, 2247)	1.28 (0.7, 2.27)	−0.16 (−0.38, 0.05)
Central Europe	757 (375, 1503)	0.8 (0.4, 1.6)	117 (76, 181)	0.2 (0.13, 0.32)	−4.91 (−5.64, −4.17)
Central Latin America	8092 (6915, 9539)	3.58 (3.06, 4.23)	3342 (2276, 4746)	1.62 (1.1, 2.3)	−2.08 (−2.47, −1.69)
Central Sub-Saharan Africa	4455 (2018, 7601)	4.53 (2.06, 7.75)	5731(2249, 10991)	2.74 (1.08, 5.26)	−1.15 (−1.4, −0.9)
East Asia	28194 (14624, 43375)	2.45 (1.27, 3.76)	7061 (2980, 10647)	0.88 (0.37, 1.32)	−2.17 (−2.66, −1.69)
Eastern Europe	2162 (1585, 2910)	1.23 (0.9, 1.66)	440 (326, 588)	0.4 (0.3, 0.54)	−4.47 (−5.2, −3.73)
Eastern Sub-Saharan Africa	82748 (48818, 116452)	23.71 (13.9, 33.35)	92696 (61255, 142472)	14.67 (9.7, 22.54)	−1.02 (−1.25, −0.8)
High-income Asia Pacific	801 (589, 1098)	0.74 (0.55, 1.01)	199 (145, 272)	0.29 (0.21, 0.4)	−2.14 (−2.87, −1.4)
High-income North America	1462 (1293, 1661)	0.68 (0.6, 0.77)	501 (374, 686)	0.24 (0.18, 0.32)	−2.6 (−3.19, −2.02)
North Africa and Middle East	6809 (4143, 10721)	1.35 (0.82, 2.12)	3906 (2403, 6212)	0.63 (0.39, 1)	−2.23 (−2.56, −1.9)
Oceania	116 (36, 326)	1.19 (0.37, 3.34)	250 (69, 824)	1.33 (0.37, 4.39)	0.42 (0.04, 0.79)
South Asia	81757 (38571, 122163)	5.23 (2.47, 7.82)	59193 (33196, 87908)	3.68 (2.06, 5.48)	−1.09 (−1.27, −0.9)
Southeast Asia	13508 (5866, 22493)	2.32 (1.01, 3.86)	8421 (3735, 12789)	1.47 (0.65, 2.24)	−1.36 (−1.45, −1.27)
Southern Latin America	686 (453, 1085)	1.33 (0.88, 2.11)	148 (94, 217)	0.32 (0.21, 0.47)	−4 (−4.67, −3.33)
Southern Sub-Saharan Africa	1738 (916, 2636)	2.36 (1.24, 3.57)	2672 (1096, 4853)	3.31 (1.35, 6)	1.95 (1.53, 2.37)
Tropical Latin America	5534 (4315, 7017)	3.14 (2.45, 3.99)	1727 (1156, 2375)	1 (0.67, 1.38)	−2.99 (−3.43, −2.54)
Western Europe	1155 (1017, 1328)	0.5 (0.44, 0.57)	638 (483, 826)	0.29 (0.22, 0.38)	−1.55 (−2.42, −0.67)
Western Sub-Saharan Africa	32988 (15729, 47738)	9.74 (4.64, 14.09)	52424 (22045, 82867)	6.69 (2.81, 10.58)	−1.04 (−1.19, −0.88)

Due to space limitations, the numbers and ASIR, ASPR, ASDR of RB by region in 1990 and 2021, along with their EAPCs, are presented in Supplementary Tables S1–S3.

**Figure 2:**
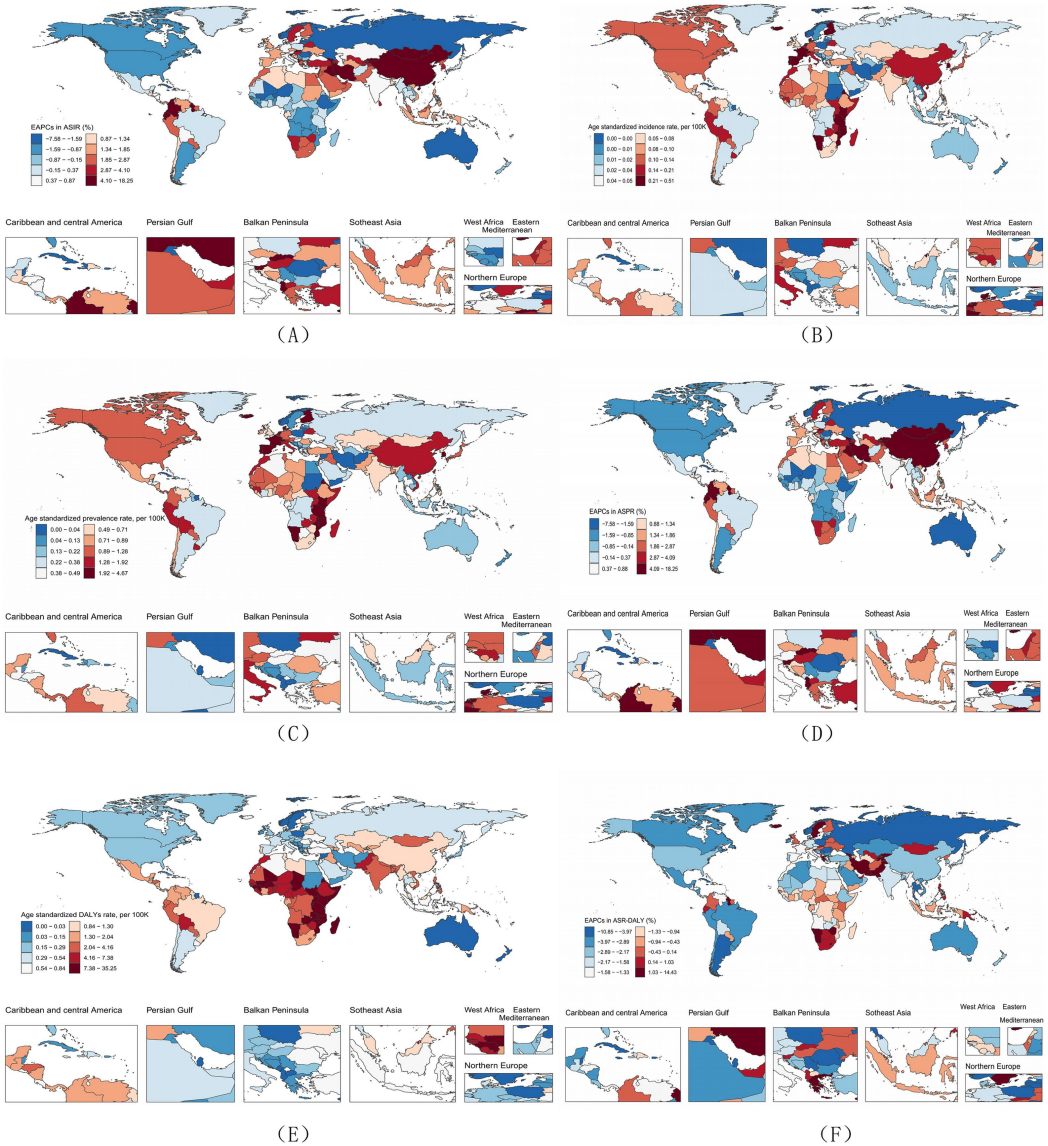
**(A)** ASIR of RB in 2021; **(B)** EAPC in ASIR of RB from 1990 to 2021; **(C)** ASPR of RB in 2021; **(D)** EAPC in ASPR of RB from 1990 to 2021; **(E)** ASR-DALY of RB in 2021; **(F)** TEAPC in ASR-DALY of RB from 1990 to 2021.

By region, in 2021, Oceania had the lowest numbers and ASRs for all four indicators, whereas Eastern Sub-Saharan Africa had the highest (Figure 2; Table 1; Supplementary Tables S1–S3). At the national level, India recorded the highest numbers of incidence and deaths, while China had the highest numbers of prevalence and DALYs. In terms of ASRs, Qatar had the lowest ASIR but ranked third in ASDR; the Syrian Arab Republic had the lowest ASDR; Saint Kitts and Nevis had the lowest ASPR and ASR-DALY; Tokelau had the highest ASIR, ASR-DALY, and ASPR; and the Republic of Malawi recorded the highest ASDR (Figure 2; Supplementary Tables S4–S7).

Across SDI strata, in 2021, the numbers of all four indicators decreased with increasing SDI, with the high SDI group showing the lowest numbers of incidence, prevalence, DALYs, and deaths. For ASRs, the high-middle SDI group had the highest ASIR and ASPR, while the low SDI group had the highest ASDR and ASR-DALY (Table 1; Supplementary Tables S1–S3).

### 3.2 Trends in the global burden of RB

#### 3.2.1 Overall trends of the global, regional, and national levels

Globally, from 1990 to 2021, ASIR and ASPR of RB increased at average annual percent changes of 1.35% (95% UI: 1.13–1.56) and 1.36% (95% UI: 1.15–1.57), respectively. In contrast, ASDR and ASR-DALY declined by 0.42% (95% UI: 0.31–0.53) and 0.44% (95% UI: 0.33–0.55) per year, respectively (Table 1; Supplementary Tables S1–S3).

At the regional level, the increase in ASIR and ASPR in East Asia was the most significant, while the decline in the Caribbean was the largest. ASR-DALY declined in all regions except sub-Saharan Africa. ASDR has also declined in all regions. Among them, the decline in the European region is the most significant (Figure 2; Table 1; Supplementary Tables S1–S3).

At the national level, the trends of 204 countries and regions vary significantly. ASIR and ASDR in Bahrain increased the most, while ASR-DALY and ASDR in Kuwait decreased the most. Overall, ASR-DALY in 180 countries (88.23%) showed a downward trend, and ASDR in 181 countries (88.73%) showed a downward trend (Figure 2; Supplementary Tables S4–S7).

Among all SDI strata, except for the low SDI strata, the number and ASRs of incidence and prevalence increased in the other four strata. However, the number and ASRs of DALY and death declined in all strata, with the most pronounced decreases observed in the high SDI and high-middle SDI groups, and the most minor decreases in the low-middle and low-SDI groups (Table 1; Supplementary Tables S1–S3).

#### 3.2.2 Segmented trends of global burden

**Figure 3:**
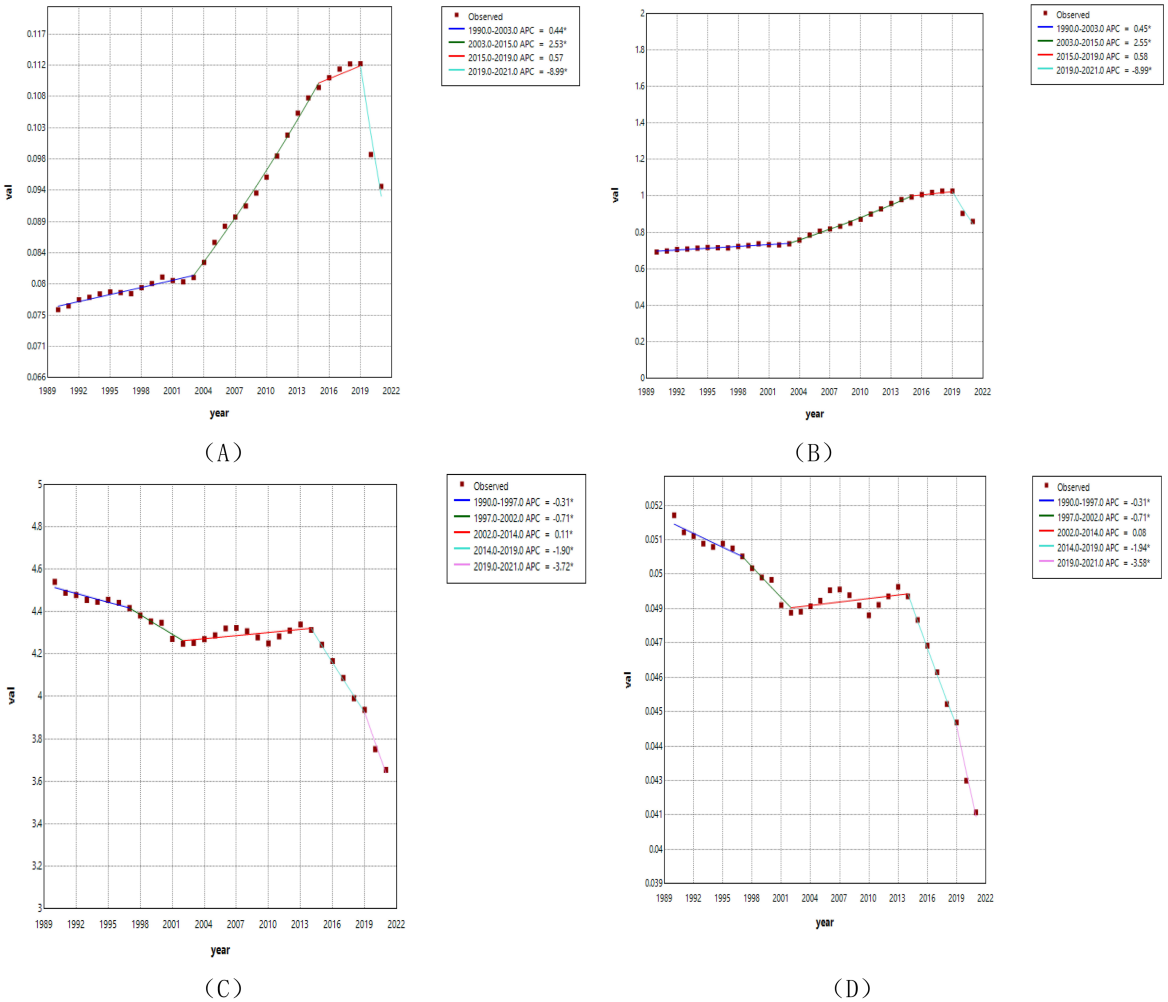
Joinpoint regression analysis for global ASIR **(A)**, ASPR **(B)**, ASR-DALY **(C)**, ASDR **(D)** of RB.

Figure 3 shows the joinpoint regression results for ASRs of RB. Overall, ASIR and ASPR showed increasing trends, whereas ASR-DALY and ASDR showed decreasing trends. However, partial trends at different joinpoints were heterogeneous. Specifically, ASIR and ASPR have experienced four joinpoints: both increased at varying rates during 1990–2003, 2003–2015, and 2015–2019, followed by a decline during 2019–2021 (Supplementary Table S5). In contrast, ASR-DALY and ASDR exhibited five joinpoints: a decreasing trend from 1990 to 1997, a continued decline at varying rates from 1997 to 2002, a slight increase from 2002 to 2014, a decrease from 2014 to 2019, and an accelerated decline from 2019 to 2021 (Supplementary Table S5).

#### 3.2.3 Projection trends of global burden

**Figure 4:**
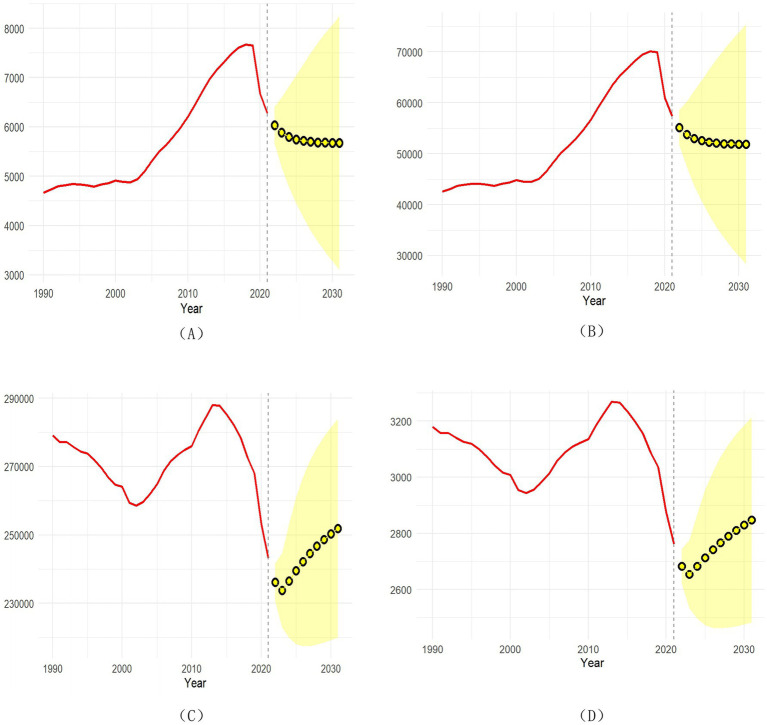
Projection the numbers of incidence **(A)**, prevalence **(B)**, DALYs **(C)**, death **(D)** by 2035. 3.3 Global inequalities in the burden of RB.

Figure 4 shows the projected global numbers of incidence, prevalence, DALYs, and death of RB through 2035. The results suggest that incidence and prevalence cases will gradually decline, while the numbers of DALYs and deaths will continue to rise annually. Detailed projections by year are provided in Supplementary Table S6.

#### 3.2.4 Cross-national inequalities based on SII and CI

**Figure 5:**
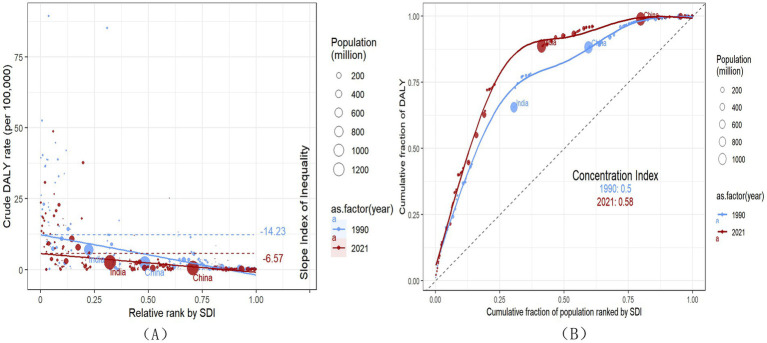
Regression curves **(A)** and concentration curves **(B)** for DALYs of global RB in 1990 and 2021, related to SDI.

Figure 5 shows the absolute and relative inequalities of the SDI related to the burden of RB. In 1990, the SII (per 100,000 population) of DALYs was −14.23 (95% CI: −15.83 to–12.63), and in 2021 it was–6.57 (95% CI: −7.38 to −5.75), indicating that the absolute inequality of DALYs between high SDI countries and low SDI countries has decreased during this period (Figure 5A). However, the concentration index revealed a rising trend in relative inequality between 1990 and 2021 (Figure 5B), reflecting that high SDI countries have made greater improvements in reducing the burden of rare diseases than countries with low SDI. Considering that purely epidemiological factors cannot fully explain these differences, we further analyzed the rare disease policies across countries at different levels of economic development to examine the role of policy effects.

#### 3.2.5 Policy effects on the disease burden

**Figure 6:**
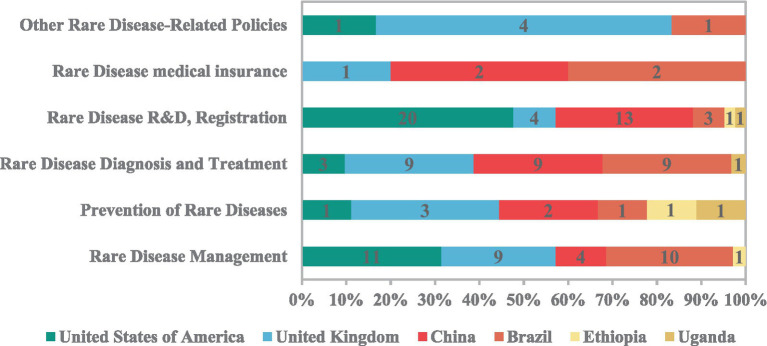
Comparison of comprehensive rare disease policies across countries at different development levels.

**Figure 7:**
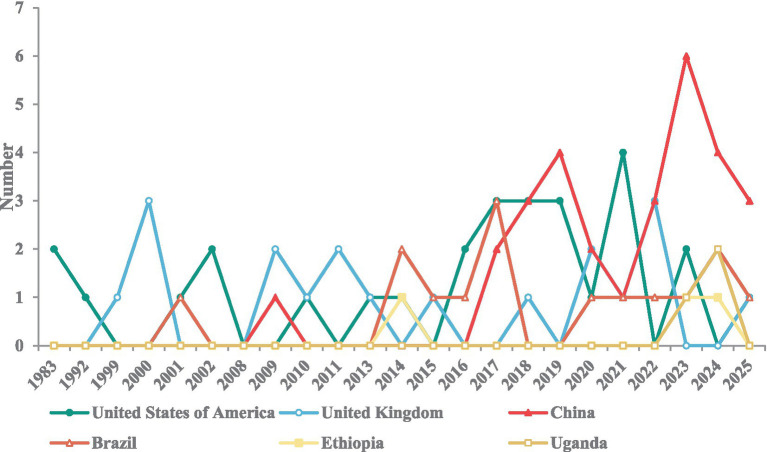
Trends in the release dates and number of rare disease policies across countries at different levels of development.

The policy comparison results revealed significant differences across countries at varying development levels in both the comprehensiveness of coverage across the rare disease policy continuum and the timing of policy implementation (Figures 6, 7; Supplementary Tables S10, S11). Overall, high-income countries introduced policies for rare diseases earlier and established a relatively comprehensive system covering all six areas. In contrast, middle-income countries demonstrated a marked catch-up trend, with the number of rare disease policies in China and Brazil increasing after 2016. Low-income countries, such as Ethiopia and Uganda, initiated rare disease policies later and with a narrower scope, introducing only fragmented supportive measures in a few areas, including rare disease management, prevention, diagnosis, and treatment, and R&D registration. This finding is consistent with the disease burden being predominantly concentrated in low SDI countries, alongside a persistent trend of widening relative inequality.

## 4 Discussion

Although there are differences in RB incidence, prevalence, DALYs, and death from country to country, the overall global RB burden has shown a trend of increasing and then gradually decreasing between 1990 and 2021. Projections suggest that although the number of incidence and prevalence is expected to decline slightly every year between 2022 and 2035, the number of DALYs and deaths will continue to rise. This suggests that the control and management of RB will remain a focus of global public health efforts in the coming decades. Transnational inequality analysis shows that low-SDI countries bear a disproportionately higher burden of rare diseases, and the relative inequality associated with SDI has intensified over time. In terms of rare disease policy formulation, countries with high and middle SDI have established more complete systems, while countries with low SDI still have limited scope and progress.

Although RB is a rare disease with a relatively low incidence and prevalence, its burden of disease is still quite heavy. For example, in 2021, the total DALYs reached 279,066 (95% confidence interval: 159,656–365,150). There are significant differences between different regions and countries, and sub-Saharan East Africa ranks first in all four indicators. India reported the highest number of incidence and deaths, while China has the highest prevalence and number of DALYs. The Republic of Malawi has the largest ASDR, whereas Tokelau has the largest ASIR, ASR-DALY, and ASPR. Regions with a relatively heavy burden of these diseases require more attention and targeted interventions. Analysis of SDI strata indicates that nations with low SDI experience a greater disease burden in terms of quantity. As far as ASR is concerned, low SDI deaths and DALYs are the highest, highlighting significant health inequalities associated with RB, which may be attributed to factors such as war (27), armed conflict, and economic crises (28, 29).

From an overall temporal perspective, global RB incidence and prevalence increased, whereas ASR-DALY and ASDR declined. With the exception of Sub-Saharan Africa, all other regions showed a decline in ASR-DALY, and ASDR decreased across all regions, with Central Europe showing the most significant downward trends. Furthermore, ASR-DALY decreased in 180 countries (88.23%), and ASDR decreased in 181 countries (88.73%). These widespread declines are closely linked to advances in RB diagnosis and treatment, as well as improvements in rare disease policy. Between 1990 and 2021, the widespread application of intravenous chemotherapy (30, 31), intra-arterial chemotherapy, intravitreal chemotherapy, and combined local radiotherapy significantly improved local control and eye preservation rates, leading to marked reductions in death and years lived with disability (32, 33). Declines in mortality and DALYs reflect not only medical progress but also the critical role of neonatal screening, referral systems, and treatment infrastructure established in recent years (34). Particularly in Central Europe, most countries gradually introduced neonatal red reflex or “leukocoria” screening in the late 20th and early 21st centuries (35), incorporating it into national child health programs. Combined with rare ocular tumor registries and multinational clinical research networks, these measures enabled standardized clinical guidelines and knowledge sharing (36). In addition, universal health insurance coverage reduced treatment abandonment due to financial barriers (37), thereby improving patient outcomes at a population level. By contrast, resource-limited regions such as Sub-Saharan Africa continue to experience high ASR-DALY/ASDR and poorer survival outcomes due to delayed diagnosis and limited treatment access, underscoring substantial policy and resource gaps (38).

Segmented trend analysis reveals that the growth rates of ASIR and ASPR varied from 1990 to 2003, 2003–2015, and 2015–2019, with the growth trend accelerating over time. Due to the genetic characteristics of RB, this trend may reflect the improvement of diagnostic and screening capabilities and the enhancement of public awareness, thus increasing the case detection rate rather than the real increase in the risk of disease (39). From 2019 to 2021, ASIR and ASPR decreased slightly, which may be due to external factors such as disruptions in healthcare services, delayed diagnosis, and underreporting during the COVID-19 pandemic affecting cancer detection and surveillance worldwide (40). In contrast, ASR-DALY and ASDR are generally on a downward trend, but their change patterns are more complex. Both indicators rose slightly from 2002 to 2014 but declined sharply after 2014, accelerating from 2019 to 2021. These changes are closely related to the adoption of multidisciplinary management models and the emergence of new local treatments, particularly intra-arterial chemotherapy and intravitreal chemotherapy, which have substantially improved survival outcomes as well as eye- and vision-preservation rates, thus effectively reducing the burden of disease caused by death and disability (33). Overall, the period between 1990 and the early 2000s was characterized by inadequate diagnostic and curative capacity, resulting in fluctuations or increases in the disease burden. In contrast, the past decade has shown a continuous downward trend, mainly due to advances in healthcare levels and resources. These changes highlight the importance of policies such as promoting early screening, simplifying referral processes, and centralized treatment.

Previous studies, using the Bayesian age-period-cohort (BAPC) model, have suggested that ASIR, ASPR, and ASR-DALY would show modest increases from 1990 to 2021, while ASDR may decline slightly (15). Extending this evidence, this study projected the number of incidence, prevalence, DALYs, and deaths for global RB through 2035. The results indicate that while the numbers of incidence and prevalence of RB may gradually decline, the numbers of DALYs and deaths are projected to continue increasing, highlighting the persistent global health burden of the disease. This differs somewhat from previous studies on the increase in ASIR and ASPR, primarily due to demographic changes, as the high-risk group—namely children—is expected to decrease due to persistently low fertility rates. Furthermore, this discrepancy suggests that future related projection research should focus not only on predicting ASR but also on predicting its number.

Crude rates can more directly reflect the actual burden of populations in different countries or regions (41). The inequality analysis using the crude DALYs rate showed that low SDI countries bear a more serious burden of rare diseases. Between 1990 and 2021, absolute inequality has narrowed, indicating that the global burden of rare diseases has generally improved, and the absolute gap between countries with high SDI and countries with low SDI has reduced. However, relative inequality has increased, because the improvement of countries with high SDI is faster than that of countries with low SDI, thus widening the proportional difference. This aligns with Li et al. (42) who similarly reported widening relative inequalities in ASR-DALY. Based on the comparison of rare disease policies, our research results show that high-income countries have launched rare disease policies earlier and established perfect screening, diagnosis, treatment capabilities and health care security systems. Middle-income countries are quickly following suit, while low-income countries are still lagging behind and independent. These studies suggest that health inequalities are partly due to inadequate policy coverage.

The use of novel therapeutic agents (e.g., topotecan) (43) and the optimization of treatment strategies [e.g., avoiding decentralized and overly aggressive treatment approaches (44)] have played a significant role in improving survival outcomes for patients with RB. However, existing research results show that RB patients have reduced survival rates. Research shows that medical progress must be combined with institutional and policy support to translate into practical patient benefits (45). The results of this study’s analysis of the effects of policy on disease burden indicate a clear association between policy development and trends in the burden of RB. Further integrating the findings of this study on regional trends in the burden of RB, we observed that from 1990 to 2021, ASIR and ASPR in high and high-middle SDI strata showed an increasing trend, while ASR-DALY and ASDR declined most markedly. This aligns with the policies of systematic neonatal screening, centralized treatment centers, national registration systems, and rare disease medical insurance policies in high-income countries, which enhance the timeliness and accessibility of medical services, ultimately improving patient prognosis. In contrast, the World Health Assembly resolution on rare diseases emphasized that inadequate screening items and uneven diagnostic resources may be the leading causes of delayed diagnosis (7, 8). Between 1990 and 2021, the ASIR and ASPR of other SDIs exhibited an upward trend, whereas the ASIR and ASPR of low SDI showed a downward trend. The decline in ASR-DALY and ASDR with low SDI is relatively small, which may reflect significant policy deficiencies, leading to limited screening, reduced treatment opportunities, and low affordability for medical expenses, thus exacerbating the structural root causes of health inequality (46). Therefore, the future RB prevention and control strategy should not only focus on promoting medical intervention, but also strengthen and implement public health policies. Systematic policy development and global collaboration (47, 48) are critical to narrowing regional disparities and sustaining a sustained decline in the global RB disease burden. Significantly, such policy improvements can not only improve the survival rate and quality of life of RB patients but also provide valuable experience for managing other rare diseases, thus helping to enhance the well-being of patients with rare diseases worldwide and promote health equity.

This study also has several limitations. First, it is based on the GBD database and may be affected by uncertainties and underreporting. Second, further rigorous causal inference studies on policy effects are needed in the future.

## 5 Conclusion

In summary, although RB is a rare tumor, it continues to impose a substantial global disease burden with pronounced regional disparities. From 1990 to 2021, incidence and prevalence exhibited an overall upward trend, while death and DALYs rates declined, suggesting improvements in diagnostic and therapeutic capacity. However, projection trends indicate that the number of DALYs and deaths will continue to rise in the coming decades, underscoring that RB prevention and control remain a critical global public health challenge. Notably, regions such as Central Europe have achieved significant reductions in disease burden through comprehensive policy frameworks and standardized management, highlighting the pivotal role of policy interventions and systemic measures in mitigating disease burden. In contrast, RB patients in low SDI countries continue to face disproportionate burdens due to limited resources and inadequate policy support, with health inequities remaining particularly acute. Moving forward, it is imperative to strengthen policy development related to rare diseases globally, especially in underdeveloped regions. Such policies and measures would narrow regional gaps, alleviate disease burden, and provide valuable lessons for the management of other rare diseases and the advancement of health equity.

## Data Availability

The original contributions presented in the study are included in the article/Supplementary material, further inquiries can be directed to the corresponding authors.
