# Supplementary material for: Cross-national inequalities and public health policy effects of the global retinoblastoma burden from 1990 to 2021 and projections to 2035
Source: Front Public Health. 2026 Jan 26;14:1753390. doi: 10.3389/fpubh.2026.1753390 (PMC12883818; doi:10.3389/fpubh.2026.1753390)
Supplement: Supplementary file 1 [file Table_1.DOCX]

Table S1 The incidence number and ASIR of retinoblastoma by region in 1990 and 2021, along with its EAPC

| **Location** | **1990** | | **2021** | | **EAPC (95 %CI)1990-2021** |
| --- | --- | --- | --- | --- | --- |
|  | **Number (95% UIs)** | **ASR (95% UIs)** | **Number (95% UIs)** | **ASR (95% UIs)** |  |
| **Global** | 4674(3033,5953) | 0.08 (0.05, 0.10) | 6275(3855, 8382) | 0.09 (0.06, 0.13) | 1.35 (1.13, 1.56) |
| **SDI strata** |  | | | | |
| High SDI | 818(684,962) | 0.13 (0.11, 0.15) | 649(489, 829) | 0.12 (0.09, 0.15) | 0.43 (0.1, 0.77) |
| High-middle SDI | 606(406,899) | 0.07 (0.04, 0.10) | 935(488, 1388) | 0.13 (0.07, 0.19) | 3.58 (3.1, 4.06) |
| Middle SDI | 907(520,1338) | 0.05 (0.03, 0.07) | 1481(848, 2090) | 0.08 (0.05, 0.11) | 2.8 (2.51, 3.1) |
| Low-middle SDI | 1198(659,1690) | 0.07 (0.04, 0.10) | 1531(881, 2241) | 0.08 (0.05, 0.12) | 0.74 (0.54, 0.94) |
| Low SDI | 1142(628,1573) | 0.13 (0.07, 0.18) | 1677(979, 2522) | 0.10 (0.06, 0.15) | -0.57 (-0.75, -0.4) |
| **GBD regions** |  | | | | |
| Andean Latin America | 55(31, 98) | 0.11 (0.06, 0.19) | 112(62, 188) | 0.18 (0.10, 0.30) | 2.63 (2.35, 2.91) |
| Australasia | 8(6, 10) | 0.05 (0.04, 0.07) | 3(1, 5) | 0.02 (0.01, 0.03) | -1.47 (-3.31, 0.4) |
| Caribbean | 14(9, 21) | 0.03 (0.02, 0.05) | 4(2, 7) | 0.01 (0.01, 0.02) | -2.12 (-3.2, -1.03) |
| Central Asia | 31(13, 66) | 0.03 (0.01, 0.07) | 55(28, 97) | 0.06 (0.03, 0.10) | 2.7 (2.26, 3.15) |
| Central Europe | 29(15, 55) | 0.03 (0.02, 0.06) | 18(11, 27) | 0.03 (0.02, 0.05) | -0.25 (-1.17, 0.68) |
| Central Latin America | 139(98, 190) | 0.06 (0.04, 0.08) | 161(105, 242) | 0.08 (0.05, 0.12) | 1.46 (0.8, 2.13) |
| Central Sub-Saharan Africa | 51(23, 86) | 0.05 (0.02, 0.09) | 72(26, 147) | 0.03 (0.01, 0.07) | -0.95 (-1.16, -0.75) |
| East Asia | 565(290, 927) | 0.05 (0.03, 0.08) | 1125(444, 1763) | 0.14 (0.06, 0.22) | 5.55 (4.81, 6.29) |
| Eastern Europe | 107(74, 156) | 0.06 (0.04, 0.09) | 52(38, 72) | 0.05 (0.04, 0.07) | -1.15 (-1.63, -0.68) |
| Eastern Sub-Saharan Africa | 972(564, 1357) | 0.28 (0.16, 0.39) | 1265(787, 1975) | 0.20 (0.12, 0.31) | -0.67 (-0.88, -0.45) |
| High-income Asia Pacific | 138(107, 176) | 0.13 (0.10, 0.16) | 107(74, 147) | 0.16 (0.11, 0.21) | 1.52 (0.64, 2.4) |
| High-income North America | 445(370, 529) | 0.21 (0.17, 0.25) | 233(159, 336) | 0.11 (0.08, 0.16) | -1.25 (-1.71, -0.78) |
| North Africa and Middle East | 114(67, 180) | 0.02 (0.01, 0.04) | 231(146, 359) | 0.04 (0.02, 0.06) | 1.96 (1.68, 2.24) |
| Oceania | 1(0, 4) | 0.01 (0.00, 0.04) | 4(1, 10) | 0.02 (0.01, 0.05) | 0.73 (0.39, 1.07) |
| South Asia | 954(453, 1417) | 0.06 (0.03, 0.09) | 1248(671, 1931) | 0.08 (0.04, 0.12) | 0.94 (0.53, 1.36) |
| Southeast Asia | 218(92, 393) | 0.04 (0.02, 0.07) | 311(172, 494) | 0.05 (0.03, 0.09) | 1.31 (1.18, 1.44) |
| Southern Latin America | 41(22, 72) | 0.08 (0.04, 0.14) | 26(15, 39) | 0.06 (0.03, 0.09) | -0.7 (-1.5, 0.11) |
| Southern Sub-Saharan Africa | 25(12, 41) | 0.03 (0.02, 0.06) | 52(20, 92) | 0.06 (0.02, 0.11) | 2.95 (2.27, 3.63) |
| Tropical Latin America | 89(61, 125) | 0.05 (0.03, 0.07) | 74(49, 105) | 0.04 (0.03, 0.06) | 0.06 (-0.45, 0.56) |
| Western Europe | 291(238, 351) | 0.13 (0.10, 0.15) | 364(264, 462) | 0.17 (0.12, 0.21) | 1.15 (0.54, 1.77) |
| Western Sub-Saharan Africa | 387(184, 557) | 0.11 (0.05, 0.16) | 760(291, 1260) | 0.10 (0.04, 0.16) | -0.38 (-0.59, -0.17) |

**Abbreviations**: ASR, age-standardized rate; RB, Retinoblastoma; EAPC, estimated annual percentage change; UIs, uncertainty intervals; CI, conﬁdence interval.

**Table S2** The number of prevalence and ASPR of RB by region in 1990 and 2021, along with its EAPC

| **Location** | **1990** | | **2021** | | **EAPC (95 %CI)1990-2021** |
| --- | --- | --- | --- | --- | --- |
|  | **Number (95% UIs)** | **ASR (95% UIs)** | **Number (95% UIs)** | **ASR (95% UIs)** |  |
| Global | 42550(27687,54220) | 0.69 (0.45, 0.88) | 57333(35247,76615) | 0.86 (0.53, 1.15) | 1.36 (1.15, 1.57) |
| **SDI strata** |  | | | | |
| High SDI | 7493(6268, 8805) | 1.20 (1.01, 1.41) | 5531(3707, 8219) | 1.07 (0.81, 1.37) | 0.44 (0.11, 0.77) |
| High-middle SDI | 5531(3707, 8219) | 0.59 (0.40, 0.88) | 8560(4466, 12687) | 1.19 (0.62, 1.78) | 3.59 (3.11, 4.07) |
| Middle SDI | 8273(4746, 12177) | 0.41 (0.24, 0.61) | 13551(7768, 19120) | 0.75 (0.42, 1.05) | 2.81 (2.52, 3.11) |
| Low-middle SDI | 10888(5990, 15344) | 0.64 (0.35, 0.90) | 13971(8043, 20462) | 0.73 (0.42, 1.06) | 2.81 (2.52, 3.11) |
| Low SDI | 10343(5690, 14273) | 1.18 (0.65, 1.63) | 15279(8925, 22943) | 0.93 (0.55, 1.40) | -0.55 (-0.72, -0.38) |
| **GBD regions** |  | | | | |
| Andean Latin America | 504(281, 892) | 0.97 (0.54, 1.72) | 1022(568, 1714) | 1.65 (0.92, 2.77) | 2.64 (2.36, 2.93) |
| Australasia | 75(55, 95) | 0.48 (0.36, 0.62) | 26(14, 47) | 0.14 (0.07, 0.25) | -1.47 (-3.31, 0.41) |
| Caribbean | 126(79, 190) | 0.31 (0.2, 0.47) | 40(22, 68) | 0.1 (0.06, 0.17) | -2.12 (-3.2, -1.03) |
| Central Asia | 287(118, 605) | 0.31 (0.13, 0.66) | 501(257, 889) | 0.51 (0.26, 0.9) | 2.71 (2.26, 3.16) |
| Central Europe | 261(134, 502) | 0.28 (0.14, 0.54) | 161(103, 250) | 0.28 (0.18, 0.44) | -0.24 (-1.17, 0.69) |
| Central Latin America | 1266(897, 1732) | 0.56 (0.4, 0.77) | 1474(958, 2215) | 0.72 (0.46, 1.08) | 1.47 (0.8, 2.13) |
| Central Sub-Saharan Africa | 462(209, 780) | 0.47 (0.21, 0.8) | 653(240, 1339) | 0.31 (0.11, 0.64) | -0.94 (-1.15, -0.73) |
| East Asia | 5151(2641, 8443) | 0.45 (0.23, 0.73) | 10297(4066, 16118) | 1.29 (0.51, 2.02) | 5.57 (4.83, 6.31) |
| Eastern Europe | 976(675, 1423) | 0.55 (0.38, 0.81) | 476(351, 658) | 0.44 (0.32, 0.61) | -1.14 (-1.62, -0.67) |
| Eastern Sub-Saharan Africa | 8814(5114, 12295) | 2.53 (1.47, 3.54) | 11533(7177, 17980) | 1.82 (1.13, 2.85) | -0.65 (-0.87, -0.43) |
| High-income Asia Pacific | 1268(983, 1615) | 1.18 (0.92, 1.5) | 979(679, 1347) | 1.44 (1, 1.97) | 1.52 (0.65, 2.4) |
| High-income North America | 4079(3390, 4845) | 1.9 (1.58, 2.26) | 2142(1462, 3082) | 1.01 (0.69, 1.45) | -1.24 (-1.71, -0.77) |
| North Africa and Middle East | 1044(613, 1644) | 0.21 (0.12, 0.32) | 2110(1333, 3285) | 0.34 (0.22, 0.53) | 1.97 (1.7, 2.25) |
| Oceania | 13(4, 36) | 0.14 (0.04, 0.37) | 32(9, 94) | 0.17 (0.05, 0.5) | 0.74 (0.4, 1.08) |
| South Asia | 8656(4110, 12853) | 0.55 (0.26, 0.82) | 11391(6129, 17628) | 0.71 (0.38, 1.1) | 0.97 (0.55, 1.38) |
| Southeast Asia | 1987(840, 3583) | 0.34 (0.14, 0.62) | 2847(1571, 4515) | 0.5 (0.27, 0.79) | 1.32 (1.19, 1.45) |
| Southern Latin America | 374(199, 656) | 0.73 (0.39, 1.27) | 238(140, 360) | 0.52 (0.31, 0.79) | -0.69 (-1.5, 0.12) |
| Southern Sub-Saharan Africa | 226(111, 377) | 0.31 (0.15, 0.51) | 471(184, 831) | 0.58 (0.23, 1.03) | 2.95 (2.27, 3.63) |
| Tropical Latin America | 811(563, 1144) | 0.46 (0.32, 0.65) | 680(446, 964) | 0.39 (0.26, 0.56) | 0.07 (-0.44, 0.57) |
| Western Europe | 2666(2180, 3216) | 1.15 (0.94, 1.38) | 3336(2418, 4239) | 1.53 (1.11, 1.93) | 1.16 (0.55, 1.77) |
| Western Sub-Saharan Africa | 3504(1668, 5071) | 1.03 (0.49, 1.5) | 6923(2644, 11472) | 0.88 (0.34, 1.46) | -0.36 (-0.57, -0.15) |

**Abbreviations**: ASR, age-standardized rate; RB, Retinoblastoma; EAPC, estimated annual percentage change; UIs, uncertainty intervals; CI, conﬁdence interval.

**Table S3** The number of deaths and ASDR of RB by region in 1990 and 2021, along with its EAPC

| **Location** | **1990** | | **2021** | | **EAPC (95 %CI)1990-2021** |
| --- | --- | --- | --- | --- | --- |
|  | **Number (95% UIs)** | **ASR (95% UIs)** | **Number (95% UIs)** | **ASR (95% UIs)** |  |
| **Global** | 3180(1819,4173) | 0.05 (0.03, 0.07) | 2762(1666,3761) | 0.04 (0.03, 0.06) | -0.44 (-0.55, -0.33) |
| **SDI strata** |  | | | | |
| High SDI | 40(33, 47) | 0.01 (0.01, 0.01) | 12(10, 15) | 0 (0, 0) | -2.64 (-3.13, -2.15) |
| High-middle SDI | 214(120, 327) | 0.02 (0.01, 0.04) | 59(30, 86) | 0.01 (0, 0.01) | -2.77 (-3.09, -2.46) |
| Middle SDI | 652(386, 861) | 0.03 (0.02, 0.04) | 309(175, 417) | 0.02 (0.01, 0.02) | -1.77 (-1.96, -1.59) |
| Low-middle SDI | 1134(613, 1583) | 0.07 (0.04, 0.09) | 923(553, 1277) | 0.05 (0.03, 0.07) | -0.69 (-0.8, -0.58) |
| Low SDI | 1140(634, 1571) | 0.13 (0.07, 0.18) | 1457(879, 2101) | 0.09 (0.05, 0.13) | -0.95 (-1.09, -0.81) |
| **GBD regions** |  | | | | |
| Andean Latin America | 48(28, 82) | 0.09 (0.05, 0.16) | 26(16, 43) | 0.04 (0.03, 0.07) | -2.06 (-2.23, -1.89) |
| Australasia | 0(0, 0) | 0 (0, 0) | 0(0, 0) | 0 (0, 0) | -4.55 (-6.26, -2.81) |
| Caribbean | 6(3, 8) | 0.01 (0.01, 0.02) | 2(1, 3) | 0 (0, 0.01) | -2.29 (-2.99, -1.59) |
| Central Asia | 16(7, 30) | 0.02 (0.01, 0.03) | 14(8, 25) | 0.01 (0.01, 0.03) | -0.21 (-0.43, 0) |
| Central Europe | 9(4, 17) | 0.01 (0, 0.02) | 1(1, 2) | 0 (0, 0) | -5.14 (-5.87, -4.39) |
| Central Latin America | 93(79, 109) | 0.04 (0.04, 0.05) | 38(26, 54) | 0.02 (0.01, 0.03) | -2.14 (-2.52, -1.75) |
| Central Sub-Saharan Africa | 51(23, 87) | 0.05 (0.02, 0.09) | 66(26, 126) | 0.03 (0.01, 0.06) | -1.14 (-1.39, -0.89) |
| East Asia | 320(166, 494) | 0.03 (0.01, 0.04) | 74(32, 111) | 0.01 (0, 0.01) | -2.47 (-2.96, -1.97) |
| Eastern Europe | 24(18, 33) | 0.01 (0.01, 0.02) | 5(4, 6) | 0 (0, 0.01) | -4.61 (-5.36, -3.86) |
| Eastern Sub-Saharan Africa | 943(554, 1328) | 0.27 (0.16, 0.38) | 1058(700, 1629) | 0.17 (0.11, 0.26) | -1.02 (-1.25, -0.79) |
| High-income Asia Pacific | 8(6, 12) | 0.01 (0.01, 0.01) | 2(1, 2) | 0 (0, 0) | -3.08 (-3.79, -2.36) |
| High-income North America | 14(13, 16) | 0.01 (0.01, 0.01) | 4(3, 6) | 0 (0, 0) | -3.01 (-3.64, -2.38) |
| North Africa and Middle East | 78(47, 123) | 0.02 (0.01, 0.02) | 44(27, 70) | 0.01 (0, 0.01) | -2.3 (-2.64, -1.97) |
| Oceania | 1(0, 4) | 0.01 (0, 0.04) | 3(1, 9) | 0.02 (0, 0.05) | 0.42 (0.04, 0.79) |
| South Asia | 936(441, 1396) | 0.06 (0.03, 0.09) | 674(378, 1003) | 0.04 (0.02, 0.06) | -1.11 (-1.29, -0.93) |
| Southeast Asia | 155(67, 257) | 0.03 (0.01, 0.04) | 96(42, 145) | 0.02 (0.01, 0.03) | -1.39 (-1.48, -1.31) |
| Southern Latin America | 8(5, 12) | 0.01 (0.01, 0.02) | 2(1, 2) | 0 (0, 0) | -4.22 (-4.89, -3.55) |
| Southern Sub-Saharan Africa | 19(10, 30) | 0.03 (0.01, 0.04) | 31(13, 56) | 0.04 (0.02, 0.07) | 1.94 (1.52, 2.35) |
| Tropical Latin America | 64(50, 80) | 0.04 (0.03, 0.05) | 20(13, 27) | 0.01 (0.01, 0.02) | -3.02 (-3.47, -2.57) |
| Western Europe | 11(13, 310) | 0 (0, 0.01) | 5(4, 6) | 0 (0, 0) | -2.43 (-3.37, -1.47) |
| Western Sub-Saharan Africa | 376(179, 544) | 0.11 (0.05, 0.16) | 599(252, 949) | 0.08 (0.03, 0.12) | -1.04 (-1.19, -0.89) |

**Abbreviations**: ASR, age-standardized rate; RB, Retinoblastoma; EAPC, estimated annual percentage change; UIs, uncertainty intervals; CI, conﬁdence interval.

**Table S4** The incidence number and ASIR for RB by country in 1990 and 2021, along with its EAPC

| **Location** | **1990** | | **2021** | | **EAPC**  **(95% CI) 1990-2021** |
| --- | --- | --- | --- | --- | --- |
|  | **Number (95% UIs)** | **ASR (95% UIs)** | **Number (95% UIs)** | **ASR (95% UIs)** |  |
| Afghanistan | 0（0,0） | 0.0（0.0,0.0） | 0（0,2） | 0.0（0.0,0.0） | 0.18(-0.03,0.40) |
| Albania | 0（0,0） | 0.0（0.0,0.0） | 0（0,0） | 0.0（0.0,0.01） | 5.72(4.60,6.85) |
| Algeria | 13（6,26） | 0.04（0.02,0.07） | 23（10,45） | 0.05（0.02,0.1） | 0.90(0.53,1.27) |
| American Samoa | 0（0,0） | 0.01（0.0,0.02） | 0（0,0） | 0.01（0.0,0.03） | 1.23(0.82,1.63) |
| Andorra | 0（0,0） | 0.04（0.01,0.07） | 0（0,0） | 0.03（0.01,0.06） | -0.72(-1.27,-0.16) |
| Angola | 10（5,24） | 0.06（0.03,0.13） | 21（7,50） | 0.04（0.01,0.09） | -1.34(-1.48,-1.21) |
| Antigua and Barbuda | 0（0,0） | 0.01（0.0,0.02） | 0（0,0） | 0.0（0.0,0.0） | -7.50(-9.30,-5.66) |
| Argentina | 29（14,56） | 0.09（0.04,0.16） | 13（6,22） | 0.04（0.02,0.07） | -1.42(-2.24,-0.59) |
| Armenia | 0（0,0） | 0.0（0.0,0.0） | 0（0,1） | 0.05（0.02,0.1） | 18.07(14.74,21.49) |
| Australia | 7（5,9） | 0.06（0.04,0.08） | 2（1,4） | 0.02（0.01,0.03） | -1.64(-3.29,0.04) |
| Austria | 6（4,9） | 0.15（0.1,0.2） | 6（3,10） | 0.14（0.07,0.23） | 0.78(0.02,1.55) |
| Azerbaijan | 3（0,9） | 0.04（0.01,0.11） | 5（1,17） | 0.08（0.02,0.22） | 2.96(2.72,3.20) |
| Bahamas | 0（0,0） | 0.05（0.02,0.1） | 0（0,0） | 0.03（0.01,0.07） | -0.54(-2.25,1.20) |
| Bahrain | 0（0,0） | 0.0（0.0,0.01） | 0（0,0） | 0.02（0.0,0.05） | 8.13(6.84,9.44) |
| Bangladesh | 137（63,269） | 0.07（0.03,0.15） | 138（64,288） | 0.09（0.04,0.2） | 1.04(0.46,1.62) |
| Barbados | 0（0,0） | 0.08（0.03,0.21） | 0（0,0） | 0.19（0.07,0.42） | 4.11(2.88,5.35) |
| Belarus | 5（2,10） | 0.07（0.03,0.14） | 9（2,24） | 0.19（0.05,0.48） | 3.43(1.99,4.88) |
| Belgium | 8（5,11） | 0.14（0.09,0.2） | 9（5,15） | 0.16（0.09,0.25） | 0.70(-0.19,1.60) |
| Belize | 0（0,0） | 0.04（0.01,0.08） | 0（0,0） | 0.01（0.0,0.01） | -5.31(-7.27,-3.30) |
| Benin | 12（4,27） | 0.13（0.05,0.29） | 22（8,53） | 0.1（0.04,0.24） | -0.87(-1.08,-0.65) |
| Bermuda | 0（0,0） | 0.0（0.0,0.0） | 0（0,0） | 0.0（0.0,0.0） | 7.69(3.89,11.61) |
| Bhutan | 0（0,1） | 0.06（0.02,0.16） | 0（0,1） | 0.11（0.05,0.23） | 2.05(1.70,2.40) |
| Bolivia (Plurinational State of) | 12（5,26） | 0.13（0.06,0.27） | 17（7,37） | 0.15（0.06,0.31） | 0.53(0.16,0.91) |
| Bosnia and Herzegovina | 0（0,0） | 0.0（0.0,0.01） | 0（0,0） | 0.0（0.0,0.01） | 1.70(0.52,2.88) |
| Botswana | 0（0,1） | 0.03（0.01,0.06） | 1（0,2） | 0.05（0.02,0.12） | 2.28(1.92,2.63) |
| Brazil | 84（58,120） | 0.05（0.03,0.07） | 66（43,93） | 0.04（0.03,0.06） | -0.10(-0.64,0.44) |
| Brunei Darussalam | 0（0,0） | 0.21（0.09,0.28） | 0（0,0） | 0.26（0.19,0.29） | 0.94(0.83,1.05) |
| Bulgaria | 0（0,1） | 0.02（0.01,0.04） | 0（0,1） | 0.02（0.0,0.04） | -0.80(-1.73,0.13) |
| Burkina Faso | 23（9,54） | 0.13（0.05,0.31） | 35（14,80） | 0.09（0.04,0.2） | -0.92(-1.34,-0.49) |
| Burundi | 24（11,46） | 0.24（0.11,0.45） | 26（10,57） | 0.12（0.05,0.27） | -1.03(-1.55,-0.50) |
| Côte d'Ivoire | 6（2,16） | 0.07（0.02,0.17） | 12（4,30） | 0.18（0.08,0.39） | 3.87(3.23,4.52) |
| Cabo Verde | 0（0,0） | 0.02（0.0,0.04） | 0（0,1） | 0.02（0.0,0.05） | 0.25(-0.16,0.67) |
| Cambodia | 2（0,6） | 0.1（0.04,0.23） | 3（0,8） | 0.08（0.03,0.19） | -0.33(-0.54,-0.12) |
| Cameroon | 19（7,44） | 0.2（0.14,0.28） | 38（13,92） | 0.11（0.06,0.19） | -1.11(-1.45,-0.76) |
| Canada | 38（26,53） | 0.06（0.03,0.14） | 21（11,37） | 0.06（0.02,0.14） | -0.00(-0.19,0.18) |
| Central African Republic | 3（1,6） | 0.11（0.04,0.25） | 4（1,11） | 0.09（0.04,0.19） | -0.58(-0.66,-0.50) |
| Chad | 13（5,29） | 0.05（0.03,0.08） | 31（13,66） | 0.08（0.04,0.14） | 1.00(-0.27,2.29) |
| Chile | 6（3,11） | 0.05（0.03,0.08） | 9（4,16） | 0.14（0.05,0.22） | 5.55(4.81,6.30) |
| China | 551（279,907） | 0.06（0.03,0.08） | 1102（428,1737） | 0.1（0.06,0.18） | 4.65(3.47,5.83) |
| Colombia | 23（13,34） | 0.22（0.1,0.47） | 36（20,61） | 0.22（0.08,0.49） | -0.15(-0.40,0.10) |
| Comoros | 1（0,3） | 0.05（0.02,0.12） | 1（0,3） | 0.04（0.01,0.1） | -0.85(-1.10,-0.60) |
| Congo | 2（0,4） | 0.03（0.01,0.09） | 2（0,6） | 0.29（0.07,0.94） | 4.02(2.51,5.55) |
| Cook Islands | 0（0,0） | 0.13（0.08,0.22） | 0（0,0） | 0.08（0.05,0.15） | -0.10(-0.83,0.64) |
| Costa Rica | 5（3,8） | 0.03（0.01,0.07） | 2（1,4） | 0.03（0.01,0.07） | 0.15(-0.23,0.54) |
| Croatia | 0（0,0） | 0.01（0.0,0.01） | 0（0,0） | 0.01（0.0,0.02） | 1.12(-1.30,3.61) |
| Cuba | 6（3,11） | 0.08（0.04,0.13） | 0（0,0） | 0.0（0.0,0.01） | -6.84(-8.99,-4.65) |
| Cyprus | 0（0,0） | 0.0（0.0,0.01） | 0（0,0） | 0.01（0.0,0.03） | 7.72(6.77,8.67) |
| Czechia | 1（1,2） | 0.03（0.02,0.04） | 2（1,4） | 0.04（0.02,0.09） | 1.74(0.33,3.17) |
| Democratic People's Republic of Korea | 7（3,16） | 0.03（0.02,0.07） | 10（4,24） | 0.07（0.03,0.16） | 2.73(2.51,2.95) |
| Democratic Republic of the Congo | 33（14,64） | 0.05（0.02,0.09） | 40（15,97） | 0.03（0.01,0.07） | -0.99(-1.28,-0.71) |
| Denmark | 7（5,9） | 0.25（0.18,0.34） | 8（5,11） | 0.26（0.17,0.38） | 0.65(-0.20,1.51) |
| Djibouti | 1（0,2） | 0.25（0.11,0.46） | 2（0,6） | 0.2（0.07,0.48） | -0.38(-0.73,-0.03) |
| Dominica | 0（0,0） | 0.0（0.0,0.0） | 0（0,0） | 0.0（0.0,0.0） | 1.56(1.03,2.09) |
| Dominican Republic | 1（0,3） | 0.02（0.01,0.03） | 1（0,3） | 0.02（0.01,0.04） | 1.30(0.49,2.12) |
| Ecuador | 8（4,14） | 0.06（0.03,0.11） | 30（15,54） | 0.18（0.09,0.32） | 4.33(3.95,4.71) |
| Egypt | 4（1,13） | 0.01（0.0,0.02） | 12（3,39） | 0.01（0.0,0.03） | 2.17(0.48,3.89) |
| El Salvador | 1（0,3） | 0.02（0.01,0.04） | 1（0,4） | 0.03（0.01,0.07） | 1.83(1.31,2.35) |
| Equatorial Guinea | 0（0,1） | 0.06（0.02,0.13） | 0（0,2） | 0.04（0.01,0.11） | -1.47(-2.04,-0.90) |
| Eritrea | 15（5,30） | 0.25（0.09,0.52） | 20（7,43） | 0.22（0.08,0.48） | -0.50(-0.75,-0.26) |
| Estonia | 0（0,0） | 0.04（0.02,0.08） | 0（0,0） | 0.04（0.02,0.07） | 1.51(0.19,2.85) |
| Eswatini | 0（0,0） | 0.03（0.01,0.06） | 0（0,1） | 0.04（0.02,0.09） | 1.13(0.94,1.31) |
| Ethiopia | 132（58,251） | 0.14（0.06,0.27） | 142（71,261） | 0.09（0.05,0.17） | -1.90(-2.42,-1.37) |
| Fiji | 0（0,0） | 0.02（0.0,0.05） | 0（0,0） | 0.03（0.0,0.08） | 1.89(1.44,2.33) |
| Finland | 5（3,6） | 0.16（0.11,0.22） | 9（6,13） | 0.37（0.23,0.51） | 2.87(2.47,3.28) |
| France | 70（51,91） | 0.18（0.13,0.23） | 90（53,136） | 0.25（0.15,0.37） | 1.81(0.94,2.68) |
| Gabon | 0（0,1） | 0.03（0.01,0.07） | 0（0,2） | 0.04（0.01,0.11） | 1.23(0.88,1.57) |
| Gambia | 1（0,4） | 0.11（0.05,0.24） | 4（1,11） | 0.13（0.05,0.34） | 0.56(0.15,0.97) |
| Georgia | 0（0,1） | 0.01（0.0,0.03） | 2（0,6） | 0.09（0.02,0.24） | 10.74(9.40,12.10) |
| Germany | 49（31,76） | 0.11（0.07,0.17） | 46（25,77） | 0.11（0.06,0.19） | 0.63(0.26,1.00) |
| Ghana | 21（8,49） | 0.08（0.03,0.2） | 35（11,92） | 0.08（0.02,0.2） | 0.02(-0.41,0.46) |
| Greece | 1（1,2） | 0.03（0.02,0.04） | 2（1,3） | 0.05（0.02,0.09） | 2.78(1.49,4.09) |
| Greenland | 0（0,0） | 0.04（0.01,0.1） | 0（0,0） | 0.02（0.01,0.06） | 0.31(-0.82,1.45) |
| Grenada | 0（0,0） | 0.0（0.0,0.01） | 0（0,0） | 0.0（0.0,0.0） | -5.37(-7.43,-3.27) |
| Guam | 0（0,0） | 0.02（0.01,0.07） | 0（0,0） | 0.03（0.01,0.11） | 3.06(2.35,3.77) |
| Guatemala | 6（2,13） | 0.04（0.02,0.09） | 7（3,14） | 0.05（0.02,0.09） | 1.11(0.86,1.36) |
| Guinea | 25（11,52） | 0.23（0.11,0.48） | 31（12,74） | 0.14（0.05,0.34） | -1.17(-1.40,-0.94) |
| Guinea-Bissau | 2(1,6) | 0.15（0.06,0.37） | 2（1,5） | 0.08（0.03,0.18） | -1.45(-1.91,-0.99) |
| Guyana | 0（0,0） | 0.0（0.0,0.01） | 0（0,0） | 0.02（0.01,0.03） | 4.47(1.18,7.88) |
| Haiti | 0（0,1） | 0.01（0.0,0.02） | 0（0,1） | 0.0（0.0,0.01） | -1.59(-1.74,-1.43) |
| Honduras | 3（1,6） | 0.04（0.02,0.09） | 4（2,11） | 0.04（0.02,0.1） | 0.38(0.27,0.48) |
| Hungary | 1（0,2） | 0.03（0.02,0.04） | 2（1,4） | 0.05（0.02,0.09） | 3.23(1.53,4.96) |
| Iceland | 0（0,0） | 0.21（0.15,0.27） | 0（0,0） | 0.24（0.2,0.28） | 0.80(0.44,1.17) |
| India | 698（321,1061） | 0.06（0.03,0.09） | 809（439,1274） | 0.07（0.04,0.11） | 0.61(0.12,1.10) |
| Indonesia | 25（6,45） | 0.01（0.0,0.02） | 36（8,72） | 0.02（0.0,0.03） | 1.48(1.39,1.58) |
| Iran (Islamic Republic of) | 1（0,4） | 0.0（0.0,0.0） | 2（0,4） | 0.0（0.0,0.01） | 4.14(3.48,4.80) |
| Iraq | 9（4,18） | 0.03（0.01,0.06） | 44（21,84） | 0.1（0.05,0.19） | 4.28(4.07,4.49) |
| Ireland | 2（1,4） | 0.09（0.06,0.13） | 2（1,4） | 0.08（0.04,0.14） | -0.01(-0.43,0.41) |
| Israel | 3（1,4） | 0.06（0.03,0.1） | 9（5,15） | 0.11（0.06,0.17） | 3.02(2.38,3.66) |
| Italy | 34（27,41） | 0.12（0.1,0.15） | 47（33,60） | 0.21（0.15,0.27） | 0.75(-0.46,1.99) |
| Jamaica | 1（0,3） | 0.05（0.02,0.13） | 0（0,1） | 0.02（0.01,0.06） | -2.23(-3.71,-0.73) |
| Japan | 94（77,114） | 0.14（0.11,0.16） | 49（33,69） | 0.1（0.07,0.15） | 0.37(-0.90,1.65) |
| Jordan | 1（0,3） | 0.03（0.01,0.06） | 6（3,10） | 0.05（0.03,0.09） | 2.07(1.72,2.43) |
| Kazakhstan | 14（3,39） | 0.08（0.02,0.21） | 12（3,34） | 0.07（0.02,0.18） | 0.62(0.21,1.04) |
| Kenya | 252（131,388） | 0.6（0.31,0.93） | 303（160,496） | 0.51（0.27,0.83） | 0.00(-0.40,0.41) |
| Kiribati | 0（0,0） | 0.02（0.0,0.06） | 0（0,0） | 0.02（0.0,0.05） | -1.07(-1.42,-0.71) |
| Kuwait | 1（1,2） | 0.09（0.06,0.14） | 0（0,0） | 0.01（0.0,0.01） | -7.33(-11.47,-2.99) |
| Kyrgyzstan | 3（1,10） | 0.06（0.02,0.17） | 7（2,16） | 0.09（0.03,0.21） | 5.26(2.61,7.98) |
| Lao People's Democratic Republic | 1（0,2） | 0.02（0.0,0.04） | 1（0,3） | 0.01（0.0,0.04） | -0.68(-0.84,-0.51) |
| Latvia | 1（0,1） | 0.05（0.02,0.1） | 0（0,0） | 0.01（0.0,0.01） | -6.93(-7.31,-6.54) |
| Lebanon | 0（0,1） | 0.02（0.01,0.04） | 2（1,4） | 0.06（0.02,0.11） | 3.80(3.41,4.19) |
| Lesotho | 0（0,1） | 0.03（0.01,0.07） | 0（0,1） | 0.04（0.02,0.09） | 1.18(0.92,1.43) |
| Liberia | 4（1,10） | 0.1（0.04,0.25） | 5（1,15） | 0.07（0.03,0.2） | -0.90(-1.71,-0.08) |
| Libya | 3（1,8） | 0.06（0.03,0.13） | 3（1,8） | 0.09（0.04,0.19） | 1.22(0.84,1.59) |
| Lithuania | 1（0,2） | 0.05（0.03,0.09） | 0（0,0） | 0.0（0.0,0.01） | -7.37(-8.73,-6.00) |
| Luxembourg | 0（0,0） | 0.01（0.01,0.02） | 0（0,0） | 0.01（0.0,0.04） | 1.92(0.58,3.28) |
| Madagascar | 44（19,86） | 0.21（0.09,0.42） | 65（24,149） | 0.16（0.06,0.37） | -0.83(-1.00,-0.67) |
| Malawi | 99（48,191） | 0.55（0.27,1.06） | 118（46,242） | 0.44（0.17,0.89） | -0.37(-0.63,-0.11) |
| Malaysia | 9（2,21） | 0.04（0.01,0.09） | 15（5,34） | 0.06（0.02,0.14） | 2.12(1.71,2.54) |
| Maldives | 0（0,0） | 0.0（0.0,0.01） | 0（0,0） | 0.01（0.01,0.02） | 4.60(4.02,5.18) |
| Mali | 40（17,86） | 0.25（0.11,0.54） | 56（19,163） | 0.13（0.04,0.37） | -1.89(-2.09,-1.70) |
| Malta | 0（0,0） | 0.01（0.01,0.02） | 0（0,0） | 0.01（0.01,0.02） | 0.91(-0.07,1.89) |
| Marshall Islands | 0（0,0） | 0.01（0.0,0.03） | 0（0,0） | 0.02（0.0,0.05） | 1.34(1.04,1.63) |
| Mauritania | 3（1,8） | 0.09（0.04,0.23） | 6（2,15） | 0.1（0.04,0.23） | -0.03(-0.37,0.31) |
| Mauritius | 0（0,0） | 0.0（0.0,0.01） | 0（0,0） | 0.01（0.01,0.03） | 7.21(3.70,10.83) |
| Mexico | 78（54,112） | 0.07（0.05,0.1） | 83（50,125） | 0.08（0.05,0.12） | 0.27(-0.49,1.03) |
| Micronesia (Federated States of) | 0（0,0） | 0.01（0.0,0.05） | 0（0,0） | 0.02（0.0,0.06） | 1.11(0.85,1.37) |
| Monaco | 0（0,0） | 0.01（0.01,0.02） | 0（0,0） | 0.04（0.02,0.09） | 1.74(0.77,2.72) |
| Mongolia | 0（0,2） | 0.02（0.0,0.06） | 2（0,7） | 0.07（0.02,0.18） | 4.59(3.95,5.25) |
| Montenegro | 0（0,0） | 0.01（0.0,0.02） | 0（0,0） | 0.0（0.0,0.01） | 0.25(-0.47,0.97) |
| Morocco | 40（18,81） | 0.12（0.05,0.23） | 65（26,130） | 0.2（0.08,0.4） | 2.24(2.06,2.42) |
| Mozambique | 80（38,162） | 0.35（0.17,0.7） | 110（49,208） | 0.22（0.1,0.41） | -1.09(-1.37,-0.82) |
| Myanmar | 9（2,24） | 0.02（0.0,0.05） | 11（2,31） | 0.02（0.0,0.06） | 0.25(-0.04,0.54) |
| Namibia | 2（0,4） | 0.1（0.04,0.21） | 6（2,13） | 0.23（0.1,0.5） | 3.34(2.67,4.01) |
| Nauru | 0（0,0） | 0.02（0.0,0.05） | 0（0,0） | 0.03（0.01,0.08） | 1.11(0.94,1.29) |
| Nepal | 21（9,49） | 0.07（0.03,0.16） | 22（9,49） | 0.07（0.03,0.16） | 0.56(0.14,0.98) |
| Netherlands | 21（15,27） | 0.23（0.17,0.3） | 24（14,34） | 0.28（0.17,0.4） | 0.87(0.23,1.52) |
| New Zealand | 0（0,1） | 0.03（0.01,0.05） | 0（0,0） | 0.01（0.0,0.03） | -0.21(-3.53,3.23) |
| Nicaragua | 6（2,18） | 0.1（0.04,0.29） | 5（2,11） | 0.08（0.04,0.17） | 0.74(0.21,1.26) |
| Niger | 24（10,57） | 0.15（0.06,0.37） | 39（15,96） | 0.08（0.03,0.2） | -2.06(-2.34,-1.78) |
| Nigeria | 155（69,244） | 0.1（0.05,0.16） | 393（129,716） | 0.11（0.04,0.2） | 0.18(-0.02,0.37) |
| Niue | 0（0,0） | 0.03（0.01,0.1） | 0（0,0） | 0.22（0.05,0.76） | 3.25(2.04,4.48) |
| North Macedonia | 0（0,0） | 0.0（0.0,0.0） | 0（0,0） | 0.0（0.0,0.0） | 3.34(2.21,4.48) |
| Northern Mariana Islands | 0（0,0） | 0.01（0.0,0.02） | 0（0,0） | 0.01（0.0,0.02） | -0.52(-1.29,0.26) |
| Norway | 0（0,1） | 0.03（0.03,0.04） | 0（0,0） | 0.0（0.0,0.01） | -4.74(-6.94,-2.49) |
| Oman | 0（0,1） | 0.02（0.01,0.05） | 1（0,2） | 0.03（0.01,0.07） | 1.95(1.37,2.54) |
| Pakistan | 94（43,177） | 0.05（0.02,0.1） | 276（119,501） | 0.09（0.04,0.17） | 2.43(2.25,2.61) |
| Palau | 0（0,0） | 0.02（0.0,0.06） | 0（0,0） | 0.03（0.01,0.11） | 1.65(1.35,1.95) |
| Palestine | 0（0,0） | 0.01（0.0,0.01） | 0（0,1） | 0.01（0.0,0.02） | 2.87(2.12,3.62) |
| Panama | 2（0,4） | 0.08（0.03,0.17） | 4（2,8） | 0.12（0.06,0.22） | 1.41(1.24,1.59) |
| Papua New Guinea | 0（0,3） | 0.01（0.0,0.05） | 2（0,8） | 0.02（0.0,0.06） | 0.62(0.24,0.99) |
| Paraguay | 4（1,9） | 0.07（0.03,0.15） | 7（2,16） | 0.11（0.04,0.25） | 2.15(1.87,2.43) |
| Peru | 34（17,67） | 0.12（0.06,0.24） | 63（26,132） | 0.19（0.08,0.4） | 2.73(2.35,3.11) |
| Philippines | 35（9,69） | 0.04（0.01,0.08） | 70（19,123） | 0.06（0.02,0.11） | 1.85(1.61,2.08) |
| Poland | 0（0,0） | 0.0（0.0,0.0） | 0（0,1） | 0.0（0.0,0.01） | 0.21(-2.77,3.27) |
| Portugal | 20（13,28） | 0.34（0.23,0.46） | 18（12,23） | 0.44（0.3,0.54） | 1.33(0.19,2.48) |
| Puerto Rico | 0（0,1） | 0.03（0.02,0.06） | 0（0,0） | 0.01（0.0,0.01） | -3.50(-5.99,-0.95) |
| Qatar | 0（0,0） | 0.0（0.0,0.0） | 0（0,0） | 0.0（0.0,0.0） | 0.74(-2.35,3.93) |
| Republic of Korea | 35（16,63） | 0.1（0.05,0.18） | 46（26,75） | 0.28（0.16,0.45） | 3.70(3.19,4.20) |
| Republic of Moldova | 5（2,14） | 0.13（0.05,0.32） | 0（0,1） | 0.05（0.02,0.11） | -3.49(-4.22,-2.76) |
| Romania | 19（6,45） | 0.11（0.04,0.25） | 7（3,15） | 0.08（0.04,0.17） | -1.73(-2.51,-0.95) |
| Russian Federation | 78（52,112） | 0.07（0.04,0.09） | 31（23,42） | 0.04（0.03,0.05） | -2.49(-3.15,-1.82) |
| Rwanda | 36（17,74） | 0.28（0.13,0.57） | 28（10,61） | 0.16（0.06,0.35） | -1.54(-1.87,-1.21) |
| Saint Kitts and Nevis | 0（0,0） | 0.0（0.0,0.0） | 0（0,0） | 0.0（0.0,0.0） | -5.26(-7.18,-3.30) |
| Saint Lucia | 0（0,0） | 0.0（0.0,0.01） | 0（0,0） | 0.0（0.0,0.0） | -1.88(-3.25,-0.50) |
| Saint Vincent and the Grenadines | 0（0,0） | 0.19（0.07,0.45） | 0（0,0） | 0.02（0.01,0.04） | -6.48(-8.30,-4.63) |
| Samoa | 0（0,0） | 0.03（0.01,0.08） | 0（0,0） | 0.05（0.01,0.12） | 0.85(0.61,1.08) |
| San Marino | 0（0,0） | 0.05（0.02,0.11） | 0（0,0） | 0.04（0.01,0.09） | -0.16(-0.36,0.04) |
| Sao Tome and Principe | 0（0,0） | 0.11（0.04,0.27） | 0（0,0） | 0.12（0.04,0.3） | 0.81(-0.44,2.09) |
| Saudi Arabia | 6（0,15） | 0.03（0.0,0.06） | 8（1,21） | 0.04（0.01,0.09） | 2.18(0.39,3.99) |
| Senegal | 18（7,44） | 0.13（0.05,0.32） | 23（8,57） | 0.1（0.04,0.26） | -0.22(-0.68,0.23) |
| Serbia | 1（0,4） | 0.03（0.01,0.06） | 0（0,1） | 0.02（0.01,0.04） | -1.25(-1.89,-0.59) |
| Seychelles | 0（0,0） | 0.0（0.0,0.0） | 0（0,0） | 0.0（0.0,0.0） | 1.38(1.03,1.74) |
| Sierra Leone | 7（3,17） | 0.11（0.04,0.24） | 10（3,26） | 0.08（0.03,0.2） | -0.87(-1.16,-0.57) |
| Singapore | 7（5,9） | 0.37（0.26,0.48） | 10（6,13） | 0.35（0.21,0.48） | 0.18(-0.32,0.67) |
| Slovakia | 0（0,1） | 0.02（0.01,0.04） | 1（0,3） | 0.05（0.02,0.11） | 4.49(3.40,5.59) |
| Slovenia | 0（0,0） | 0.03（0.02,0.05） | 0（0,1） | 0.09（0.04,0.17） | 4.76(3.78,5.76) |
| Solomon Islands | 0（0,0） | 0.01（0.0,0.04） | 0（0,0） | 0.02（0.0,0.06） | 0.81(0.16,1.46) |
| Somalia | 35（14,75） | 0.24（0.1,0.52） | 57（17,139） | 0.14（0.04,0.35） | -1.03(-1.50,-0.56) |
| South Africa | 13（5,27） | 0.03（0.01,0.06） | 28（9,58） | 0.06（0.02,0.12） | 2.73(1.49,3.98) |
| South Sudan | 20（8,40） | 0.21（0.09,0.41） | 21（9,42） | 0.14（0.06,0.28） | -0.51(-0.77,-0.26) |
| Spain | 40（30,53） | 0.18（0.14,0.24） | 49（28,74） | 0.25（0.15,0.39） | 1.39(0.53,2.25) |
| Sri Lanka | 4（1,9） | 0.02（0.01,0.05） | 6（2,14） | 0.04（0.02,0.09） | 3.32(2.71,3.93) |
| Sudan | 0（0,1） | 0.0（0.0,0.0） | 1（0,3） | 0.0（0.0,0.01） | 1.54(1.19,1.90) |
| Suriname | 0（0,0） | 0.0（0.0,0.0） | 0（0,0） | 0.0（0.0,0.0） | 1.94(1.28,2.60) |
| Sweden | 0（0,0） | 0.0（0.0,0.01） | 0（0,0） | 0.01（0.0,0.01） | 3.33(-0.67,7.49) |
| Switzerland | 9（6,13） | 0.24（0.17,0.34） | 15（9,21） | 0.35（0.21,0.49） | 0.98(0.64,1.32) |
| Syrian Arab Republic | 0（0,0） | 0.0（0.0,0.0） | 0（0,0） | 0.0（0.0,0.0） | 2.14(1.54,2.76) |
| Taiwan | 4（3,6） | 0.03（0.02,0.04） | 11（6,19） | 0.13（0.07,0.22） | 6.66(5.52,7.80) |
| Tajikistan | 1（0,2） | 0.01（0.0,0.03） | 3（0,8） | 0.02（0.0,0.07） | 2.96(2.70,3.23) |
| Thailand | 26（12,46） | 0.05（0.02,0.09） | 14（6,30） | 0.05（0.02,0.1） | 0.17(-0.47,0.81) |
| Timor-Leste | 0(0,0) | 0.01（0.0,0.03） | 0（0,0） | 0.02（0.0,0.04） | 0.63(0.25,1.01) |
| Togo | 6（2,15） | 0.1（0.04,0.23） | 9（3,24） | 0.08（0.03,0.21） | -0.72(-0.96,-0.49) |
| Tokelau | 0(0,0) | 0.01（0.0,0.03） | 0（0,0） | 1.19（0.25,3.86） | 6.60(2.86,10.49) |
| Tonga | 0（0,0） | 0.02（0.0,0.05） | 0（0,0） | 0.03（0.01,0.11） | 2.12(1.75,2.50) |
| Trinidad and Tobago | 0（0,1） | 0.05（0.02,0.12） | 0（0,1） | 0.09（0.04,0.2） | 3.47(2.13,4.83) |
| Tunisia | 9（4,18） | 0.08（0.04,0.17） | 10（4,18） | 0.11（0.05,0.21） | 0.93(0.64,1.21) |
| Türkiye | 19（8,36） | 0.03（0.01,0.05） | 45（25,80） | 0.08（0.04,0.14） | 3.82(3.25,4.40) |
| Turkmenistan | 0（0,2） | 0.02（0.01,0.04） | 1（0,3） | 0.02（0.01,0.07） | 1.42(1.31,1.53) |
| Tuvalu | 0（0,0） | 0.02（0.0,0.05） | 0（0,0） | 0.02（0.0,0.07） | 0.93(0.57,1.30) |
| Uganda | 71（32,136） | 0.21（0.09,0.4） | 125（51,297） | 0.17（0.07,0.41） | -0.40(-0.73,-0.06) |
| Ukraine | 14（7,29） | 0.04（0.02,0.08） | 9（4,16） | 0.05（0.02,0.09） | 1.63(1.17,2.08) |
| United Arab Emirates | 0（0,0） | 0.01（0.0,0.03） | 0（0,2） | 0.02（0.01,0.05） | 3.97(3.18,4.76) |
| United Kingdom | 7（6,8） | 0.02（0.02,0.02） | 20（16,25） | 0.06（0.04,0.07） | 1.68(0.38,3.00) |
| United Republic of Tanzania | 114（47,241） | 0.25（0.1,0.52） | 191（72,430） | 0.22（0.08,0.49） | 0.10(-0.17,0.36) |
| United States of America | 406（337,480） | 0.21（0.17,0.25） | 211（144,303） | 0.11（0.08,0.16） | -1.26(-1.75,-0.77) |
| United States Virgin Islands | 0（0,0） | 0.08（0.03,0.19） | 0（0,0） | 0.06（0.02,0.13） | -1.44(-1.93,-0.94) |
| Uruguay | 4（2,7） | 0.16（0.08,0.28） | 3（1,5） | 0.15（0.08,0.25） | -0.37(-0.69,-0.04) |
| Uzbekistan | 7（1,21） | 0.02（0.01,0.07） | 18（5,40） | 0.05（0.02,0.11） | 3.22(2.71,3.73) |
| Vanuatu | 0（0,0） | 0.01（0.0,0.03） | 0（0,0） | 0.01（0.0,0.04） | 1.14(0.47,1.82) |
| Venezuela (Bolivarian Republic of) | 12（5,23） | 0.05（0.02,0.1） | 14（6,30） | 0.06（0.03,0.14） | 1.61(1.07,2.16) |
| Vietnam | 102（45,206） | 0.11（0.05,0.22） | 149（62,303） | 0.18（0.08,0.36） | 1.55(1.37,1.72) |
| Yemen | 0（0,0） | 0.0（0.0,0.0） | 0（0,2） | 0.0（0.0,0.0） | 1.83(1.46,2.20) |
| Zambia | 38（18,74） | 0.26（0.12,0.53） | 47（20,101） | 0.16（0.07,0.35） | -1.49(-1.70,-1.29) |
| Zimbabwe | 6（3,13） | 0.04（0.02,0.08） | 13（4,32） | 0.06（0.02,0.15） | 4.10(2.58,5.63) |

**Abbreviations**: ASR, age-standardized rate; RB, Retinoblastoma; EAPC, estimated annual percentage change; UIs, uncertainty intervals; CI, conﬁdence interval.

**Table S5** The number of Prevalence and ASPR for RB by country in 1990 and 2021, along with its EAPC

| **Location** | **1990** | | **2021** | | **EAPC (95% CI) 1990-2021** |
| --- | --- | --- | --- | --- | --- |
|  | **Number (95% UIs)** | **ASR (95% UIs)** | **Number (95% UIs)** | **ASR (95% UIs)** |  |
| Afghanistan | 1（0,4） | 0.01（0.0,0.03） | 5（0,19） | 0.01（0.0,0.04） | 0.21(-0.01,0.42) |
| Albania | 0（0,0） | 0.0（0.0,0.01） | 0（0,0） | 0.02（0.0,0.05） | 5.73(4.61,6.86) |
| Algeria | 122（58,238） | 0.33（0.16,0.63） | 215（98,414） | 0.46（0.21,0.88） | 0.91(0.54,1.29) |
| American Samoa | 0（0,0） | 0.05（0.01,0.15） | 0（0,0） | 0.08（0.02,0.28） | 1.23(0.83,1.63) |
| Andorra | 0（0,0） | 0.35（0.13,0.68） | 0（0,0） | 0.23（0.08,0.55） | -0.71(-1.26,-0.16) |
| Angola | 99（46,218） | 0.55（0.26,1.18） | 198（70,454） | 0.36（0.13,0.82） | -1.32(-1.45,-1.19) |
| Antigua and Barbuda | 0（0,0） | 0.06（0.02,0.17） | 0（0,0） | 0.01（0.0,0.01） | -7.50(-9.30,-5.66) |
| Argentina | 272（132,520） | 0.79（0.38,1.51） | 122（60,204） | 0.38（0.19,0.64） | -1.41(-2.23,-0.58) |
| Armenia | 0（0,0） | 0.01（0.0,0.03） | 8（3,17） | 0.44（0.17,0.91） | 18.07(14.74,21.50) |
| Australia | 67（48,88） | 0.53（0.38,0.69） | 23（11,38） | 0.15（0.08,0.25） | -1.64(-3.29,0.04) |
| Austria | 61（43,84） | 1.37（0.96,1.87） | 55（28,94） | 1.28（0.66,2.17） | 0.79(0.02,1.55) |
| Azerbaijan | 29（7,90） | 0.33（0.09,1.02） | 53（12,158） | 0.7（0.16,2.05） | 2.97(2.73,3.21) |
| Bahamas | 1（0,2） | 0.44（0.2,0.88） | 0（0,1） | 0.27（0.1,0.68） | -0.54(-2.26,1.20) |
| Bahrain | 0（0,0） | 0.04（0.02,0.08） | 1（0,4） | 0.17（0.03,0.43） | 8.14(6.85,9.45) |
| Bangladesh | 1246（574,2444） | 0.67（0.31,1.34） | 1264（590,2634） | 0.86（0.41,1.8） | 1.07(0.49,1.65) |
| Barbados | 1（0,3） | 0.76（0.28,1.9） | 2（0,5） | 1.75（0.63,3.82） | 4.11(2.89,5.35) |
| Belarus | 48（23,100） | 0.6（0.28,1.24） | 88（24,221） | 1.77（0.48,4.41） | 3.44(2.00,4.89) |
| Belgium | 74（48,109） | 1.24（0.81,1.82） | 88（50,139） | 1.44（0.83,2.26） | 0.70(-0.18,1.60) |
| Belize | 0（0,2） | 0.32（0.13,0.7） | 0（0,0） | 0.05（0.02,0.11） | -5.31(-7.27,-3.31) |
| Benin | 110（43,246） | 1.17（0.45,2.62） | 203（73,491） | 0.89（0.32,2.15） | -0.85(-1.07,-0.64) |
| Bermuda | 0（0,0） | 0.0（0.0,0.0） | 0（0,0） | 0.0（0.0,0.0） | 7.69(3.90,11.63) |
| Bhutan | 5（1,13） | 0.57（0.15,1.43） | 6（2,12） | 1.0（0.43,2.08） | 2.07(1.72,2.42) |
| Bolivia (Plurinational State of) | 114（50,244） | 1.17（0.51,2.48） | 162（66,340） | 1.36（0.56,2.85） | 0.54(0.17,0.92) |
| Bosnia and Herzegovina | 1（0,3） | 0.04（0.01,0.1） | 0（0,1） | 0.04（0.01,0.12） | 1.70(0.53,2.89) |
| Botswana | 5（2,11） | 0.28（0.11,0.53） | 11（4,26） | 0.49（0.17,1.14） | 2.27(1.91,2.64) |
| Brazil | 770（537,1099） | 0.45（0.32,0.65） | 611（402,860） | 0.37（0.24,0.52） | -0.09(-0.63,0.45) |
| Brunei Darussalam | 6（2,8） | 1.93（0.86,2.61） | 7（5,8） | 2.42（1.75,2.61） | 0.95(0.84,1.06) |
| Bulgaria | 8（3,17） | 0.16（0.06,0.32） | 4（1,12） | 0.15（0.04,0.4） | -0.80(-1.73,0.14) |
| Burkina Faso | 210（86,495） | 1.17（0.48,2.78） | 323（133,734） | 0.81（0.33,1.85） | -0.90(-1.33,-0.47) |
| Burundi | 223（105,420） | 2.15（1.02,4.08） | 240（99,518） | 1.13（0.46,2.44） | -1.00(-1.53,-0.47) |
| Côte d'Ivoire | 59（25,145） | 0.62（0.2,1.55） | 112（39,281） | 1.63（0.69,3.56） | 3.88(3.24,4.53) |
| Cabo Verde | 3（1,9） | 0.15（0.04,0.33） | 7（3,15） | 0.17（0.04,0.43） | 0.28(-0.14,0.69) |
| Cambodia | 25（6,57） | 0.92（0.36,2.12） | 29（6,74） | 0.73（0.25,1.75） | -0.32(-0.53,-0.11) |
| Cameroon | 176（70,407） | 1.82（1.25,2.53） | 350（120,844） | 1.0（0.52,1.76） | -1.10(-1.44,-0.76) |
| Canada | 352（242,486） | 0.59（0.27,1.24） | 198（103,349） | 0.55（0.21,1.22） | 0.01(-0.18,0.19) |
| Central African Republic | 27（13,58） | 1.01（0.41,2.28） | 45（17,99） | 0.83（0.35,1.72） | -0.57(-0.65,-0.49) |
| Chad | 117（47,264） | 0.44（0.23,0.76） | 287（122,599） | 0.76（0.38,1.3） | 1.01(-0.26,2.30) |
| Chile | 62（32,107） | 0.45（0.23,0.74） | 87（43,151） | 1.3（0.5,2.05） | 5.57(4.83,6.32) |
| China | 5032（2547,8269） | 0.51（0.31,0.77） | 10091（3924,15885） | 0.95（0.54,1.61） | 4.65(3.48,5.84) |
| Colombia | 210（126,316） | 2.03（0.92,4.27） | 332（187,559） | 2.04（0.76,4.44） | -0.14(-0.39,0.11) |
| Comoros | 16（7,33） | 0.48（0.2,1.08） | 16（6,35） | 0.35（0.11,0.88） | -0.84(-1.09,-0.58) |
| Congo | 18（7,41） | 0.23（0.05,0.83） | 22（7,56） | 2.68（0.63,8.61） | 4.03(2.52,5.56) |
| Cook Islands | 0（0,0） | 1.21（0.73,2.02） | 0（0,0） | 0.78（0.44,1.35） | -0.09(-0.82,0.64) |
| Costa Rica | 48（29,81） | 0.27（0.11,0.65） | 24（14,43） | 0.26（0.09,0.66） | 0.16(-0.23,0.55) |
| Croatia | 2（1,3） | 0.06（0.04,0.1） | 1（0,3） | 0.08（0.03,0.17） | 1.13(-1.30,3.61) |
| Cuba | 63（34,104） | 0.72（0.4,1.19） | 1（0,3） | 0.03（0.02,0.06） | -6.84(-8.99,-4.64) |
| Cyprus | 0（0,0） | 0.02（0.01,0.05） | 0（0,1） | 0.11（0.04,0.27） | 7.72(6.78,8.68) |
| Czechia | 15（9,22） | 0.24（0.15,0.34） | 22（9,45） | 0.39（0.17,0.8） | 1.75(0.34,3.18) |
| Democratic People's Republic of Korea | 72（31,153） | 0.32（0.14,0.66） | 99（39,224） | 0.66（0.26,1.48） | 2.74(2.52,2.97) |
| Democratic Republic of the Congo | 307（132,583） | 0.44（0.19,0.85） | 371（137,884） | 0.28（0.1,0.66） | -0.98(-1.26,-0.70) |
| Denmark | 65（46,89） | 2.28（1.61,3.12） | 74（46,107） | 2.4（1.51,3.45） | 0.66(-0.19,1.52) |
| Djibouti | 14（6,25） | 2.29（1.02,4.17） | 26（9,63） | 1.85（0.62,4.37） | -0.37(-0.72,-0.02) |
| Dominica | 0（0,0） | 0.0（0.0,0.0） | 0（0,0） | 0.0（0.0,0.0） | 1.55(1.03,2.08) |
| Dominican Republic | 14（5,30） | 0.15（0.05,0.31） | 14（5,32） | 0.14（0.05,0.32） | 1.31(0.50,2.13) |
| Ecuador | 76（41,133） | 0.58（0.31,1.01） | 276（140,493） | 1.63（0.83,2.91） | 4.34(3.96,4.72) |
| Egypt | 42（13,120） | 0.05（0.02,0.15） | 113（34,364） | 0.09（0.03,0.28） | 2.19(0.50,3.91) |
| El Salvador | 12（5,30） | 0.16（0.07,0.41） | 16（7,38） | 0.27（0.12,0.63） | 1.84(1.32,2.36) |
| Equatorial Guinea | 3（1,9） | 0.51（0.22,1.2） | 7（1,19） | 0.37（0.08,1.02） | -1.45(-2.02,-0.88) |
| Eritrea | 135（49,278） | 2.25（0.82,4.66） | 185（64,393） | 2.05（0.71,4.34） | -0.48(-0.73,-0.23) |
| Estonia | 4（2,8） | 0.39（0.19,0.69） | 2（1,4） | 0.33（0.17,0.6） | 1.52(0.20,2.86) |
| Eswatini | 4（1,8） | 0.3（0.12,0.58） | 5（2,11） | 0.39（0.15,0.82） | 1.12(0.93,1.31) |
| Ethiopia | 1195（528,2269） | 1.29（0.57,2.44） | 1299（651,2384） | 0.83（0.41,1.52） | -1.87(-2.38,-1.34) |
| Fiji | 1（0,4） | 0.14（0.02,0.43） | 2（0,6） | 0.24（0.03,0.72） | 1.89(1.45,2.34) |
| Finland | 46（32,63） | 1.46（1.01,1.99） | 88（55,120） | 3.38（2.11,4.63） | 2.87(2.47,3.28) |
| France | 646（475,842） | 1.65（1.22,2.14） | 834（487,1251） | 2.28（1.33,3.4） | 1.81(0.95,2.68) |
| Gabon | 4（1,10） | 0.31（0.11,0.68） | 8（2,21） | 0.39（0.1,1.0） | 1.23(0.89,1.58) |
| Gambia | 17（7,37） | 0.99（0.42,2.16） | 41（15,108） | 1.19（0.43,3.09） | 0.57(0.16,0.98) |
| Georgia | 3（0,12） | 0.08（0.02,0.27） | 20（5,55） | 0.83（0.21,2.23） | 10.75(9.40,12.10) |
| Germany | 456（286,699） | 1.02（0.64,1.57） | 422（233,710） | 1.04（0.57,1.75） | 0.63(0.26,1.01) |
| Ghana | 193（74,452） | 0.76（0.29,1.78） | 323（105,839） | 0.7（0.23,1.82） | 0.03(-0.40,0.47) |
| Greece | 15（9,24） | 0.25（0.15,0.4） | 19（9,35） | 0.45（0.23,0.82） | 2.78(1.49,4.09) |
| Greenland | 0（0,0） | 0.39（0.08,0.91） | 0（0,0） | 0.23（0.06,0.55） | 0.32(-0.80,1.46) |
| Grenada | 0（0,0） | 0.03（0.01,0.05） | 0（0,0） | 0.0（0.0,0.01） | -5.38(-7.43,-3.27) |
| Guam | 0（0,1） | 0.21（0.05,0.66） | 0（0,1） | 0.28（0.07,1.05） | 3.06(2.35,3.78) |
| Guatemala | 59（25,124） | 0.4（0.17,0.83） | 69（35,131） | 0.44（0.22,0.82） | 1.13(0.88,1.37) |
| Guinea | 227（106,479） | 2.06（0.96,4.37） | 286（110,685） | 1.3（0.5,3.1） | -1.16(-1.39,-0.93) |
| Guinea-Bissau | 24(10,59) | 1.36（0.56,3.33） | 22（9,53） | 0.7（0.28,1.65） | -1.43(-1.89,-0.97) |
| Guyana | 0（0,0） | 0.02（0.01,0.05） | 1（0,2） | 0.14（0.06,0.3） | 4.48(1.19,7.88) |
| Haiti | 7（2,16） | 0.07（0.02,0.16） | 6（2,15） | 0.04（0.01,0.1） | -1.59(-1.74,-1.44) |
| Honduras | 29（14,62） | 0.37（0.18,0.78） | 44（18,101） | 0.41（0.17,0.92） | 0.39(0.29,0.50) |
| Hungary | 14（9,22） | 0.23（0.14,0.35） | 20（9,39） | 0.45（0.2,0.87） | 3.24(1.54,4.96) |
| Iceland | 4（2,5） | 1.9（1.37,2.5） | 4（3,5） | 2.18（1.82,2.55） | 0.80(0.44,1.17) |
| India | 6340（2922,9657） | 0.55（0.25,0.83） | 7395（4016,11637） | 0.66（0.36,1.04） | 0.63(0.15,1.12) |
| Indonesia | 231（60,417） | 0.1（0.03,0.18） | 336（78,660） | 0.15（0.04,0.3） | 1.49(1.40,1.59) |
| Iran (Islamic Republic of) | 14（1,37） | 0.02（0.0,0.04） | 19（3,40） | 0.03（0.01,0.06） | 4.14(3.48,4.81) |
| Iraq | 83（39,169） | 0.27（0.13,0.55） | 403（199,777） | 0.92（0.45,1.78） | 4.29(4.08,4.50) |
| Ireland | 25（17,37） | 0.85（0.58,1.22） | 22（11,39） | 0.72（0.37,1.28） | -0.00(-0.42,0.41) |
| Israel | 27（16,45） | 0.53（0.31,0.88） | 90（52,142） | 0.99（0.58,1.56） | 3.03(2.39,3.67) |
| Italy | 315（255,380） | 1.13（0.92,1.36） | 438（307,555） | 1.92（1.35,2.44） | 0.76(-0.46,1.99) |
| Jamaica | 13（4,33） | 0.5（0.17,1.18） | 3（1,9） | 0.21（0.07,0.55） | -2.23(-3.70,-0.73) |
| Japan | 868（705,1051） | 1.24（1.01,1.5） | 452（306,640） | 0.94（0.64,1.33） | 0.37(-0.90,1.66) |
| Jordan | 16（7,30） | 0.27（0.13,0.51） | 56（28,98） | 0.5（0.26,0.87） | 2.08(1.72,2.44) |
| Kazakhstan | 128（31,358） | 0.69（0.17,1.94） | 118（32,318） | 0.62（0.17,1.66） | 0.63(0.22,1.05) |
| Kenya | 2300（1198,3538） | 5.48（2.87,8.45） | 2767（1467,4525） | 4.62（2.45,7.56） | 0.01(-0.40,0.42) |
| Kiribati | 0（0,0） | 0.16（0.04,0.53） | 0（0,0） | 0.14（0.03,0.41） | -1.06(-1.42,-0.70) |
| Kuwait | 16（10,25） | 0.8（0.52,1.24） | 1（1,2） | 0.06（0.04,0.11） | -7.33(-11.47,-2.99) |
| Kyrgyzstan | 35（9,95） | 0.56（0.16,1.52） | 66（23,154） | 0.83（0.29,1.92） | 5.27(2.62,7.99) |
| Lao People's Democratic Republic | 10（2,27） | 0.16（0.04,0.4） | 10（2,28） | 0.13（0.03,0.35） | -0.65(-0.81,-0.48) |
| Latvia | 9（4,17） | 0.47（0.23,0.89） | 0（0,1） | 0.06（0.02,0.12） | -6.92(-7.31,-6.53) |
| Lebanon | 6（2,15） | 0.18（0.07,0.39） | 21（9,40） | 0.51（0.22,0.98） | 3.81(3.42,4.20) |
| Lesotho | 7（2,14） | 0.31（0.12,0.6） | 8（3,17） | 0.39（0.16,0.85） | 1.17(0.91,1.42) |
| Liberia | 39（16,98） | 0.89（0.36,2.22） | 48（17,138） | 0.64（0.23,1.83） | -0.88(-1.69,-0.06) |
| Libya | 33（15,74） | 0.53（0.24,1.17） | 36（16,77） | 0.82（0.36,1.71） | 1.22(0.85,1.60) |
| Lithuania | 12（6,24） | 0.45（0.23,0.86） | 0（0,0） | 0.03（0.02,0.07） | -7.37(-8.72,-5.99) |
| Luxembourg | 0（0,0） | 0.09（0.05,0.16） | 0（0,1） | 0.11（0.03,0.33） | 1.92(0.57,3.28) |
| Madagascar | 403（172,779） | 1.92（0.82,3.74） | 593（222,1366） | 1.45（0.55,3.35） | -0.81(-0.97,-0.65) |
| Malawi | 904（441,1735） | 4.95（2.44,9.62） | 1078（423,2211） | 3.97（1.56,8.14） | -0.35(-0.61,-0.09) |
| Malaysia | 84（27,197） | 0.36（0.12,0.84） | 145（54,312） | 0.59（0.22,1.25） | 2.13(1.72,2.54) |
| Maldives | 0（0,0） | 0.03（0.01,0.07） | 0（0,0） | 0.1（0.05,0.21） | 4.61(4.03,5.20) |
| Mali | 362（157,781） | 2.24（0.96,4.83） | 516（172,1490） | 1.17（0.39,3.37） | -1.88(-2.07,-1.68) |
| Malta | 0（0,0） | 0.1（0.06,0.14） | 0（0,0） | 0.12（0.06,0.2） | 0.91(-0.07,1.89) |
| Marshall Islands | 0（0,0） | 0.09（0.02,0.28） | 0（0,0） | 0.15（0.03,0.49） | 1.34(1.05,1.64) |
| Mauritania | 30（12,74） | 0.86（0.34,2.09） | 60（23,136） | 0.92（0.35,2.09） | -0.01(-0.35,0.32) |
| Mauritius | 0（0,0） | 0.05（0.02,0.09） | 0（0,1） | 0.13（0.07,0.24） | 7.20(3.70,10.82) |
| Mexico | 714（499,1022） | 0.62（0.43,0.88） | 762（457,1148） | 0.75（0.45,1.14） | 0.28(-0.48,1.04) |
| Micronesia (Federated States of) | 0（0,0） | 0.13（0.03,0.43） | 0（0,0） | 0.15（0.03,0.54） | 1.12(0.86,1.38) |
| Monaco | 0（0,0） | 0.11（0.05,0.21） | 0（0,0） | 0.4（0.16,0.87） | 1.75(0.78,2.72) |
| Mongolia | 7（1,18） | 0.22（0.04,0.56） | 25（7,65） | 0.65（0.2,1.65） | 4.60(3.95,5.25) |
| Montenegro | 0（0,0） | 0.06（0.02,0.16） | 0（0,0） | 0.03（0.01,0.08） | 0.25(-0.47,0.97) |
| Morocco | 373（168,748） | 1.06（0.48,2.15） | 596（244,1189） | 1.83（0.75,3.67） | 2.26(2.08,2.43) |
| Mozambique | 731（351,1474） | 3.15（1.5,6.38） | 1007（455,1900） | 1.96（0.89,3.7） | -1.08(-1.36,-0.81) |
| Myanmar | 87（21,218） | 0.17（0.04,0.44） | 107（22,289） | 0.21（0.04,0.55） | 0.27(-0.02,0.56) |
| Namibia | 19（8,41） | 0.89（0.4,1.89） | 57（26,126） | 2.08（0.94,4.56） | 3.34(2.67,4.01) |
| Nauru | 0（0,0） | 0.15（0.04,0.47） | 0（0,0） | 0.23（0.05,0.73） | 1.12(0.94,1.29) |
| Nepal | 199（89,453） | 0.63（0.28,1.43） | 201（83,452） | 0.65（0.27,1.46） | 0.58(0.16,1.00) |
| Netherlands | 197（145,252） | 2.12（1.57,2.72） | 221（135,316） | 2.54（1.56,3.63） | 0.88(0.24,1.52) |
| New Zealand | 7（3,13） | 0.26（0.13,0.49） | 3（0,8） | 0.1（0.03,0.28） | -0.20(-3.53,3.24) |
| Nicaragua | 57（21,171） | 0.88（0.33,2.61） | 50（23,103） | 0.77（0.36,1.56） | 0.75(0.22,1.27) |
| Niger | 217（93,524） | 1.38（0.59,3.37） | 359（144,882） | 0.73（0.29,1.8） | -2.04(-2.32,-1.76) |
| Nigeria | 1413（623,2213） | 0.95（0.42,1.49） | 3584（1177,6539） | 0.98（0.32,1.8） | 0.19(-0.00,0.39) |
| Niue | 0（0,0） | 0.27（0.06,0.92） | 0（0,0） | 1.99（0.48,6.93） | 3.25(2.04,4.48) |
| North Macedonia | 0（0,0） | 0.0（0.0,0.01） | 0（0,0） | 0.0（0.0,0.01） | 3.35(2.22,4.49) |
| Northern Mariana Islands | 0（0,0） | 0.06（0.01,0.21） | 0（0,0） | 0.05（0.01,0.16） | -0.52(-1.29,0.26) |
| Norway | 7（6,9） | 0.29（0.23,0.36） | 1（0,1） | 0.04（0.02,0.05） | -4.74(-6.94,-2.49) |
| Oman | 6（2,14） | 0.2（0.07,0.45） | 13（5,27） | 0.31（0.12,0.64） | 1.95(1.37,2.54) |
| Pakistan | 864（397,1615） | 0.47（0.22,0.89） | 2523（1087,4566） | 0.85（0.37,1.54） | 2.44(2.26,2.61) |
| Palau | 0（0,0） | 0.16（0.04,0.54） | 0（0,0） | 0.28（0.07,0.99） | 1.66(1.36,1.96) |
| Palestine | 2（0,4） | 0.06（0.02,0.13） | 6（2,13） | 0.11（0.05,0.22） | 2.88(2.13,3.63) |
| Panama | 21（8,45） | 0.75（0.31,1.59） | 40（20,77） | 1.08（0.55,2.02） | 1.42(1.24,1.60) |
| Papua New Guinea | 8（1,27） | 0.13（0.03,0.44） | 23（5,81） | 0.16（0.03,0.55） | 0.63(0.25,1.00) |
| Paraguay | 40（14,84） | 0.67（0.25,1.38） | 68（25,152） | 1.04（0.38,2.3） | 2.15(1.88,2.43) |
| Peru | 312（157,618） | 1.08（0.55,2.15） | 583（238,1208） | 1.77（0.72,3.67） | 2.74(2.36,3.12) |
| Philippines | 324（81,631） | 0.36（0.09,0.7） | 648（175,1127） | 0.57（0.16,1.0） | 1.86(1.62,2.09) |
| Poland | 6（4,8） | 0.02（0.02,0.03） | 7（4,13） | 0.04（0.02,0.07） | 0.21(-2.77,3.28) |
| Portugal | 188（126,257） | 3.09（2.09,4.2） | 173（116,214） | 4.03（2.71,4.97） | 1.33(0.20,2.48) |
| Puerto Rico | 8（4,16） | 0.27（0.14,0.53） | 0（0,1） | 0.07（0.04,0.12） | -3.50(-5.99,-0.95) |
| Qatar | 0（0,0） | 0.0（0.0,0.0） | 0（0,0） | 0.0（0.0,0.0） | 0.75(-2.35,3.94) |
| Republic of Korea | 322（151,592） | 0.92（0.43,1.66） | 426（239,697） | 2.55（1.44,4.13） | 3.70(3.20,4.21) |
| Republic of Moldova | 50（18,129） | 1.16（0.42,2.95） | 7（3,15） | 0.47（0.2,0.96） | -3.48(-4.21,-2.75) |
| Romania | 178（61,412） | 1.0（0.34,2.31） | 73（34,144） | 0.76（0.36,1.52） | -1.72(-2.50,-0.94) |
| Russian Federation | 714（481,1028） | 0.6（0.4,0.87） | 290（215,391） | 0.36（0.27,0.49） | -2.48(-3.14,-1.81) |
| Rwanda | 332（156,681） | 2.54（1.2,5.19） | 260（94,559） | 1.5（0.54,3.23） | -1.43(-1.83,-1.04) |
| Saint Kitts and Nevis | 0（0,0） | 0.0（0.0,0.0） | 0（0,0） | 0.0（0.0,0.0） | -5.26(-7.19,-3.30) |
| Saint Lucia | 0（0,0） | 0.02（0.01,0.05） | 0（0,0） | 0.01（0.0,0.02） | -1.89(-3.25,-0.50) |
| Saint Vincent and the Grenadines | 2（0,5） | 1.69（0.67,4.07） | 0（0,0） | 0.19（0.07,0.41） | -6.48(-8.29,-4.62) |
| Samoa | 0（0,1） | 0.3（0.1,0.73） | 1（0,3） | 0.41（0.13,1.14） | 0.85(0.62,1.09) |
| San Marino | 0（0,0） | 0.43（0.17,1.01） | 0（0,0） | 0.39（0.13,0.83） | -0.15(-0.35,0.05) |
| Sao Tome and Principe | 2（0,4） | 1.01（0.35,2.46） | 2（0,6） | 1.13（0.38,2.73） | 0.83(-0.43,2.10) |
| Saudi Arabia | 57（6,138） | 0.24（0.03,0.58） | 82（16,195） | 0.33（0.07,0.79） | 2.19(0.41,4.00) |
| Senegal | 166（67,400） | 1.19（0.47,2.87） | 213（77,525） | 0.95（0.35,2.34） | -0.21(-0.67,0.25) |
| Serbia | 18（7,40） | 0.26（0.1,0.59） | 6（2,14） | 0.18（0.07,0.39） | -1.24(-1.89,-0.59) |
| Seychelles | 0（0,0） | 0.0（0.0,0.0） | 0（0,0） | 0.0（0.0,0.0） | 1.38(1.03,1.74) |
| Sierra Leone | 71（29,155） | 0.99（0.39,2.17） | 94（34,242） | 0.72（0.26,1.84） | -0.85(-1.15,-0.56) |
| Singapore | 69（49,89） | 3.43（2.43,4.39） | 92（55,126） | 3.19（1.94,4.38） | 0.18(-0.31,0.68) |
| Slovakia | 6（2,15） | 0.16（0.06,0.36） | 13（5,28） | 0.46（0.19,1.0） | 4.49(3.41,5.59) |
| Slovenia | 3（2,5） | 0.28（0.2,0.42） | 8（3,16） | 0.81（0.36,1.55） | 4.77(3.79,5.77) |
| Solomon Islands | 0（0,2） | 0.12（0.03,0.4） | 1（0,4） | 0.15（0.03,0.53） | 0.82(0.17,1.47) |
| Somalia | 321（132,686） | 2.16（0.89,4.64） | 526（157,1276） | 1.32（0.39,3.2） | -1.02(-1.48,-0.55) |
| South Africa | 125（53,248） | 0.26（0.11,0.51） | 260（83,536） | 0.52（0.16,1.07） | 2.73(1.49,3.99) |
| South Sudan | 186（80,362） | 1.89（0.81,3.74） | 195（83,382） | 1.3（0.54,2.57） | -0.51(-0.76,-0.25) |
| Spain | 370（278,492） | 1.67（1.25,2.22） | 458（264,680） | 2.33（1.36,3.51） | 1.39(0.53,2.26) |
| Sri Lanka | 39（16,85） | 0.22（0.09,0.47） | 62（26,134） | 0.38（0.17,0.84） | 3.33(2.72,3.94) |
| Sudan | 4（1,14） | 0.01（0.0,0.04） | 9（2,35） | 0.02（0.0,0.06） | 1.56(1.21,1.92) |
| Suriname | 0（0,0） | 0.0（0.0,0.0） | 0（0,0） | 0.0（0.0,0.0） | 1.94(1.28,2.60) |
| Sweden | 2（1,3） | 0.04（0.03,0.07） | 3（1,5） | 0.05（0.03,0.1） | 3.34(-0.66,7.50) |
| Switzerland | 86（60,122） | 2.17（1.52,3.09） | 145（85,200） | 3.24（1.9,4.49） | 0.98(0.64,1.32) |
| Syrian Arab Republic | 0（0,2） | 0.0（0.0,0.01） | 0（0,1） | 0.01（0.0,0.01） | 2.15(1.54,2.76) |
| Taiwan | 45（33,61） | 0.28（0.21,0.38） | 105（60,181） | 1.17（0.67,1.99） | 6.66(5.53,7.81) |
| Tajikistan | 9（1,23） | 0.1（0.02,0.26） | 28（4,80） | 0.21（0.04,0.61） | 2.98(2.71,3.24) |
| Thailand | 240（116,425） | 0.45（0.22,0.8） | 128（58,280） | 0.44（0.2,0.96） | 0.18(-0.46,0.82) |
| Timor-Leste | 1(0,4) | 0.13（0.03,0.32） | 2（0,6） | 0.15（0.04,0.39） | 0.65(0.27,1.03) |
| Togo | 58（23,138） | 0.88（0.36,2.09） | 83（29,223） | 0.71（0.25,1.91） | -0.72(-0.95,-0.48) |
| Tokelau | 0(0,0) | 0.09（0.02,0.3） | 0（0,0） | 10.93（2.32,35.38） | 6.61(2.87,10.49) |
| Tonga | 0（0,0） | 0.15（0.03,0.48） | 0（0,1） | 0.29（0.07,1.04） | 2.13(1.75,2.50) |
| Trinidad and Tobago | 6（2,14） | 0.47（0.19,1.06） | 7（2,14） | 0.87（0.34,1.81） | 3.47(2.13,4.83) |
| Tunisia | 82（41,168） | 0.78（0.39,1.58） | 93（45,171） | 1.03（0.5,1.89） | 0.93(0.65,1.22) |
| Türkiye | 174（78,334） | 0.25（0.11,0.48） | 414（234,736） | 0.72（0.4,1.3） | 3.84(3.27,4.41) |
| Turkmenistan | 8（2,22） | 0.16（0.05,0.4） | 11（2,35） | 0.22（0.05,0.67） | 1.43(1.32,1.53) |
| Tuvalu | 0（0,0） | 0.16（0.04,0.49） | 0（0,0） | 0.19（0.04,0.63） | 0.95(0.58,1.32) |
| Uganda | 651（292,1237） | 1.9（0.85,3.64） | 1144（467,2715） | 1.58（0.65,3.76） | -0.38(-0.71,-0.04) |
| Ukraine | 135（64,268） | 0.35（0.17,0.7） | 85（43,152） | 0.47（0.23,0.85） | 1.63(1.18,2.09) |
| United Arab Emirates | 2（0,5） | 0.1（0.03,0.25） | 8（3,18） | 0.2（0.08,0.41） | 3.96(3.17,4.76) |
| United Kingdom | 69（58,82） | 0.18（0.15,0.22） | 188（151,229） | 0.5（0.4,0.61） | 1.68(0.38,3.00) |
| United Republic of Tanzania | 1043（427,2190） | 2.25（0.9,4.73） | 1747（657,3929） | 1.99（0.75,4.49） | 0.11(-0.15,0.37) |
| United States of America | 3726（3093,4405） | 1.91（1.59,2.26） | 1943（1330,2786） | 1.02（0.69,1.45） | -1.26(-1.75,-0.76) |
| United States Virgin Islands | 0（0,1） | 0.75（0.23,1.77） | 0（0,0） | 0.52（0.16,1.18） | -1.43(-1.92,-0.93) |
| Uruguay | 39（19,70） | 1.44（0.72,2.57） | 28（15,47） | 1.37（0.76,2.26） | -0.36(-0.69,-0.04) |
| Uzbekistan | 64（15,200） | 0.2（0.05,0.62） | 166（51,373） | 0.45（0.14,1.0） | 3.22(2.71,3.73) |
| Vanuatu | 0（0,0） | 0.09（0.02,0.3） | 0（0,1） | 0.1（0.02,0.33） | 1.14(0.47,1.82) |
| Venezuela (Bolivarian Republic of) | 112（49,215） | 0.45（0.2,0.87） | 129（60,282） | 0.59（0.28,1.29） | 1.62(1.07,2.17) |
| Vietnam | 938（417,1885） | 1.0（0.44,2.01） | 1370（574,2792） | 1.65（0.69,3.34） | 1.55(1.38,1.73) |
| Yemen | 2（0,7） | 0.01（0.0,0.03） | 5（0,21） | 0.01（0.0,0.04） | 1.85(1.48,2.22) |
| Zambia | 344（166,677） | 2.38（1.13,4.79） | 431（186,918） | 1.48（0.64,3.15） | -1.47(-1.68,-1.27) |
| Zimbabwe | 62（29,120） | 0.36（0.17,0.69） | 127（44,295） | 0.58（0.2,1.34） | 4.09(2.58,5.62) |

**Abbreviations**: ASR, age-standardized rate; RB, Retinoblastoma; EAPC, estimated annual percentage change; UIs, uncertainty intervals; CI, conﬁdence interval.

**Table S6** The number of DALYs and ASR-DALY for RB by country in 1990 and 2021, along with its EAPC

| **Location** | **1990** | | **2021** | | **EAPC**  **(95% CI) 1990-2021** |
| --- | --- | --- | --- | --- | --- |
|  | **Number (95% UIs)** | **ASR (95% UIs)** | **Number (95% UIs)** | **ASR (95% UIs)** |  |
| Afghanistan | 14（2,48） | 0.08（0.02,0.3） | 45（7,166） | 0.09（0.01,0.31） | -0.01(-0.22,0.21) |
| Albania | 0（0,1） | 0.01（0.01,0.03） | 0（0,0） | 0.02（0.0,0.05） | 0.31(-0.33,0.96) |
| Algeria | 701（359,1321） | 1.87（0.96,3.52） | 333（160,620） | 0.71（0.34,1.32） | -3.33(-3.61,-3.05) |
| American Samoa | 0（0,0） | 0.24（0.07,0.66） | 0（0,0） | 0.28（0.07,0.86） | 0.11(-0.17,0.39) |
| Andorra | 0（0,0） | 0.1（0.04,0.2） | 0（0,0） | 0.04（0.01,0.08） | -2.58(-2.85,-2.31) |
| Angola | 957（454,2040） | 5.24（2.48,10.99） | 1699（631,3863） | 3.06（1.14,6.95） | -1.55(-1.73,-1.36) |
| Antigua and Barbuda | 0（0,0） | 0.21（0.08,0.54） | 0（0,0） | 0.01（0.0,0.02） | -9.57(-11.23,-7.88) |
| Argentina | 530（311,893） | 1.53（0.9,2.58） | 96（54,151） | 0.3（0.17,0.47） | -4.18(-4.93,-3.42) |
| Armenia | 1（0,2） | 0.03（0.01,0.08） | 11（4,24） | 0.57（0.22,1.23） | 14.29(11.15,17.52) |
| Australia | 21（16,27） | 0.17（0.13,0.21） | 3（1,6） | 0.02（0.01,0.04） | -3.76(-5.15,-2.36) |
| Austria | 24（17,33） | 0.53（0.39,0.73） | 9（5,16） | 0.22（0.12,0.37） | -1.73(-2.72,-0.73) |
| Azerbaijan | 164（45,503） | 1.86（0.51,5.68） | 123（27,350） | 1.58（0.35,4.52） | -0.49(-0.87,-0.11) |
| Bahamas | 5（2,10） | 2.05（1.05,4.06） | 1（0,3） | 0.65（0.24,1.58） | -2.75(-4.24,-1.23) |
| Bahrain | 0（0,1） | 0.14（0.07,0.29） | 1（0,4） | 0.15（0.03,0.4） | 2.77(1.65,3.90) |
| Bangladesh | 12044（5543,23971） | 6.51（3.0,13.05） | 4768（2369,9633） | 3.24（1.61,6.55） | -2.33(-2.52,-2.14) |
| Barbados | 5（1,11） | 2.52（0.95,5.92） | 3（1,8） | 2.7（0.99,5.77） | 1.88(0.58,3.20) |
| Belarus | 96（48,187） | 1.19（0.59,2.31） | 65（18,174） | 1.3（0.36,3.47） | -0.30(-1.50,0.92) |
| Belgium | 28（18,40） | 0.47（0.31,0.67） | 16（9,25） | 0.27（0.16,0.41） | -1.62(-2.62,-0.60) |
| Belize | 5（2,10） | 1.79（0.84,3.6） | 0（0,1） | 0.15（0.07,0.32） | -7.39(-9.28,-5.45) |
| Benin | 1068（423,2345） | 11.23（4.42,24.75） | 1688（647,4279） | 7.38（2.83,18.66） | -1.25(-1.45,-1.05) |
| Bermuda | 0（0,0） | 0.0（0.0,0.0） | 0（0,0） | 0.0（0.0,0.0） | 3.22(-0.15,6.70) |
| Bhutan | 51（15,129） | 5.46（1.59,14.0） | 24（11,49） | 4.05（1.87,8.17） | -0.93(-1.22,-0.63) |
| Bolivia (Plurinational State of) | 1108（494,2426） | 11.29（4.97,24.65） | 795（339,1645） | 6.66（2.84,13.8） | -1.79(-1.91,-1.67) |
| Bosnia and Herzegovina | 4（1,10） | 0.12（0.03,0.29） | 0（0,1） | 0.04（0.01,0.11） | -2.79(-3.44,-2.13) |
| Botswana | 54（22,103） | 2.59（1.05,4.91） | 67（26,143） | 2.87（1.14,6.07） | 0.78(0.52,1.04) |
| Brazil | 5309（4136,6727） | 3.12（2.44,3.95） | 1559（1057,2112） | 0.94（0.64,1.27） | -3.15(-3.63,-2.67) |
| Brunei Darussalam | 21（7,48） | 6.53（2.27,14.36） | 14（7,28） | 4.7（2.26,9.09） | -0.27(-0.56,0.03) |
| Bulgaria | 19（7,38） | 0.35（0.13,0.7） | 5（1,14） | 0.18（0.05,0.46） | -2.97(-4.06,-1.86) |
| Burkina Faso | 2033（865,4763） | 11.31（4.8,26.61） | 2938（1250,6752） | 7.39（3.14,17.12） | -1.11(-1.54,-0.69) |
| Burundi | 2157（1027,3993） | 20.75（9.88,38.8） | 2290（973,5020） | 10.75（4.53,23.58） | -1.04(-1.57,-0.51) |
| Côte d'Ivoire | 575（244,1414） | 4.14（1.51,9.44） | 795（311,1910） | 4.19（1.85,9.45） | 0.14(-0.37,0.65) |
| Cabo Verde | 24（8,55） | 1.39（0.37,3.1） | 18（8,42） | 0.94（0.22,2.28） | -1.48(-1.61,-1.35) |
| Cambodia | 241（64,544） | 8.77（3.52,19.87） | 165（38,398） | 5.38（1.93,12.18） | -0.94(-1.22,-0.65) |
| Cameroon | 1686（683,3804） | 0.49（0.34,0.66） | 2588（930,5866） | 0.15（0.09,0.26） | -2.89(-3.30,-2.48) |
| Canada | 94（67,127） | 5.68（2.64,11.57） | 30（17,53） | 5.3（2.08,12.16） | -0.03(-0.21,0.16) |
| Central African Republic | 270（126,547） | 9.78（3.97,22.2） | 434（170,998） | 7.88（3.36,17.5） | -0.60(-0.69,-0.52) |
| Chad | 1139（462,2579） | 0.72（0.46,1.06） | 2746（1183,6073） | 0.3（0.15,0.52） | -3.73(-4.71,-2.73) |
| Chile | 101（64,150） | 2.49（1.29,3.85） | 34（17,60） | 0.87（0.36,1.32） | -2.25(-2.74,-1.75) |
| China | 27716（14354,42866） | 2.92（2.04,4.13） | 6765（2802,10311） | 1.37（0.79,2.24） | -0.04(-1.02,0.95) |
| Colombia | 1196（837,1688） | 19.56（8.87,41.66） | 482（279,788） | 16.55（6.59,33.55） | -0.63(-0.89,-0.38) |
| Comoros | 157（72,332） | 4.61（1.86,10.21） | 134（53,271） | 2.66（0.89,6.46） | -1.49(-1.72,-1.26) |
| Congo | 176（71,389） | 0.79（0.19,2.68） | 170（57,415） | 2.9（0.73,9.43） | 0.29(-1.16,1.77) |
| Cook Islands | 0（0,0） | 2.93（1.83,4.63） | 0（0,1） | 0.84（0.48,1.48） | -2.77(-3.45,-2.08) |
| Costa Rica | 117（73,186） | 2.57（1.08,6.34） | 27（15,47） | 1.86（0.73,4.46） | -2.29(-4.57,0.04) |
| Croatia | 2（1,3） | 0.07（0.05,0.11） | 0（0,1） | 0.03（0.01,0.07） | -9.11(-11.14,-7.04) |
| Cuba | 143（83,227） | 1.63（0.95,2.58） | 1（0,3） | 0.03（0.02,0.05） | 3.06(2.45,3.68) |
| Cyprus | 0（0,0） | 0.01（0.01,0.03） | 0（0,0） | 0.02（0.01,0.05） | -1.82(-2.83,-0.81) |
| Czechia | 24（16,34） | 0.36（0.24,0.51） | 10（4,20） | 0.18（0.09,0.35） | -0.62(-0.96,-0.28) |
| Democratic People's Republic of Korea | 408（199,832） | 1.77（0.87,3.6） | 241（101,524） | 1.59（0.67,3.45） | -0.26(-0.41,-0.12) |
| Democratic Republic of the Congo | 2967（1268,5616） | 4.29（1.81,8.18） | 3351（1287,7503） | 2.49（0.96,5.58） | -1.13(-1.44,-0.81) |
| Denmark | 28（21,37） | 1.01（0.76,1.31） | 15（8,24） | 0.51（0.29,0.77） | -2.20(-3.20,-1.18) |
| Djibouti | 139（61,252） | 22.05（9.67,40.32） | 197（75,418） | 13.65（5.22,28.84） | -1.01(-1.48,-0.53) |
| Dominica | 0（0,0） | 0.0（0.0,0.01） | 0（0,0） | 0.0（0.0,0.01） | 0.19(-0.23,0.62) |
| Dominican Republic | 115（40,226） | 1.19（0.42,2.34） | 50（17,113） | 0.49（0.17,1.11） | -1.01(-1.84,-0.18) |
| Ecuador | 619（362,997） | 4.68（2.72,7.55） | 632（343,1101） | 3.73（2.02,6.53） | 0.31(-0.11,0.74) |
| Egypt | 360（122,921） | 0.44（0.15,1.12） | 276（91,869） | 0.21（0.07,0.67） | -1.60(-3.19,0.01) |
| El Salvador | 99（42,231） | 1.33（0.57,3.11） | 33（15,72） | 0.55（0.25,1.18） | -2.79(-2.84,-2.73) |
| Equatorial Guinea | 38（16,88） | 4.96（2.12,11.53） | 30（7,84） | 1.62（0.37,4.42） | -4.27(-4.59,-3.95) |
| Eritrea | 1318（485,2756） | 21.89（8.03,46.08） | 1756（617,3645） | 19.34（6.82,40.11） | -0.56(-0.82,-0.30) |
| Estonia | 8（4,15） | 0.72（0.34,1.25） | 1（0,2） | 0.19（0.1,0.32） | -2.81(-4.23,-1.37) |
| Eswatini | 40（15,76） | 2.83（1.11,5.36） | 42（16,81） | 3.0（1.16,5.81） | 0.66(0.39,0.93) |
| Ethiopia | 11648（5204,21754） | 12.53（5.6,23.44） | 10082（5311,16962） | 6.41（3.37,10.79） | -2.40(-2.79,-2.01) |
| Fiji | 9（1,29） | 1.04（0.18,3.05） | 11（1,31） | 1.24（0.19,3.43） | 0.81(0.31,1.32) |
| Finland | 21（15,29） | 0.68（0.48,0.91） | 18（11,27） | 0.69（0.42,1.04） | -0.25(-0.92,0.42) |
| France | 284（224,355） | 0.72（0.57,0.91） | 147（87,226） | 0.4（0.24,0.62） | -1.35(-2.32,-0.36) |
| Gabon | 44（16,99） | 2.96（1.09,6.58） | 43（13,109） | 2.03（0.61,5.07） | -0.68(-0.97,-0.39) |
| Gambia | 164（71,346） | 9.31（4.02,19.74） | 296（114,668） | 8.43（3.24,19.06） | -0.36(-0.74,0.03) |
| Georgia | 9（1,33） | 0.21（0.04,0.7） | 38（10,101） | 1.53（0.4,4.07） | 9.99(8.61,11.39) |
| Germany | 206（138,308） | 0.46（0.31,0.69） | 76（44,120） | 0.19（0.11,0.3） | -1.98(-2.39,-1.56) |
| Ghana | 1837（706,4434） | 7.2（2.74,17.47） | 2041（703,4902） | 4.43（1.53,10.62） | -1.13(-1.43,-0.82) |
| Greece | 4（3,7） | 0.08（0.05,0.12） | 4（2,7） | 0.09（0.05,0.17） | 1.54(0.04,3.06) |
| Greenland | 0（0,0） | 0.74（0.16,1.54） | 0（0,0） | 0.15（0.04,0.34） | -3.24(-4.34,-2.13) |
| Grenada | 0（0,0） | 0.17（0.08,0.33） | 0（0,0） | 0.01（0.01,0.02） | -7.58(-9.53,-5.59) |
| Guam | 0（0,2） | 0.49（0.13,1.5） | 0（0,2） | 0.48（0.12,1.65） | 1.97(1.41,2.54) |
| Guatemala | 568（238,1161） | 3.81（1.6,7.78） | 295（158,499） | 1.84（0.99,3.12） | -1.80(-1.97,-1.64) |
| Guinea | 2201（1042,4612） | 19.96（9.41,41.85） | 2674（1054,6113） | 12.08（4.77,27.69） | -1.23(-1.46,-1.00) |
| Guinea-Bissau | 237(98,569) | 13.19（5.43,31.81） | 221（85,527） | 6.75（2.62,16.16） | -1.44(-1.90,-0.98) |
| Guyana | 2（1,4） | 0.22（0.11,0.43） | 6（3,12） | 0.86（0.43,1.65） | 3.25(-0.08,6.70) |
| Haiti | 72（20,157） | 0.71（0.2,1.54） | 63（19,152） | 0.41（0.12,0.99） | -1.59(-1.74,-1.44) |
| Honduras | 276（135,590） | 3.45（1.69,7.36） | 245（115,517） | 2.23（1.05,4.71） | -1.48(-1.59,-1.36) |
| Hungary | 29（19,44） | 0.47（0.31,0.71） | 12（5,24） | 0.28（0.12,0.53） | -0.37(-1.75,1.02) |
| Iceland | 1（0,1） | 0.55（0.39,0.73） | 1（0,2） | 0.69（0.37,1.22） | 1.14(-0.11,2.42) |
| India | 59516（27178,90412） | 5.12（2.34,7.78） | 34814（20462,53353） | 3.08（1.8,4.72） | -1.73(-1.97,-1.50) |
| Indonesia | 2105（535,3694） | 0.93（0.24,1.64） | 1605（382,2951） | 0.72（0.17,1.33） | -0.65(-0.85,-0.45) |
| Iran (Islamic Republic of) | 38（5,93） | 0.04（0.01,0.11） | 18（4,36） | 0.03（0.01,0.05） | 1.26(0.52,2.01) |
| Iraq | 485（241,941） | 1.6（0.8,3.11） | 665（332,1253） | 1.51（0.76,2.84） | -0.43(-0.66,-0.20) |
| Ireland | 10（7,14） | 0.36（0.26,0.48） | 3（1,6） | 0.11（0.06,0.21） | -3.50(-3.77,-3.23) |
| Israel | 15（9,23） | 0.29（0.18,0.45） | 19（12,30） | 0.21（0.13,0.33） | -0.26(-1.02,0.51) |
| Italy | 119（102,137） | 0.42（0.37,0.49） | 88（55,129） | 0.39（0.24,0.57） | -1.38(-2.95,0.22) |
| Jamaica | 49（18,116） | 1.77（0.66,4.16） | 7（2,19） | 0.43（0.15,1.08） | -3.97(-5.34,-2.58) |
| Japan | 299（261,345） | 0.43（0.37,0.49） | 81（57,114） | 0.17（0.12,0.24） | -1.58(-2.88,-0.27) |
| Jordan | 67（34,121） | 1.14（0.59,2.06） | 63（33,111） | 0.56（0.29,0.99） | -2.96(-3.39,-2.52) |
| Kazakhstan | 529（143,1360） | 2.86（0.77,7.36） | 189（50,472） | 0.99（0.26,2.46） | -3.33(-4.21,-2.44) |
| Kenya | 19722（10431,29723） | 46.98（24.96,70.78） | 18846（10691,29759） | 31.4（17.84,49.56） | -0.40(-0.78,-0.02) |
| Kiribati | 1（0,5） | 1.51（0.37,4.95） | 1（0,5） | 1.31（0.35,4.12） | -1.07(-1.42,-0.71) |
| Kuwait | 23（15,34） | 1.16（0.79,1.69） | 1（0,1） | 0.04（0.02,0.06） | -10.74(-14.78,-6.50) |
| Kyrgyzstan | 179（52,455） | 2.85（0.83,7.22） | 146（50,337） | 1.83（0.63,4.2） | 2.12(-0.22,4.52) |
| Lao People's Democratic Republic | 101（24,249） | 1.5（0.37,3.71） | 80（17,205） | 0.99（0.22,2.51） | -1.24(-1.42,-1.06) |
| Latvia | 18（8,34） | 0.91（0.44,1.71） | 0（0,0） | 0.05（0.02,0.1） | -9.26(-9.60,-8.92) |
| Lebanon | 24（9,49） | 0.63（0.25,1.3） | 16（7,32） | 0.41（0.18,0.78） | -1.26(-1.48,-1.03) |
| Lesotho | 72（28,139） | 2.97（1.17,5.75） | 76（30,158） | 3.68（1.47,7.62） | 1.14(0.89,1.39) |
| Liberia | 383（154,901） | 8.62（3.46,20.48） | 343（126,880） | 4.52（1.67,11.6） | -1.94(-2.66,-1.22) |
| Libya | 135（65,293） | 2.14（1.03,4.62） | 56（26,114） | 1.27（0.59,2.57） | -1.86(-2.05,-1.68) |
| Lithuania | 22（11,41） | 0.77（0.4,1.42） | 0（0,0） | 0.03（0.01,0.06） | -8.99(-10.33,-7.63) |
| Luxembourg | 0（0,0） | 0.04（0.02,0.07） | 0（0,0） | 0.02（0.0,0.05） | -1.44(-3.06,0.20) |
| Madagascar | 3925（1693,7946） | 18.69（7.9,38.25） | 5439（2087,11991） | 13.33（5.11,29.42） | -0.98(-1.13,-0.83) |
| Malawi | 8770（4352,16404） | 47.89（23.82,91.91） | 9484（3905,20919） | 34.9（14.34,77.09） | -0.62(-0.93,-0.31) |
| Malaysia | 506（180,1131） | 2.16（0.77,4.82） | 277（104,595） | 1.11（0.42,2.38） | -1.84(-2.35,-1.33) |
| Maldives | 1（0,2） | 0.25（0.11,0.58） | 0（0,0） | 0.13（0.06,0.27） | -2.08(-2.26,-1.90) |
| Mali | 3498（1556,7338） | 21.57（9.36,45.18） | 4489（1575,12921） | 10.14（3.54,29.27） | -2.17(-2.35,-2.00) |
| Malta | 0（0,0） | 0.05（0.03,0.06） | 0（0,0） | 0.02（0.01,0.04） | -1.98(-3.02,-0.94) |
| Marshall Islands | 0（0,1） | 0.85（0.2,2.63） | 0（0,2） | 1.12（0.25,3.63） | 0.79(0.44,1.14) |
| Mauritania | 293（119,717） | 8.24（3.35,20.22） | 272（112,607） | 4.16（1.72,9.26） | -2.46(-2.64,-2.27) |
| Mauritius | 1（1,3） | 0.18（0.1,0.33） | 1（0,2） | 0.24（0.14,0.43） | 4.98(1.67,8.40) |
| Mexico | 4704（3937,5580） | 4.05（3.39,4.81） | 1770（1147,2595） | 1.73（1.12,2.54） | -2.90(-3.35,-2.45) |
| Micronesia (Federated States of) | 1（0,6） | 1.23（0.3,3.93） | 0（0,3） | 0.94（0.21,3.3） | -0.35(-0.65,-0.05) |
| Monaco | 0（0,0） | 0.03（0.01,0.05） | 0（0,0） | 0.07（0.03,0.14） | 0.11(-0.89,1.11) |
| Mongolia | 67（12,175） | 2.07（0.38,5.34） | 86（29,217） | 2.19（0.75,5.51） | 0.62(0.30,0.93) |
| Montenegro | 0（0,0） | 0.06（0.02,0.16） | 0（0,0） | 0.02（0.01,0.04） | -2.16(-2.87,-1.44) |
| Morocco | 3100（1402,6124） | 8.85（3.99,17.54） | 1676（716,3249） | 5.14（2.2,9.91） | -1.36(-1.60,-1.12) |
| Mozambique | 7035（3416,14047） | 30.23（14.6,60.72） | 9565（4511,18287） | 18.6（8.78,35.59） | -1.10(-1.38,-0.82) |
| Myanmar | 842（201,2258） | 1.68（0.4,4.51） | 621（140,1752） | 1.18（0.27,3.35） | -1.33(-1.48,-1.18) |
| Namibia | 185（85,402） | 8.42（3.85,18.19） | 294（139,611） | 10.58（5.03,21.98） | 1.31(0.89,1.72) |
| Nauru | 0（0,0） | 1.37（0.35,4.05） | 0（0,0） | 1.38（0.34,4.55） | 0.04(-0.28,0.36) |
| Nepal | 1920（876,4332） | 6.04（2.75,13.71） | 1052（503,2151） | 3.39（1.62,6.94） | -1.50(-1.74,-1.25) |
| Netherlands | 58（46,75） | 0.63（0.5,0.81） | 35（21,51） | 0.4（0.25,0.59） | -1.50(-2.22,-0.77) |
| New Zealand | 2（1,4） | 0.09（0.04,0.16） | 0（0,1） | 0.02（0.01,0.05） | -2.69(-6.29,1.05) |
| Nicaragua | 393（151,1069） | 5.99（2.31,16.3） | 123（59,232） | 1.87（0.89,3.52） | -2.93(-3.37,-2.48) |
| Niger | 2109（900,5343） | 13.4（5.66,34.41） | 3396（1337,8357） | 6.91（2.72,17.02） | -2.13(-2.41,-1.86) |
| Nigeria | 12857（5841,20276） | 8.61（3.91,13.65） | 24939（8828,43745） | 6.85（2.42,12.01） | -0.70(-0.83,-0.58) |
| Niue | 0（0,0） | 1.54（0.36,4.94） | 0（0,0） | 5.4（1.38,18.5） | 0.82(-0.37,2.03) |
| North Macedonia | 0（0,0） | 0.01（0.0,0.02） | 0（0,0） | 0.0（0.0,0.01） | -0.94(-1.71,-0.17) |
| Northern Mariana Islands | 0（0,0） | 0.16（0.04,0.57） | 0（0,0） | 0.09（0.02,0.28） | -1.23(-1.75,-0.71) |
| Norway | 2（2,3） | 0.11（0.09,0.12） | 0（0,0） | 0.01（0.0,0.01） | -7.35(-9.23,-5.43) |
| Oman | 26（9,53） | 0.82（0.29,1.7） | 12（4,24） | 0.29（0.11,0.58） | -2.56(-3.09,-2.01) |
| Pakistan | 8223（3771,15545） | 4.49（2.05,8.55） | 18533（8336,33047） | 6.25（2.81,11.14） | 1.67(1.42,1.91) |
| Palau | 0（0,0） | 0.79（0.21,2.56） | 0（0,0） | 0.76（0.18,2.85） | -0.03(-0.26,0.20) |
| Palestine | 10（3,23） | 0.29（0.1,0.62） | 11（4,20） | 0.18（0.08,0.34） | -0.71(-1.35,-0.07) |
| Panama | 85（38,179） | 2.98（1.35,6.28） | 60（31,111） | 1.59（0.84,2.89） | -1.50(-1.72,-1.28) |
| Papua New Guinea | 76（16,264） | 1.23（0.26,4.23） | 200（43,756） | 1.37（0.3,5.16） | 0.35(-0.07,0.77) |
| Paraguay | 224（85,442） | 3.69（1.4,7.25） | 167（65,383） | 2.52（0.97,5.82） | -0.50(-0.76,-0.24) |
| Peru | 2470（1262,4608） | 8.58（4.36,16.03） | 881（377,1812） | 2.68（1.14,5.5） | -3.04(-3.26,-2.81) |
| Philippines | 2219（561,4000） | 2.47（0.62,4.44） | 3007（868,5084） | 2.66（0.77,4.49） | 0.78(0.49,1.06) |
| Poland | 17（13,21） | 0.06（0.04,0.07） | 5（2,8） | 0.03（0.02,0.05） | -3.97(-6.67,-1.18) |
| Portugal | 143（104,201） | 2.32（1.72,3.25） | 43（25,68） | 1.01（0.59,1.59） | -2.42(-4.00,-0.82) |
| Puerto Rico | 18（9,33） | 0.56（0.31,1.06） | 0（0,1） | 0.05（0.03,0.09） | -7.01(-9.23,-4.74) |
| Qatar | 0（0,0） | 0.0（0.0,0.01） | 0（0,0） | 0.0（0.0,0.0） | -4.60(-7.41,-1.72) |
| Republic of Korea | 425（225,718） | 1.2（0.64,2.0） | 82（45,139） | 0.5（0.28,0.84） | -2.68(-3.07,-2.28) |
| Republic of Moldova | 152（59,346） | 3.51（1.37,7.93） | 9（4,18） | 0.58（0.25,1.17） | -6.21(-6.64,-5.78) |
| Romania | 586（202,1310） | 3.28（1.13,7.34） | 63（31,122） | 0.66（0.32,1.29） | -6.15(-6.88,-5.42) |
| Russian Federation | 1597（1149,2161） | 1.34（0.97,1.82） | 248（189,326） | 0.31（0.23,0.4） | -6.08(-7.09,-5.06) |
| Rwanda | 3213（1527,6656） | 24.48（11.68,50.72） | 2114（842,4372） | 12.18（4.84,25.16） | -1.98(-2.35,-1.62) |
| Saint Kitts and Nevis | 0（0,0） | 0.01（0.0,0.01） | 0（0,0） | 0.0（0.0,0.0） | -8.71(-10.34,-7.04) |
| Saint Lucia | 0（0,0） | 0.13（0.05,0.29） | 0（0,0） | 0.03（0.01,0.06） | -4.66(-5.89,-3.40) |
| Saint Vincent and the Grenadines | 12（6,29） | 9.87（4.54,22.41） | 0（0,1） | 0.62（0.24,1.33） | -8.17(-9.78,-6.53) |
| Samoa | 5（2,12） | 2.21（0.83,4.91） | 4（1,12） | 1.57（0.53,4.21） | -1.07(-1.27,-0.87) |
| San Marino | 0（0,0） | 0.11（0.04,0.25） | 0（0,0） | 0.07（0.02,0.15） | -1.40(-1.67,-1.12) |
| Sao Tome and Principe | 18（6,46） | 9.44（3.33,23.19） | 12（4,28） | 4.84（1.74,11.3） | -1.75(-2.80,-0.68) |
| Saudi Arabia | 355（36,850） | 1.48（0.15,3.55） | 92（19,221） | 0.37（0.08,0.88） | -3.65(-5.25,-2.03) |
| Senegal | 1599（649,3864） | 11.39（4.54,27.72） | 1533（617,3628） | 6.83（2.75,16.16） | -1.03(-1.46,-0.60) |
| Serbia | 43（17,91） | 0.62（0.25,1.29） | 4（1,10） | 0.13（0.05,0.27） | -5.50(-6.18,-4.81) |
| Seychelles | 0（0,0） | 0.0（0.0,0.0） | 0（0,0） | 0.0（0.0,0.0） | -1.48(-1.70,-1.27) |
| Sierra Leone | 695（280,1495） | 9.56（3.74,20.8） | 795（292,1919） | 6.06（2.25,14.61） | -1.18(-1.44,-0.91) |
| Singapore | 53（39,69） | 2.63（1.92,3.5） | 19（10,30） | 0.68（0.38,1.05） | -3.91(-4.53,-3.28) |
| Slovakia | 12（5,28） | 0.3（0.12,0.66） | 8（3,18） | 0.3（0.14,0.65） | 0.95(-0.02,1.92) |
| Slovenia | 3（2,5） | 0.3（0.22,0.43） | 2（1,5） | 0.27（0.13,0.52） | 0.34(-0.47,1.15) |
| Solomon Islands | 6（1,21） | 1.15（0.24,3.84） | 12（2,40） | 1.3（0.3,4.34） | 0.51(-0.15,1.16) |
| Somalia | 3128（1323,6938） | 21.02（8.82,47.18） | 5091（1505,12332） | 12.72（3.8,30.86） | -1.04(-1.50,-0.57) |
| South Africa | 844（370,1540） | 1.75（0.77,3.2） | 1030（335,1960） | 2.04（0.67,3.9） | 0.76(-0.03,1.56) |
| South Sudan | 1803（777,3564） | 18.29（7.81,36.87） | 1750（772,3432） | 11.63（5.07,23.06） | -0.72(-0.98,-0.47) |
| Spain | 148（114,193） | 0.66（0.52,0.86） | 75（44,117） | 0.38（0.23,0.6） | -1.47(-2.56,-0.37) |
| Sri Lanka | 212（91,434） | 1.17（0.5,2.39） | 87（39,189） | 0.54（0.25,1.15） | -1.50(-2.06,-0.94) |
| Sudan | 40（9,134） | 0.12（0.03,0.4） | 38（8,134） | 0.07（0.02,0.24） | -1.46(-1.71,-1.21) |
| Suriname | 0（0,0） | 0.01（0.0,0.02） | 0（0,0） | 0.01（0.0,0.02） | -0.17(-0.76,0.42) |
| Sweden | 0（0,1） | 0.01（0.01,0.02） | 0（0,0） | 0.01（0.0,0.02） | 1.38(-2.37,5.28) |
| Switzerland | 24（17,33） | 0.61（0.44,0.84） | 23（14,35） | 0.53（0.31,0.8） | -0.75(-1.28,-0.21) |
| Syrian Arab Republic | 5（2,12） | 0.03（0.01,0.06） | 1（0,2） | 0.01（0.0,0.02） | -2.36(-2.63,-2.09) |
| Taiwan | 69（53,89） | 0.42（0.32,0.54） | 54（32,89） | 0.6（0.36,0.98） | 2.96(2.03,3.91) |
| Tajikistan | 63（11,145） | 0.72（0.13,1.66） | 133（25,403） | 1.03（0.19,3.1） | 1.71(1.35,2.08) |
| Thailand | 1071（549,1863） | 1.99（1.03,3.4） | 156（69,354） | 0.53（0.23,1.2） | -4.15(-4.67,-3.63) |
| Timor-Leste | 16(4,38) | 1.26（0.32,2.93） | 18（4,46） | 1.04（0.25,2.55） | -0.66(-0.91,-0.42) |
| Togo | 560（232,1360） | 8.43（3.5,20.55） | 629（239,1570） | 5.38（2.05,13.42） | -1.37(-1.52,-1.21) |
| Tokelau | 0(0,0) | 0.8（0.19,2.55） | 0（0,1） | 32.9（6.75,108.87） | 3.21(-0.34,6.89) |
| Tonga | 1（0,4） | 0.82（0.18,2.71） | 1（0,4） | 0.96（0.23,3.17） | 0.55(0.13,0.96) |
| Trinidad and Tobago | 36（15,79） | 2.63（1.12,5.72） | 17（7,34） | 2.08（0.86,4.08） | 0.23(-1.07,1.54) |
| Tunisia | 303（159,582） | 2.84（1.49,5.46） | 106（52,189） | 1.16（0.58,2.09） | -2.89(-3.12,-2.67) |
| Türkiye | 1076（531,1984） | 1.55（0.77,2.86） | 437（259,765） | 0.76（0.45,1.32） | -1.16(-1.32,-1.01) |
| Turkmenistan | 54（16,132） | 0.97（0.3,2.38） | 35（8,100） | 0.67（0.16,1.88） | -0.96(-1.38,-0.55) |
| Tuvalu | 0（0,0） | 1.51（0.37,4.85） | 0（0,0） | 1.01（0.23,3.38） | -2.34(-2.69,-1.98) |
| Uganda | 6288（2788,11540） | 18.28（8.06,33.65） | 8989（3994,19338） | 12.44（5.52,26.9） | -0.93(-1.22,-0.63) |
| Ukraine | 266（131,509） | 0.69（0.34,1.31） | 113（57,196） | 0.61（0.29,1.06） | -0.16(-0.54,0.22) |
| United Arab Emirates | 11（3,28） | 0.52（0.17,1.26） | 14（5,29） | 0.32（0.13,0.67） | 0.17(-0.47,0.80) |
| United Kingdom | 29（26,33） | 0.08（0.07,0.09） | 56（44,71） | 0.15（0.12,0.19） | -0.07(-1.80,1.70) |
| United Republic of Tanzania | 10044（4174,21275） | 21.62（8.77,46.21） | 13365（5531,27551） | 15.25（6.3,31.49） | -0.52(-0.80,-0.24) |
| United States of America | 1367（1201,1563） | 0.7（0.62,0.8） | 470（348,635） | 0.24（0.18,0.33） | -2.58(-3.19,-1.97) |
| United States Virgin Islands | 2（0,6） | 2.6（0.9,6.02） | 0（0,0） | 0.86（0.28,2.0） | -3.59(-4.01,-3.17) |
| Uruguay | 54（31,89） | 1.98（1.16,3.28） | 17（9,29） | 0.82（0.44,1.41） | -2.94(-3.37,-2.52) |
| Uzbekistan | 325（79,973） | 1.01（0.25,3.03） | 497（164,1084） | 1.34（0.44,2.92） | 1.03(0.69,1.37) |
| Vanuatu | 2（0,7） | 0.83（0.18,2.85） | 3（0,11） | 0.8（0.17,2.73） | 0.77(0.07,1.48) |
| Venezuela (Bolivarian Republic of) | 650（318,1256） | 2.62（1.28,5.04） | 304（146,645） | 1.38（0.66,2.94） | -1.36(-1.67,-1.04) |
| Vietnam | 6165（3212,12184） | 6.57（3.42,12.97） | 2386（1028,4706） | 2.86（1.24,5.62） | -2.90(-3.05,-2.75) |
| Yemen | 22（5,75） | 0.08（0.02,0.27） | 31（5,110） | 0.07（0.01,0.24） | -0.48(-0.72,-0.24) |
| Zambia | 3336（1607,6569） | 22.99（10.81,45.97） | 3505（1566,7272） | 12.03（5.38,24.94） | -1.89(-2.18,-1.59) |
| Zimbabwe | 540（272,999） | 3.1（1.56,5.73） | 1160（396,2680） | 5.27（1.8,12.18） | 4.74(3.14,6.37) |

**Abbreviations**: ASR, age-standardized rate; DALYs, disability-adjusted life-years; RB, Retinoblastoma; EAPC, estimated annual percentage change; UIs, uncertainty intervals; CI, conﬁdence interval.

**Table S7** The number of deaths and ASDR for RB by country in 1990 and 2021, along with its EAPC

| **Location** | **1990** | | **2021** | | **EAPC (95% CI) 1990-2021** |
| --- | --- | --- | --- | --- | --- |
|  | **Number (95% UIs)** | **ASR (95% UIs)** | **Number (95% UIs)** | **ASR (95% UIs)** |  |
| Afghanistan | 0（0,0） | 0.0（0.0,0.0） | 0（0,1） | 0.0（0.0,0.0） | 0.01(-0.21,0.23) |
| Albania | 0（0,0） | 0.0（0.0,0.0） | 0（0,0） | 0.0（0.0,0.0） | 0.09(-0.57,0.74) |
| Algeria | 8（4,15） | 0.02（0.01,0.04） | 3（1,6） | 0.01（0.0,0.01） | -3.42(-3.70,-3.13) |
| American Samoa | 0（0,0） | 0.0（0.0,0.01） | 0（0,0） | 0.0（0.0,0.01） | 0.10(-0.18,0.37) |
| Andorra | 0（0,0） | 0.0（0.0,0.0） | 0（0,0） | 0.0（0.0,0.0） | -3.42(-3.62,-3.23) |
| Angola | 10（5,23） | 0.06（0.03,0.13） | 19（7,44） | 0.04（0.01,0.08） | -1.54(-1.72,-1.35) |
| Antigua and Barbuda | 0（0,0） | 0.0（0.0,0.01） | 0（0,0） | 0.0（0.0,0.0） | -9.62(-11.27,-7.94) |
| Argentina | 5（3,9） | 0.02（0.01,0.03） | 1（0,1） | 0.0（0.0,0.01） | -4.34(-5.08,-3.58) |
| Armenia | 0（0,0） | 0.0（0.0,0.0） | 0（0,0） | 0.01（0.0,0.01） | 14.17(11.04,17.39) |
| Australia | 0（0,0） | 0.0（0.0,0.0） | 0（0,0） | 0.0（0.0,0.0） | -4.68(-5.98,-3.37) |
| Austria | 0（0,0） | 0.01（0.0,0.01） | 0（0,0） | 0.0（0.0,0.0） | -2.63(-3.67,-1.57) |
| Azerbaijan | 1（0,5） | 0.02（0.01,0.07） | 1（0,4） | 0.02（0.0,0.05） | -0.56(-0.94,-0.17) |
| Bahamas | 0（0,0） | 0.02（0.01,0.05） | 0（0,0） | 0.01（0.0,0.02） | -2.81(-4.30,-1.30) |
| Bahrain | 0（0,0） | 0.0（0.0,0.0） | 0（0,0） | 0.0（0.0,0.0） | 2.58(1.48,3.70) |
| Bangladesh | 137（63,275） | 0.07（0.03,0.15） | 54（26,109） | 0.04（0.02,0.07） | -2.36(-2.54,-2.17) |
| Barbados | 0（0,0） | 0.03（0.01,0.07） | 0（0,0） | 0.03（0.01,0.06） | 1.81(0.51,3.13) |
| Belarus | 1（0,2） | 0.01（0.01,0.03） | 0（0,1） | 0.01（0.0,0.04） | -0.48(-1.66,0.72) |
| Belgium | 0（0,0） | 0.0（0.0,0.01） | 0（0,0） | 0.0（0.0,0.0） | -2.41(-3.43,-1.38) |
| Belize | 0（0,0） | 0.02（0.01,0.04） | 0（0,0） | 0.0（0.0,0.0） | -7.42(-9.31,-5.48) |
| Benin | 12（4,26） | 0.13（0.05,0.28） | 19（7,48） | 0.08（0.03,0.21） | -1.25(-1.45,-1.05) |
| Bermuda | 0（0,0） | 0.0（0.0,0.0） | 0（0,0） | 0.0（0.0,0.0） | 2.85(-0.52,6.34) |
| Bhutan | 0（0,1） | 0.06（0.02,0.16） | 0（0,0） | 0.05（0.02,0.09） | -0.95(-1.25,-0.65) |
| Bolivia (Plurinational State of) | 12（5,27） | 0.13（0.06,0.28） | 9（3,18） | 0.08（0.03,0.16） | -1.81(-1.93,-1.69) |
| Bosnia and Herzegovina | 0（0,0） | 0.0（0.0,0.0） | 0（0,0） | 0.0（0.0,0.0） | -2.98(-3.61,-2.34) |
| Botswana | 0（0,1） | 0.03（0.01,0.06） | 0（0,1） | 0.03（0.01,0.07） | 0.76(0.50,1.02) |
| Brazil | 60（47,77） | 0.04（0.03,0.05） | 17（12,24） | 0.01（0.01,0.01） | -3.18(-3.66,-2.70) |
| Brunei Darussalam | 0（0,0） | 0.07（0.03,0.16） | 0（0,0） | 0.05（0.02,0.1） | -0.30(-0.59,0.00) |
| Bulgaria | 0（0,0） | 0.0（0.0,0.01） | 0（0,0） | 0.0（0.0,0.01） | -3.04(-4.14,-1.92) |
| Burkina Faso | 23（9,54） | 0.13（0.05,0.3） | 33（14,77） | 0.08（0.04,0.2） | -1.11(-1.54,-0.69) |
| Burundi | 24（11,45） | 0.24（0.11,0.44） | 26（11,57） | 0.12（0.05,0.27） | -1.04(-1.57,-0.51) |
| Côte d'Ivoire | 6（2,16） | 0.05（0.02,0.11） | 9（3,21） | 0.05（0.02,0.1） | 0.08(-0.43,0.59) |
| Cabo Verde | 0（0,0） | 0.02（0.0,0.04） | 0（0,0） | 0.01（0.0,0.03） | -1.49(-1.62,-1.36) |
| Cambodia | 2（0,6） | 0.1（0.04,0.23） | 1（0,4） | 0.06（0.02,0.14） | -0.94(-1.22,-0.65) |
| Cameroon | 19（7,43） | 0.0（0.0,0.01） | 29（10,67） | 0.0（0.0,0.0） | -3.77(-4.23,-3.30) |
| Canada | 0（0,1） | 0.07（0.03,0.13） | 0（0,0） | 0.06（0.02,0.14） | -0.02(-0.20,0.17) |
| Central African Republic | 3（1,6） | 0.11（0.05,0.25） | 4（1,11） | 0.09（0.04,0.2） | -0.60(-0.68,-0.52) |
| Chad | 13（5,29） | 0.01（0.01,0.01） | 31（13,69） | 0.0（0.0,0.01） | -4.18(-5.17,-3.18) |
| Chile | 1（0,1） | 0.03（0.01,0.04） | 0（0,0） | 0.01（0.0,0.01） | -2.55(-3.06,-2.03) |
| China | 314（162,487） | 0.03（0.02,0.05） | 70（29,106） | 0.02（0.01,0.03） | -0.15(-1.13,0.83) |
| Colombia | 13（9,19） | 0.22（0.1,0.48） | 5（3,8） | 0.19（0.08,0.38） | -0.63(-0.88,-0.38) |
| Comoros | 1（0,3） | 0.05（0.02,0.12） | 1（0,3） | 0.03（0.01,0.07） | -1.49(-1.71,-1.26) |
| Congo | 2（0,4） | 0.01（0.0,0.03） | 1（0,4） | 0.03（0.01,0.1） | 0.17(-1.29,1.64) |
| Cook Islands | 0（0,0） | 0.03（0.02,0.05） | 0（0,0） | 0.01（0.01,0.02） | -2.85(-3.53,-2.17) |
| Costa Rica | 1（0,2） | 0.03（0.01,0.07） | 0（0,0） | 0.02（0.01,0.05） | -2.64(-4.91,-0.30) |
| Croatia | 0（0,0） | 0.0（0.0,0.0） | 0（0,0） | 0.0（0.0,0.0） | -9.21(-11.24,-7.14) |
| Cuba | 1（0,2） | 0.02（0.01,0.03） | 0（0,0） | 0.0（0.0,0.0） | 1.83(1.30,2.37) |
| Cyprus | 0（0,0） | 0.0（0.0,0.0） | 0（0,0） | 0.0（0.0,0.0） | -2.15(-3.13,-1.15) |
| Czechia | 0（0,0） | 0.0（0.0,0.01） | 0（0,0） | 0.0（0.0,0.0） | -0.62(-0.95,-0.28) |
| Democratic People's Republic of Korea | 4（2,9） | 0.02（0.01,0.04） | 2（1,5） | 0.02（0.01,0.04） | -0.31(-0.45,-0.16) |
| Democratic Republic of the Congo | 33（14,64） | 0.05（0.02,0.09） | 38（14,86） | 0.03（0.01,0.06） | -1.12(-1.43,-0.80) |
| Denmark | 0（0,0） | 0.01（0.01,0.01） | 0（0,0） | 0.0（0.0,0.01） | -3.01(-4.04,-1.96) |
| Djibouti | 1（0,2） | 0.25（0.11,0.46） | 2（0,4） | 0.16（0.06,0.33） | -1.00(-1.48,-0.53) |
| Dominica | 0（0,0） | 0.0（0.0,0.0） | 0（0,0） | 0.0（0.0,0.0） | 0.16(-0.26,0.59) |
| Dominican Republic | 1（0,2） | 0.01（0.0,0.03） | 0（0,1） | 0.01（0.0,0.01） | -1.03(-1.85,-0.20) |
| Ecuador | 7（4,11） | 0.05（0.03,0.09） | 7（3,12） | 0.04（0.02,0.07） | 0.26(-0.16,0.68) |
| Egypt | 4（1,10） | 0.01（0.0,0.01） | 3（1,9） | 0.0（0.0,0.01） | -1.64(-3.23,-0.03) |
| El Salvador | 1（0,2） | 0.02（0.01,0.04） | 0（0,0） | 0.01（0.0,0.01） | -2.86(-2.91,-2.80) |
| Equatorial Guinea | 0（0,1） | 0.06（0.02,0.13） | 0（0,0） | 0.02（0.0,0.05） | -4.29(-4.61,-3.97) |
| Eritrea | 15（5,31） | 0.25（0.09,0.53） | 20（7,41） | 0.22（0.08,0.46） | -0.56(-0.82,-0.29) |
| Estonia | 0（0,0） | 0.01（0.0,0.01） | 0（0,0） | 0.0（0.0,0.0） | -3.10(-4.53,-1.65) |
| Eswatini | 0（0,0） | 0.03（0.01,0.06） | 0（0,0） | 0.03（0.01,0.07） | 0.65(0.38,0.92) |
| Ethiopia | 132（59,248） | 0.14（0.06,0.27） | 115（60,193） | 0.07（0.04,0.12） | -2.40(-2.79,-2.01) |
| Fiji | 0（0,0） | 0.01（0.0,0.03） | 0（0,0） | 0.01（0.0,0.04） | 0.80(0.29,1.31) |
| Finland | 0（0,0） | 0.01（0.0,0.01） | 0（0,0） | 0.01（0.0,0.01） | -1.20(-1.93,-0.46) |
| France | 2（2,3） | 0.01（0.01,0.01） | 1（0,1） | 0.0（0.0,0.0） | -2.39(-3.36,-1.40) |
| Gabon | 0（0,1） | 0.03（0.01,0.08） | 0（0,1） | 0.02（0.01,0.06） | -0.69(-0.98,-0.40) |
| Gambia | 1（0,3） | 0.11（0.05,0.23） | 3（1,7） | 0.1（0.04,0.22） | -0.36(-0.74,0.02) |
| Georgia | 0（0,0） | 0.0（0.0,0.01） | 0（0,1） | 0.02（0.0,0.05） | 9.99(8.60,11.39) |
| Germany | 2（1,3） | 0.0（0.0,0.01） | 0（0,0） | 0.0（0.0,0.0） | -2.84(-3.31,-2.36) |
| Ghana | 20（8,50） | 0.08（0.03,0.2） | 23（8,55） | 0.05（0.02,0.12） | -1.13(-1.44,-0.82) |
| Greece | 0（0,0） | 0.0（0.0,0.0） | 0（0,0） | 0.0（0.0,0.0） | 1.09(-0.48,2.68) |
| Greenland | 0（0,0） | 0.01（0.0,0.02） | 0（0,0） | 0.0（0.0,0.0） | -3.43(-4.53,-2.32) |
| Grenada | 0（0,0） | 0.0（0.0,0.0） | 0（0,0） | 0.0（0.0,0.0） | -7.63(-9.58,-5.64) |
| Guam | 0（0,0） | 0.01（0.0,0.02） | 0（0,0） | 0.01（0.0,0.02） | 1.94(1.38,2.51) |
| Guatemala | 6（2,13） | 0.04（0.02,0.09） | 3（1,5） | 0.02（0.01,0.04） | -1.83(-1.99,-1.66) |
| Guinea | 25（11,52） | 0.23（0.11,0.48） | 30（12,69） | 0.14（0.05,0.32） | -1.23(-1.46,-1.00) |
| Guinea-Bissau | 2(1,6) | 0.15（0.06,0.36） | 2（0,6） | 0.08（0.03,0.19） | -1.43(-1.89,-0.97) |
| Guyana | 0（0,0） | 0.0（0.0,0.0） | 0（0,0） | 0.01（0.0,0.02） | 3.25(-0.09,6.69) |
| Haiti | 0（0,1） | 0.01（0.0,0.02） | 0（0,1） | 0.0（0.0,0.01） | -1.59(-1.74,-1.44) |
| Honduras | 3（1,6） | 0.04（0.02,0.08） | 2（1,5） | 0.03（0.01,0.05） | -1.50(-1.62,-1.39) |
| Hungary | 0（0,0） | 0.01（0.0,0.01） | 0（0,0） | 0.0（0.0,0.01） | -0.60(-1.96,0.78) |
| Iceland | 0（0,0） | 0.01（0.0,0.01） | 0（0,0） | 0.01（0.0,0.01） | 1.13(-0.52,2.82) |
| India | 681（311,1031） | 0.06（0.03,0.09） | 395（233,609） | 0.04（0.02,0.05） | -1.76(-2.00,-1.53) |
| Indonesia | 24（6,42） | 0.01（0.0,0.02） | 18（4,33） | 0.01（0.0,0.02） | -0.67(-0.87,-0.47) |
| Iran (Islamic Republic of) | 0（0,1） | 0.0（0.0,0.0） | 0（0,0） | 0.0（0.0,0.0） | 1.14(0.39,1.89) |
| Iraq | 5（2,10） | 0.02（0.01,0.04） | 7（3,13） | 0.02（0.01,0.03） | -0.53(-0.76,-0.29) |
| Ireland | 0（0,0） | 0.0（0.0,0.0） | 0（0,0） | 0.0（0.0,0.0） | -4.77(-5.02,-4.51) |
| Israel | 0（0,0） | 0.0（0.0,0.0） | 0（0,0） | 0.0（0.0,0.0） | -1.07(-1.84,-0.29) |
| Italy | 1（1,1） | 0.0（0.0,0.0） | 0（0,1） | 0.0（0.0,0.0） | -2.13(-3.85,-0.38) |
| Jamaica | 0（0,1） | 0.02（0.01,0.05） | 0（0,0） | 0.0（0.0,0.01） | -4.02(-5.39,-2.63) |
| Japan | 2（2,3） | 0.0（0.0,0.0） | 0（0,0） | 0.0（0.0,0.0） | -2.29(-3.61,-0.95) |
| Jordan | 0（0,1） | 0.01（0.01,0.02） | 0（0,1） | 0.01（0.0,0.01） | -3.13(-3.57,-2.69) |
| Kazakhstan | 6（1,15） | 0.03（0.01,0.08） | 2（0,5） | 0.01（0.0,0.03） | -3.43(-4.32,-2.52) |
| Kenya | 224（118,338） | 0.54（0.28,0.81） | 214（122,340） | 0.36（0.2,0.57） | -0.40(-0.78,-0.03) |
| Kiribati | 0（0,0） | 0.02（0.0,0.06） | 0（0,0） | 0.01（0.0,0.05） | -1.06(-1.42,-0.71) |
| Kuwait | 0（0,0） | 0.01（0.01,0.02） | 0（0,0） | 0.0（0.0,0.0） | -10.98(-15.00,-6.77) |
| Kyrgyzstan | 2（0,5） | 0.03（0.01,0.08） | 1（0,3） | 0.02（0.01,0.05） | 2.08(-0.25,4.47) |
| Lao People's Democratic Republic | 1（0,2） | 0.02（0.0,0.04） | 0（0,2） | 0.01（0.0,0.03） | -1.24(-1.43,-1.06) |
| Latvia | 0（0,0） | 0.01（0.0,0.02） | 0（0,0） | 0.0（0.0,0.0） | -9.37(-9.71,-9.03) |
| Lebanon | 0（0,0） | 0.01（0.0,0.01） | 0（0,0） | 0.0（0.0,0.01） | -1.47(-1.70,-1.25) |
| Lesotho | 0（0,1） | 0.03（0.01,0.07） | 0（0,1） | 0.04（0.02,0.09） | 1.15(0.90,1.39) |
| Liberia | 4（1,10） | 0.1（0.04,0.23） | 3（1,10） | 0.05（0.02,0.13） | -1.95(-2.66,-1.23) |
| Libya | 1（0,3） | 0.02（0.01,0.05） | 0（0,1） | 0.01（0.01,0.03） | -1.94(-2.12,-1.75) |
| Lithuania | 0（0,0） | 0.01（0.0,0.02） | 0（0,0） | 0.0（0.0,0.0） | -9.06(-10.40,-7.69) |
| Luxembourg | 0（0,0） | 0.0（0.0,0.0） | 0（0,0） | 0.0（0.0,0.0） | -2.61(-4.26,-0.92) |
| Madagascar | 44（19,90） | 0.21（0.09,0.44） | 62（23,136） | 0.15（0.06,0.34） | -0.98(-1.12,-0.83) |
| Malawi | 99（49,186） | 0.55（0.27,1.05） | 108（44,239） | 0.4（0.16,0.88） | -0.61(-0.93,-0.30) |
| Malaysia | 5（2,13） | 0.02（0.01,0.06） | 3（1,6） | 0.01（0.0,0.03） | -1.92(-2.42,-1.42) |
| Maldives | 0（0,0） | 0.0（0.0,0.01） | 0（0,0） | 0.0（0.0,0.0） | -2.24(-2.42,-2.05) |
| Mali | 39（17,83） | 0.25（0.11,0.52） | 51（17,148） | 0.12（0.04,0.34） | -2.17(-2.34,-1.99) |
| Malta | 0（0,0） | 0.0（0.0,0.0） | 0（0,0） | 0.0（0.0,0.0） | -2.84(-3.88,-1.78) |
| Marshall Islands | 0（0,0） | 0.01（0.0,0.03） | 0（0,0） | 0.01（0.0,0.04） | 0.79(0.44,1.14) |
| Mauritania | 3（1,8） | 0.09（0.04,0.23） | 3（1,6） | 0.05（0.02,0.11） | -2.47(-2.66,-2.29) |
| Mauritius | 0（0,0） | 0.0（0.0,0.0） | 0（0,0） | 0.0（0.0,0.0） | 4.92(1.61,8.34) |
| Mexico | 53（45,63） | 0.05（0.04,0.06） | 20（12,29） | 0.02（0.01,0.03） | -2.95(-3.39,-2.50) |
| Micronesia (Federated States of) | 0（0,0） | 0.01（0.0,0.04） | 0（0,0） | 0.01（0.0,0.04） | -0.36(-0.66,-0.06) |
| Monaco | 0（0,0） | 0.0（0.0,0.0） | 0（0,0） | 0.0（0.0,0.0） | -0.60(-1.60,0.41) |
| Mongolia | 0（0,2） | 0.02（0.0,0.06） | 0（0,2） | 0.03（0.01,0.06） | 0.57(0.26,0.88) |
| Montenegro | 0（0,0） | 0.0（0.0,0.0） | 0（0,0） | 0.0（0.0,0.0） | -2.36(-3.08,-1.64) |
| Morocco | 35（16,70） | 0.1（0.05,0.2） | 18（8,36） | 0.06（0.02,0.11） | -1.41(-1.65,-1.16) |
| Mozambique | 80（38,160） | 0.34（0.17,0.69） | 109（51,209） | 0.21（0.1,0.41） | -1.09(-1.38,-0.81) |
| Myanmar | 9（2,26） | 0.02（0.0,0.05） | 7（1,19） | 0.01（0.0,0.04） | -1.34(-1.49,-1.18) |
| Namibia | 2（0,4） | 0.1（0.04,0.21） | 3（1,6） | 0.12（0.06,0.25） | 1.29(0.87,1.70) |
| Nauru | 0（0,0） | 0.02（0.0,0.05） | 0（0,0） | 0.02（0.0,0.05） | 0.04(-0.29,0.36) |
| Nepal | 21（10,49） | 0.07（0.03,0.16） | 12（5,24） | 0.04（0.02,0.08） | -1.51(-1.75,-1.27) |
| Netherlands | 0（0,0） | 0.01（0.0,0.01） | 0（0,0） | 0.0（0.0,0.0） | -2.56(-3.30,-1.80) |
| New Zealand | 0（0,0） | 0.0（0.0,0.0） | 0（0,0） | 0.0（0.0,0.0） | -3.54(-7.26,0.32) |
| Nicaragua | 4（1,12） | 0.07（0.03,0.19） | 1（0,2） | 0.02（0.01,0.04） | -2.98(-3.42,-2.53) |
| Niger | 24（10,61） | 0.15（0.06,0.39） | 38（15,95） | 0.08（0.03,0.19） | -2.13(-2.41,-1.85) |
| Nigeria | 146（66,231） | 0.1（0.04,0.16） | 284（100,500） | 0.08（0.03,0.14） | -0.71(-0.83,-0.58) |
| Niue | 0（0,0） | 0.02（0.0,0.06） | 0（0,0） | 0.06（0.02,0.21） | 0.79(-0.40,1.99) |
| North Macedonia | 0（0,0） | 0.0（0.0,0.0） | 0（0,0） | 0.0（0.0,0.0） | -1.12(-1.88,-0.36) |
| Northern Mariana Islands | 0（0,0） | 0.0（0.0,0.01） | 0（0,0） | 0.0（0.0,0.0） | -1.25(-1.76,-0.73) |
| Norway | 0（0,0） | 0.0（0.0,0.0） | 0（0,0） | 0.0（0.0,0.0） | -8.43(-10.22,-6.62) |
| Oman | 0（0,0） | 0.01（0.0,0.02） | 0（0,0） | 0.0（0.0,0.01） | -2.73(-3.27,-2.20) |
| Pakistan | 93（42,177） | 0.05（0.02,0.1） | 212（95,378） | 0.07（0.03,0.13） | 1.67(1.43,1.91) |
| Palau | 0（0,0） | 0.01（0.0,0.03） | 0（0,0） | 0.01（0.0,0.03） | -0.05(-0.28,0.17) |
| Palestine | 0（0,0） | 0.0（0.0,0.01） | 0（0,0） | 0.0（0.0,0.0） | -0.80(-1.43,-0.16) |
| Panama | 0（0,2） | 0.03（0.02,0.07） | 0（0,1） | 0.02（0.01,0.03） | -1.58(-1.80,-1.35) |
| Papua New Guinea | 0（0,3） | 0.01（0.0,0.05） | 2（0,8） | 0.02（0.0,0.06） | 0.35(-0.07,0.77) |
| Paraguay | 2（0,5） | 0.04（0.02,0.08） | 1（0,4） | 0.03（0.01,0.07） | -0.54(-0.80,-0.28) |
| Peru | 28（14,52） | 0.1（0.05,0.18） | 9（4,20） | 0.03（0.01,0.06） | -3.14(-3.36,-2.92) |
| Philippines | 25（6,45） | 0.03（0.01,0.05） | 34（9,58） | 0.03（0.01,0.05） | 0.76(0.48,1.05) |
| Poland | 0（0,0） | 0.0（0.0,0.0） | 0（0,0） | 0.0（0.0,0.0） | -4.20(-6.91,-1.42) |
| Portugal | 1（1,2） | 0.02（0.02,0.03） | 0（0,0） | 0.01（0.01,0.02） | -3.20(-4.83,-1.54) |
| Puerto Rico | 0（0,0） | 0.01（0.0,0.01） | 0（0,0） | 0.0（0.0,0.0） | -7.22(-9.43,-4.95) |
| Qatar | 0（0,0） | 0.0（0.0,0.0） | 0（0,0） | 0.0（0.0,0.0） | -4.88(-7.66,-2.02) |
| Republic of Korea | 4（2,7） | 0.01（0.01,0.02） | 0（0,1） | 0.0（0.0,0.01） | -3.99(-4.43,-3.54) |
| Republic of Moldova | 1（0,3） | 0.04（0.02,0.09） | 0（0,0） | 0.01（0.0,0.01） | -6.30(-6.72,-5.88) |
| Romania | 6（2,14） | 0.04（0.01,0.08） | 0（0,1） | 0.01（0.0,0.01） | -6.34(-7.07,-5.60) |
| Russian Federation | 17（12,24） | 0.02（0.01,0.02） | 2（2,3） | 0.0（0.0,0.0） | -6.26(-7.28,-5.22) |
| Rwanda | 36（17,76） | 0.28（0.13,0.58） | 24（9,49） | 0.14（0.05,0.29） | -1.99(-2.35,-1.62) |
| Saint Kitts and Nevis | 0（0,0） | 0.0（0.0,0.0） | 0（0,0） | 0.0（0.0,0.0） | -8.73(-10.35,-7.07) |
| Saint Lucia | 0（0,0） | 0.0（0.0,0.0） | 0（0,0） | 0.0（0.0,0.0） | -4.71(-5.94,-3.46) |
| Saint Vincent and the Grenadines | 0（0,0） | 0.11（0.05,0.26） | 0（0,0） | 0.01（0.0,0.02） | -8.17(-9.77,-6.54) |
| Samoa | 0（0,0） | 0.03（0.01,0.06） | 0（0,0） | 0.02（0.01,0.05） | -1.09(-1.30,-0.89) |
| San Marino | 0（0,0） | 0.0（0.0,0.0） | 0（0,0） | 0.0（0.0,0.0） | -1.99(-2.33,-1.64) |
| Sao Tome and Principe | 0（0,0） | 0.11（0.04,0.27） | 0（0,0） | 0.05（0.02,0.13） | -1.77(-2.81,-0.71) |
| Saudi Arabia | 4（0,9） | 0.02（0.0,0.04） | 1（0,2） | 0.0（0.0,0.01） | -3.80(-5.38,-2.19) |
| Senegal | 18（7,44） | 0.13（0.05,0.32） | 17（7,41） | 0.08（0.03,0.19） | -1.03(-1.46,-0.60) |
| Serbia | 0（0,1） | 0.01（0.0,0.01） | 0（0,0） | 0.0（0.0,0.0） | -5.74(-6.44,-5.05) |
| Seychelles | 0（0,0） | 0.0（0.0,0.0） | 0（0,0） | 0.0（0.0,0.0） | -1.54(-1.76,-1.33) |
| Sierra Leone | 7（3,17） | 0.11（0.04,0.24） | 9（3,21） | 0.07（0.03,0.17） | -1.18(-1.44,-0.91) |
| Singapore | 0（0,0） | 0.03（0.02,0.04） | 0（0,0） | 0.01（0.0,0.01） | -4.75(-5.40,-4.11) |
| Slovakia | 0（0,0） | 0.0（0.0,0.01） | 0（0,0） | 0.0（0.0,0.01） | 0.71(-0.25,1.69) |
| Slovenia | 0（0,0） | 0.0（0.0,0.0） | 0（0,0） | 0.0（0.0,0.0） | -0.21(-1.01,0.60) |
| Solomon Islands | 0（0,0） | 0.01（0.0,0.04） | 0（0,0） | 0.01（0.0,0.05） | 0.51(-0.15,1.16) |
| Somalia | 35（15,78） | 0.24（0.1,0.54） | 58（17,141） | 0.15（0.04,0.35） | -1.03(-1.49,-0.56) |
| South Africa | 9（4,17） | 0.02（0.01,0.04） | 11（3,22） | 0.02（0.01,0.04） | 0.73(-0.05,1.51) |
| South Sudan | 20（8,40） | 0.21（0.09,0.42） | 20（8,39） | 0.13（0.06,0.26） | -0.71(-0.97,-0.46) |
| Spain | 1（1,1） | 0.01（0.01,0.01） | 0（0,0） | 0.0（0.0,0.0） | -2.56(-3.70,-1.41) |
| Sri Lanka | 2（1,4） | 0.01（0.01,0.03） | 0（0,2） | 0.01（0.0,0.01） | -1.63(-2.19,-1.06) |
| Sudan | 0（0,1） | 0.0（0.0,0.0） | 0（0,1） | 0.0（0.0,0.0） | -1.48(-1.73,-1.22) |
| Suriname | 0（0,0） | 0.0（0.0,0.0） | 0（0,0） | 0.0（0.0,0.0） | -0.19(-0.78,0.40) |
| Sweden | 0（0,0） | 0.0（0.0,0.0） | 0（0,0） | 0.0（0.0,0.0） | 0.46(-3.20,4.27) |
| Switzerland | 0（0,0） | 0.01（0.0,0.01） | 0（0,0） | 0.0（0.0,0.01） | -1.53(-2.19,-0.87) |
| Syrian Arab Republic | 0（0,0） | 0.0（0.0,0.0） | 0（0,0） | 0.0（0.0,0.0） | -2.45(-2.71,-2.18) |
| Taiwan | 0（0,0） | 0.0（0.0,0.01） | 0（0,0） | 0.01（0.0,0.01） | 2.65(1.73,3.58) |
| Tajikistan | 0（0,1） | 0.01（0.0,0.02） | 1（0,4） | 0.01（0.0,0.04） | 1.71(1.34,2.07) |
| Thailand | 12（6,21） | 0.02（0.01,0.04） | 1（0,3） | 0.01（0.0,0.01） | -4.29(-4.82,-3.77) |
| Timor-Leste | 0(0,0) | 0.01（0.0,0.03） | 0（0,0） | 0.01（0.0,0.03） | -0.67(-0.91,-0.43) |
| Togo | 6（2,15） | 0.1（0.04,0.24） | 7（2,17） | 0.06（0.02,0.15） | -1.37(-1.52,-1.22) |
| Tokelau | 0(0,0) | 0.01（0.0,0.03） | 0（0,0） | 0.37（0.08,1.23） | 3.17(-0.38,6.85) |
| Tonga | 0（0,0） | 0.01（0.0,0.03） | 0（0,0） | 0.01（0.0,0.04） | 0.52(0.11,0.94) |
| Trinidad and Tobago | 0（0,0） | 0.03（0.01,0.07） | 0（0,0） | 0.02（0.01,0.05） | 0.17(-1.13,1.49) |
| Tunisia | 3（1,6） | 0.03（0.02,0.06） | 1（0,2） | 0.01（0.01,0.02） | -3.01(-3.25,-2.78) |
| Türkiye | 12（6,22） | 0.02（0.01,0.03） | 4（2,8） | 0.01（0.0,0.01） | -1.20(-1.36,-1.04) |
| Turkmenistan | 0（0,1） | 0.01（0.0,0.03） | 0（0,1） | 0.01（0.0,0.02） | -0.98(-1.39,-0.57) |
| Tuvalu | 0（0,0） | 0.02（0.0,0.06） | 0（0,0） | 0.01（0.0,0.04） | -2.49(-2.85,-2.13) |
| Uganda | 71（31,130） | 0.21（0.09,0.38） | 102（45,221） | 0.14（0.06,0.31） | -0.92(-1.22,-0.63) |
| Ukraine | 3（1,5） | 0.01（0.0,0.01） | 1（0,2） | 0.01（0.0,0.01） | -0.24(-0.62,0.15) |
| United Arab Emirates | 0（0,0） | 0.01（0.0,0.01） | 0（0,0） | 0.0（0.0,0.01） | 0.05(-0.58,0.68) |
| United Kingdom | 0（0,0） | 0.0（0.0,0.0） | 0（0,0） | 0.0（0.0,0.0） | -0.48(-2.32,1.39) |
| United Republic of Tanzania | 114（47,242） | 0.25（0.1,0.53） | 152（63,314） | 0.17（0.07,0.36） | -0.52(-0.80,-0.24) |
| United States of America | 13（11,14） | 0.01（0.01,0.01） | 4（3,5） | 0.0（0.0,0.0） | -2.96(-3.60,-2.32) |
| United States Virgin Islands | 0（0,0） | 0.03（0.01,0.07） | 0（0,0） | 0.01（0.0,0.02） | -3.69(-4.10,-3.27) |
| Uruguay | 0（0,1） | 0.02（0.01,0.04） | 0（0,0） | 0.01（0.0,0.01） | -3.15(-3.58,-2.71) |
| Uzbekistan | 3（0,11） | 0.01（0.0,0.03） | 5（1,12） | 0.02（0.01,0.03） | 1.00(0.66,1.34) |
| Vanuatu | 0（0,0） | 0.01（0.0,0.03） | 0（0,0） | 0.01（0.0,0.03） | 0.77(0.07,1.48) |
| Venezuela (Bolivarian Republic of) | 7（3,14） | 0.03（0.01,0.06） | 3（1,7） | 0.02（0.01,0.03） | -1.40(-1.71,-1.09) |
| Vietnam | 70（36,138） | 0.07（0.04,0.15） | 26（11,53） | 0.03（0.01,0.06） | -2.98(-3.13,-2.82) |
| Yemen | 0（0,0） | 0.0（0.0,0.0） | 0（0,1） | 0.0（0.0,0.0） | -0.49(-0.72,-0.25) |
| Zambia | 37（18,74） | 0.26（0.12,0.52） | 40（17,83） | 0.14（0.06,0.29） | -1.88(-2.18,-1.58) |
| Zimbabwe | 6（3,11） | 0.04（0.02,0.07） | 13（4,30） | 0.06（0.02,0.14） | 4.76(3.15,6.39) |

**Abbreviations**: ASR, age-standardized rate; RB, Retinoblastoma; EAPC, estimated annual percentage change; UIs, uncertainty intervals; CI, conﬁdence interval.

**Table S8** Comparison of APC and AAPC for ASIR, ASPR, ASR-DALY, ASDR in RB from 1990 to 2021

| **Incidence** | | | **Prevalence** | | | **DALYs** | | | **DEATH** | | |
| --- | --- | --- | --- | --- | --- | --- | --- | --- | --- | --- | --- |
| **Range** | **APC (95% CI)** | **P** | **Range** | **APC (95% CI)** | **P** | **Range** | **APC (95% CI)** | **P** | **Range** | **APC (95% CI)** | **P** |
| 1990-2003 | 0.44（0.34,0.55） | < 0.001 | 1990-2003 | 0.45（0.35,0.56） | < 0.001 | 1990-1997 | -0.31（-0.55,-0.07） | < 0.001 | 1990-1997 | 0.31（-0.55,-0.07） | < 0.001 |
| 2003-2015 | 2.53（2.40,2.67） | < 0.001 | 2003-2015 | 2.55（2.41,2.68） | < 0.001 | 1997-2002 | -0.71（-1.23,-0.20） | < 0.001 | 1997-2002 | -0.71（-1.23,-0.19） | < 0.001 |
| 2015-2019 | 0.57（-0.48,1.63） | < 0.001 | 2015-2019 | 0.58（-0.47,1.64） | < 0.001 | 2002-2014 | 0.11（0.01,-0.22 | < 0.001 | 2002-2014 | 0.08（-0.02,0.17） | < 0.001 |
| 2019-2021 | -8.99（-11.09,-6.83） | < 0.001 | 2019-2021 | -8.99（-11.09,-6.84） | < 0.001 | 2014-2019 | -1.90（-2.41,-1.38） | < 0.001 | 2014-2019 | -1.94(-2.47,-1.41） | < 0.001 |
|  |  |  |  |  |  | 2019-2021 | -3.72（-5.47,-1.94） | < 0.001 | 2019-2021 | -3.58（-5.36,-1.76） | < 0.001 |
|  | | | | | | | | |  |  |  |
| Range | AAPC (95% CI) | *P* | Range | AAPC (95% CI) | *P* | Range | AAPC (95% CI) | *P* |  | AAPC (95% CI) | *P* |
| 1990-2021 | 0.62(0.42,0.82) | < 0.001 | 1990-2021 | 0.63(0.43,0.84) | < 0.001 | 1990-2021 | -0.69(-0.86,-0.52) | < 0.001 | 1990-2021 | -0.70(-0.87,-0.531 | < 0.001 |

**Abbreviations**: APC, annual percentage change; AAPC, average annual percentage change; RB, Retinoblastoma; DALYs, disability-adjusted life-years; CI, conﬁdence interval.

**Table S9** Forecasting the Numbers of Incidence, Prevalence, DALYs, and Deaths for RB from 2022 to 2035

| **Year** | **Incidence (95% Crl)** | **Prevalence (95% Crl)** | **DALYs (95% Crl)** | **DEATH (95% Crl)** |
| --- | --- | --- | --- | --- |
| 2022 | 6034（5661，6407 ） | 55127（51715，58538） | 236107（230480，241734） | 2683（2622，2745） |
| 2023 | 5889（5185，6592） | 53801（47362，60241） | 233733（222838，244628） | 2655（2534，2776） |
| 2024 | 5801（4786，6817） | 53004（43716，62292） | 236569（219915，253224） | 2682（2497，2868） |
| 2025 | 5749（4449，7049） | 52526（40629，64422） | 239524（218016，261032） | 2713（2473，2954） |
| 2026 | 5718（4158，7277） | 52238（37969，66507） | 242177（217439，266914） | 2741（2464，3019） |
| 2027 | 5699（3903，7494） | 52065（35635，68495） | 244559（217497，271621） | 2767（2462，3072） |
| 2028 | 5688（3676，7699） | 51961（33551，70372） | 246698（217898，275499） | 2790（2464，3116） |
| 2029 | 5681（3469，7892） | 51899（31662，72136） | 248619（218490，278748） | 2811（2469，3153） |
| 2030 | 5677（3280，8073） | 51861（29927，73796） | 250344（219185，281503） | 2830（2475，3185） |
| 2031 | 5674（3104，8244） | 51839（28316，75361） | 251893（219928，283858） | 2847（2482，3212） |
| 2032 | 5889（5185，6592） | 55127（51715，58538） | 236107（230480，241734） | 2683（2622，2745） |
| 2033 | 5801（4786，6817） | 53801（47362，60241） | 233733（222838，244628） | 2655（2534，2776） |
| 2034 | 5749（4449，7049） | 53004（43716，62292） | 236569（219915，253224） | 2682（2497，2868） |
| 2035 | 5718（4158，7277） | 52526（40629，64422） | 239524（218016，261032） | 2713（2473，2954） |

**Abbreviations:** DALYs, disability-adjusted life-years; RB, Retinoblastoma; Crl, credible interval.

Table S10 Rare disease policy releases in different policy domains across countries at different levels of economic development

| **Country** | **Rare Disease Management** | **Prevention of Rare Diseases** | **Rare Disease Diagnosis and Treatment** | **Rare Disease R&D, Registration** | **Rare Disease medical insurance** | **Other Rare Disease-Related Policies** | **Total** |
| --- | --- | --- | --- | --- | --- | --- | --- |
| United States of America | 11 | 1 | 3 | 20 | - | 1 | 28 |
| United Kingdom | 9 | 3 | 9 | 4 | 1 | 4 | 19 |
| China | 4 | 2 | 9 | 13 | 2 | - | 29 |
| Brazil | 10 | 1 | 9 | 3 | 2 | 1 | 15 |
| Ethiopia | 1 | 1 | - | 1 | - | - | 3 |
| Uganda | - | 1 | 1 | 1 | - | - | 3 |

Table S11 Number of rare disease policies issued annually by countries at different levels of economic development

| **Year** | **United States of America** | **United Kingdom** | **China** | **Brazil** | **Ethiopia** | **Uganda** |
| --- | --- | --- | --- | --- | --- | --- |
| 1983 | 2 | - | - | - | - | - |
| 1992 | 1 | - | - | - | - | - |
| 1999 | - | 1 | - | - | - | - |
| 2000 | - | 3 | - | - | - | - |
| 2001 | 1 | - | - | 1 | - | - |
| 2002 | 2 | - | - | - | - | - |
| 2008 | - | - | - | - | - | - |
| 2009 | - | 2 | 1 | - | - | - |
| 2010 | 1 | 1 | - | - | - | - |
| 2011 | - | 2 | - | - | - | - |
| 2013 | 1 | 1 | - | - | - | - |
| 2014 | 1 | - | - | 2 | 1 | - |
| 2015 | - | 1 | - | 1 | - | - |
| 2016 | 2 | - | - | 1 | - | - |
| 2017 | 3 | - | 2 | 3 | - | - |
| 2018 | 3 | 1 | 3 | - | - | - |
| 2019 | 3 | - | 4 | - | - | - |
| 2020 | 1 | 2 | 2 | 1 | - | - |
| 2021 | 4 | 1 | 1 | 1 | - | - |
| 2022 | - | 3 | 3 | 1 | - | - |
| 2023 | 2 | - | 6 | 1 | 1 | 1 |
| 2024 | - | - | 4 | 2 | 1 | 2 |
| 2025 | 1 | 1 | 3 | 1 | 0 | 0 |
| Total | 28 | 19 | 29 | 15 | 3 | 3 |

Table S12 Rare disease policy release status in countries with different levels of economic development

| **No.** | **Country** | **Publicati--on Year** | **Issuing Agency** | **Policy Title** | **Policy Domain** | **URL** |
| --- | --- | --- | --- | --- | --- | --- |
| 1 | United States of America | 1983 | The United States Congress (97th Congress) | 《Orphan Drug Act》 | Management, R&D & Registration | [https://www.congress.gov/bill/97th-congress/house-bill/5238/text?s=1&r=1&q=%7B%22search%22%3A%22Orphan+Drug+Act%22%7D](https://www.congress.gov/bill/97th-congress/house-bill/5238/text?s=1&r=1&q={"search":"Orphan+Drug+Act"}" \o "https://www.congress.gov/bill/97th-congress/house-bill/5238/text?s=1&r=1&q=%7B%22search%22%3A%22Orphan+Drug+Act%22%7D) |
| 2 | United States of America | 1983 | FDA | 《Clinical Trials GrantsProgram》 | R&D & Registration | <https://www.fda.gov/industry/medical-products-rare-diseases-and-conditions/grant-programs-support-development-medical-products-rare-diseases> |
| 3 | United States of America | 1992 | FDA | 《Code of Federal Regulations-ORPHAN DRUGS》 | Management | <https://www.fda.gov/media/86619/download> |
| 4 | United States of America | 2001 | The United States Congress (107th Congress) | 《Muscular Dystrophy Community Assistance, Research and Education Amendments of 2001》 | Management, Diagnosis and Treatment | [https://www.govinfo.gov/content/pkg/PLAW-107publ84/pdf/PLAW-107publ84.pdf](https://www.govinfo.gov/content/pkg/PLAW-107publ84/pdf/PLAW-107publ84.pdf) |
| 5 | United States of America | 2002 | The United States Congress (107th Congress) | 《Rare Diseases Orphan Product Development Act of 2002》 | R&D & Registration | <https://www.govinfo.gov/app/details/STATUTE-116/STATUTE-116-Pg1992> |
| 6 | United States of America | 2002 | The United States Congress (107th Congress) | 《Rare Diseases Act of 2002》 | Management, R&D & Registration | <https://www.congress.gov/bill/107th-congress/house-bill/4013/text> |
| 7 | United States of America | 2010 | The United States Congress (111th Congress) | 《Creating Hope Act of 2010》 | Management, R&D & Registration | [https://www.govinfo.gov/content/pkg/BILLS-111s3697is/pdf/BILLS-111s3697is.pdf](https://www.govinfo.gov/content/pkg/BILLS-111s3697is/pdf/BILLS-111s3697is.pdf) |
| 8 | United States of America | 2013 | FDA | 《Orphan Drug Regulations》 | R&D & Registration | [https://www.govinfo.gov/content/pkg/FR-2013-06-12/html/2013-13930.htm](https://www.govinfo.gov/content/pkg/FR-2013-06-12/html/2013-13930.htm) |
| 9 | United States of America | 2014 | The United States Congress (113th Congress) | 《NEWBORN SCREENING SAVES LIVES REAUTHORIZATIONACT OF 2014》 | Management, Prevention | [https://www.govinfo.gov/content/pkg/CRPT-113hrpt478/pdf/CRPT-113hrpt478.pdf](https://www.govinfo.gov/content/pkg/CRPT-113hrpt478/pdf/CRPT-113hrpt478.pdf) |
| 10 | United States of America | 2016 | The United States Congress (114th Congress) | 《Advancing Hope Act of 2016》 | Management, R&D & Registration | <https://www.congress.gov/bill/114th-congress/senate-bill/1878/text> |
| 11 | United States of America | 2016 | FDA | 《Natural History StudiesGrants Program》 | R&D & Registration | https://www.fda.gov/industry/orphan-products-grants-program/natural-history-studies-grants-program |
| 12 | United States of America | 2017 | FDA | 《Orphan Drug Modernization Plan》 | R&D & Registration | <https://www.fda.gov/industry/designating-orphan-product-drugs-and-biological-products/orphan-drug-modernization-plan> |
| 13 | United States of America | 2017 | FDA | 《Pediatric Rare Diseases--A Collaborative Approach for Drug Development Using Gaucher Disease as a Model》 | R&D & Registration | <https://www.fda.gov/regulatory-information/search-fda-guidance-documents/pediatric-rare-diseases-collaborative-approach-drug-development-using-gaucher-disease-model-draft> |
| 14 | United States of America | 2017 | FDA | 《Clarification of Orphan Designation of Drugs and Biologics for Pediatric Subpopulations of Common Diseases Guidance for Industry》 | Management | <https://www.fda.gov/regulatory-information/search-fda-guidance-documents/clarification-orphan-designation-drugs-and-biologics-pediatric-subpopulations-common-diseases> |
| 15 | United States of America | 2018 | FDA | 《Slowly Progressive, Low-Prevalence Rare Diseases With Substrate Deposition That Results From Single Enzyme Defects: Providing Evidence Of Effectiveness for Replacement or Corrective Therapies》 | Management | https://www.regulations.gov/document/FDA-2018-D-2456-0002 |
| 16 | United States of America | 2018 | FDA | 《Rare Diseases: Early Drug Development and the Role of Pre-IND Meetings Guidance for Industry DRAFT GUIDANCE》 | R&D & Registration | https://www.regulations.gov/document/FDA-2018-D-3268-0002 |
| 17 | United States of America | 2018 | The United States Congress (115th Congress) | 《THE RARE DISEASE ADVANCEMENT, RESEARCH, AND EDUCATION (RARE) ACT》 | Diagnosis and Treatment, Other Rare Disease-Related Policies | https://www.congress.gov/congressional-record/volume-164/issue-35/extensions-of-remarks-section/article/E232-2?q=%7B%22search%22%3A%22rare+disease%22%7D&s=2&r=3 |
| 18 | United States of America | 2019 | FDA | 《Rare Diseases: Natural History Studies for Drug Development》 | R&D & Registration | <https://www.fda.gov/regulatory-information/search-fda-guidance-documents/rare-diseases-natural-history-studies-drug-development> |
| 19 | United States of America | 2019 | FDA | 《Rare Pediatric Disease Priority Review Vouchers》 | R&D & Registration | https://www.fda.gov/regulatory-information/search-fda-guidance-documents/rare-pediatric-disease-priority-review-vouchers |
| 20 | United States of America | 2019 | FDA | 《Rare Pediatric Disease Priority Review Vouchers Guidance for Industry》 | R&D & Registration | [https://www.fda.gov/regulatory-information/search-fda-guidance-documents/rare-pediatric-disease-priority-review-vouchers](https://www.fda.gov/regulatory-information/search-fda-guidance-documents/rare-pediatric-disease-priority-review-vouchers) |
| 21 | United States of America | 2020 | HHS | 《Human Gene Therapy for Rare Diseases 》 | R&D & Registration | [https://www.fda.gov/media/113807/download](https://www.fda.gov/media/113807/download) |
| 22 | United States of America | 2021 | The United States Congress (117th Congress) | 《S.Res.74》 | Management | https://www.congress.gov/bill/117th-congress/senate-resolution/74?q=%7B%22search%22%3A%22S.+RES.+74%22%7D&s=1&r=10 |
| 23 | United States of America | 2021 | The United States Congress (117th Congress) | 《Fairness in Orphan Drug Exclusivity Act》 | Management, R&D & Registration | https://www.congress.gov/bill/117th-congress/house-bill/1629 |
| 24 | United States of America | 2021 | The United States Congress (117th Congress) | 《ACCELERATING ACCESS TO CRITICAL THERAPIES FOR ALS ACT》 | R&D & Registration | [https://www.govinfo.gov/app/details/CRPT-117hrpt207/CRPT-117hrpt207](https://www.govinfo.gov/app/details/CRPT-117hrpt207/CRPT-117hrpt207) |
| 25 | United States of America | 2021 | The United States Congress (117th Congress) | 《Accelerating Access to CriticalTherapies for ALS Act》 | Diagnosis and Treatment | [https://www.congress.gov/117/plaws/publ79/PLAW-117publ79.pdf](https://www.congress.gov/117/plaws/publ79/PLAW-117publ79.pdf) |
| 26 | United States of America | 2023 | FDA | 《Support for Clinical Trials Advancing Rare Disease Therapeutics Pilot Program》 | R&D & Registration | https://www.federalregister.gov/documents/2023/10/02/2023-21235/support-for-clinical-trials-advancing-rare-disease-therapeutics-pilot-program-program-announcement |
| 27 | United States of America | 2023 | FDA | 《Rare Diseases: Considerations for the Development of Drugs and Biological Products》 | R&D & Registration | [https://www.fda.gov/regulatory-information/search-fda-guidance-documents/rare-diseases-considerations-development-drugs-and-biological-products](https://www.fda.gov/regulatory-information/search-fda-guidance-documents/rare-diseases-considerations-development-drugs-and-biological-products) |
| 28 | United States of America | 2025 | FDA | 《CDER/CBER Rare Disease Evidence Principles (RDEP)》 | R&D & Registration | https://www.fda.gov/industry/fda-rare-disease-innovation-hub/cdercber-rare-disease-evidence-principles-rdep |
| 29 | United Kingdom | 1999 | EP, EUC | 《DECISION NO 1295/1999/EC OF THE EUROPEAN PARLIAMENT AND OFTHE COUNCIL》 | Diagnosis and Treatment, Other Rare Disease-Related Policies | <https://eur-lex.europa.eu/resource.html?uri=cellar:208111e4-414e-4da5-94c1-852f1c74f351.0008.02/DOC_1&format=PDF> |
| 30 | United Kingdom | 2000 | EC | 《COMMISSION REGULATION (EC) No 847/2000》 | Management, R&D & Registration | [https://eur-lex.europa.eu/legal-content/EN/TXT/?uri=CELEX%3A02000R0847-20180619](https://eur-lex.europa.eu/legal-content/EN/TXT/?uri=CELEX:02000R0847-20180619) |
| 31 | United Kingdom | 2000 | EMA | 《List of the marketing authorisations》 | Management, R&D & Registration | <https://www.eurordis.org/list-of-the-marketing-authorisations/> |
| 32 | United Kingdom | 2000 | EP, EUC | 《REGULATION (EC) No 141/2000 OF THE EUROPEAN PARLIAMENT AND OF THE COUNCIL》 | Management, R&D & Registration | [https://eur-lex.europa.eu/legal-content/EN/TXT/?uri=CELEX%3A02000R0141-20190726](https://eur-lex.europa.eu/legal-content/EN/TXT/?uri=CELEX:02000R0141-20190726) |
| 33 | United Kingdom | 2009 | EUC | 《on an action in the field of rare diseases》 | Diagnosis and Treatment | <https://eur-lex.europa.eu/legal-content/EN/TXT/?uri=CELEX:32009H0703(02)> |
| 34 | United Kingdom | 2009 | EC | 《establishing a European Union Committee of Experts on Rare Diseases》 | Management | <https://eur-lex.europa.eu/legal-content/EN/TXT/?uri=CELEX:32009D0872> |
| 35 | United Kingdom | 2010 | DG SANTE | 《RECOMMENDATIONSFOR THE DEVELOPMENT OF NATIONAL PLANS FOR RARE DISEASES》 | Prevention, Diagnosis and Treatment, Other Rare Disease-Related Policies | https://health.ec.europa.eu/latest-updates/national-plans-or-strategies-rare-diseases-page-updated-1970-01-01_en |
| 36 | United Kingdom | 2011 | NHSE | 《Cancer Drugs Fund》 | Other Rare Disease-Related Policies | <https://www.england.nhs.uk/cancer/cdf/> |
| 37 | United Kingdom | 2011 | EP, EUC | 《on the application of patients' rights in cross-border healthcare》 | Management, Diagnosis and Treatment | https://eur-lex.europa.eu/eli/dir/2011/24/oj |
| 38 | United Kingdom | 2013 | DHSC | 《Rare diseases: UK strategy》 | Management, Diagnosis and Treatment | <https://www.gov.uk/government/collections/rare-diseases> |
| 39 | United Kingdom | 2015 | NHSE | 《National Congenital Anomaly and Rare Disease Registration Service》 | Management | https://eu-rd-platform.jrc.ec.europa.eu/node/1300_it |
| 40 | United Kingdom | 2018 | NHSE | 《Implementation Plan for the UK Strategy for Rare Diseases》 | Management, Diagnosis and Treatment | https://www.england.nhs.uk/commissioning/publication/implementation-plan-for-the-uk-strategy-for-rare-diseases-progress-report/ |
| 41 | United Kingdom | 2020 | DHSC | 《Genome UK: the future of healthcare》 | Prevention | <https://www.gov.uk/government/publications/genome-uk-the-future-of-healthcare> |
| 42 | United Kingdom | 2020 | Medicines and Healthcare products Regulatory Agency | 《Guidance Orphan medicinal products》 | R&D & Registration | <https://www.gov.uk/guidance/orphan-medicinal-products-in-great-britain> |
| 43 | United Kingdom | 2021 | DHSC | 《UK Rare Diseases Framework》 | Management, Diagnosis and Treatment | [https://www.gov.uk/government/publications/uk-rare-diseases-framework](https://www.gov.uk/government/publications/uk-rare-diseases-framework) |
| 44 | United Kingdom | 2022 | DHSC | 《Genome UK: 2022 to 2025 implementation plan for England》 | Prevention | <https://www.gov.uk/government/publications/genome-uk-2022-to-2025-implementation-plan-for-england> |
| 45 | United Kingdom | 2022 | DHSC | 《England Rare Diseases Action Plan》 | Diagnosis and Treatment | [https://www.gov.uk/government/publications/england-rare-diseases-action-plan-2022](https://www.gov.uk/government/publications/england-rare-diseases-action-plan-2022) |
| 46 | United Kingdom | 2022 | NICE | 《The Innovative Medicines Fund Principles》 | Other Rare Disease-Related Policies | <https://www.england.nhs.uk/wp-content/uploads/2022/06/B1686-the-innovate-medicines-fund-principles-june-2022.pdf> |
| 47 | United Kingdom | 2025 | DHSC | 《England Rare Diseases Action Plan 2025》 | Diagnosis and Treatment, medical insurance | https://www.gov.uk/government/publications/england-rare-diseases-action-plan-2025 |
| 48 | China | 2009 | NHC of the PRC | 《Regulations on the Management of Newborn Disease Screening》 | Prevention | [https://www.gov.cn/gongbao/content/2009/content_1371363.htm](https://www.gov.cn/gongbao/content/2009/content_1371363.htm) |
| 49 | China | 2017 | NMPA | 《The Opinions on Encouraging Pharmaceutical Innovation via Priority Review & Approval issued by NMPA》 | R&D & Registration | [http://www.cjpi.org.cn/zryyxxw/yaop/cfdatz/webinfo/2017/12/1515084437146723.htm](http://www.cjpi.org.cn/zryyxxw/yaop/cfdatz/webinfo/2017/12/1515084437146723.htm) |
| 50 | China | 2017 | NPCSC | 《Law of the People's Republic of China on Maternal and Infant Health Care》 | Management, Prevention | [https://www.gov.cn/guoqing/2021-10/29/content_5647619.htm](https://www.gov.cn/guoqing/2021-10/29/content_5647619.htm) |
| 51 | China | 2018 | NMPA | 《Notice on the Release of Guidance Principles for the Registration Review of Medical Devices for the Prevention and Treatment of Rare Diseases》 | R&D & Registration | <https://www.nmpa.gov.cn/xxgk/ggtg/ylqxggtg/ylqxqtggtg/20181018162101924.html?type=pc> |
| 52 | China | 2018 | NHC of the PRC | 《Notice on Issuing the Procedures for Formulating the Rare Diseases Catalog》 | Management | <https://hjb.bjyxh.org.cn/News/Detail/414> |
| 53 | China | 2018 | NHC of the PRC | 《Notice on the Publication of the First Batch of Rare Diseases List》 | Management | <https://www.gov.cn/zhengce/zhengceku/2018-12/31/content_5435167.htm> |
| 54 | China | 2019 | NHC of the PRC | 《China Rare Disease Diagnosis and Treatment Service Information System Work Management Plan》 | Diagnosis and Treatment | <https://www.gov.cn/zhengce/zhengceku/2019-11/13/content_5451656.htm> |
| 55 | China | 2019 | NHC of the PRC | 《Notice on Establishing a National Rare Disease Diagnosis and Treatment Collaborative Network》 | Diagnosis and Treatment | <https://www.gov.cn/zhengce/zhengceku/2019-10/08/content_5436962.htm> |
| 56 | China | 2019 | NMPA | 《Notice on Value-Added Tax Policies for Rare Disease Medicines》 | R&D & Registration | <https://www.gov.cn/zhengce/zhengceku/2019-10/15/content_5439874.htm> |
| 57 | China | 2019 | NHC of the PRC | 《Guidelines for the Diagnosis and Treatment of Rare Diseases in China》 | Diagnosis and Treatment | [https://www.nhc.gov.cn/yzygj/c100068/201902/073540e8f83b4a54a28684d23e2ae2f5.shtml](https://www.nhc.gov.cn/yzygj/c100068/201902/073540e8f83b4a54a28684d23e2ae2f5.shtml) |
| 58 | China | 2020 | NHC of the PRC | 《Notice on Issuing the List of the Second Batch of Experts for Rare Disease Diagnosis, Treatment, and Protection under the National Health Commission》 | Diagnosis and Treatment | [https://www.nhc.gov.cn/yzygj/c100068/202009/10ffeb2e074b4d6b9567ec51d9b62cf3.shtml](https://www.nhc.gov.cn/yzygj/c100068/202009/10ffeb2e074b4d6b9567ec51d9b62cf3.shtml) |
| 59 | China | 2020 | MOST of the PRC | 《Notice on the Approval of the Construction of the National Key Laboratory for Difficult, Critical, and Rare Diseases》 | Diagnosis and Treatment | <https://www.gov.cn/zhengce/zhengceku/2020-10/26/content_5554310.htm> |
| 60 | China | 2021 | NHSA | 《National Basic Medical Insurance, Work Injury Insurance, and Maternity Insurance Drug Directory (2021 Edition)》 | medical insurance | [https://file.m12333.cn/upfile/download/20ef3f17-aa0e-3513-d3b8-b2da74e8723f.pdf](https://file.m12333.cn/upfile/download/20ef3f17-aa0e-3513-d3b8-b2da74e8723f.pdf) |
| 61 | China | 2022 | CDE | 《Notice on the Release of the Statistical Guidance Principles for Clinical Research on Rare Disease Drugs (Trial Implementation)》 | R&D & Registration | <https://www.cde.org.cn/main/news/viewInfoCommon/058e0d665b785e79b7f4f24dc1dc970c> |
| 62 | China | 2022 | NMPA | 《Announcement on the Release of the Third Batch of Anticancer Drugs and Rare Disease Drugs Eligible for Value-Added Tax Policies》 | R&D & Registration | <https://www.gov.cn/zhengce/zhengceku/2022-11/22/content_5728197.htm> |
| 63 | China | 2022 | NHC of the PRC | 《Notice on Issuing the Establishment Standards for National Rare Disease Medical Centers》 | Diagnosis and Treatment | [https://www.gov.cn/zhengce/zhengceku/2022-12/29/content_5734031.htm](https://www.gov.cn/zhengce/zhengceku/2022-12/29/content_5734031.htm) |
| 64 | China | 2023 | CDE | 《Guidelines for Accelerating the Review of Marketing Authorization Applications for Innovative Drugs by the Center for Drug Evaluation (Trial Implementation)》 | R&D & Registration | https://www.cde.org.cn/main/news/viewInfoCommon/ace377c025ad4f2bbf94790673b2646e |
| 65 | China | 2023 | NHC of the PRC | 《Second Batch of Rare Diseases List》 | Management | <https://www.gov.cn/zhengce/zhengceku/202309/content_6905273.htm> |
| 66 | China | 2023 | CDE | 《Guidance Principles for Natural History Studies in Rare Disease Drug Development》 | Diagnosis and Treatment | <https://www.cde.org.cn/main/news/viewInfoCommon/beef37b41b0a2d10b72ba1465a7a19e1> |
| 67 | China | 2023 | CDE | 《Technical Guidance Principles for the Design of Clinical Trials of Gene Therapy for Hemophilia》 | Diagnosis and Treatment | https://www.cde.org.cn/main/news/viewInfoCommon/a0470fe8e6a9c38fb71e0b125d5f0762 |
| 68 | China | 2023 | CDE | 《Notice on Soliciting Public Comments on the Draft Technical Guidance Principles for Clinical Trials of Rare Disease Gene Therapy Products》 | R&D & Registration | https://www.cde.org.cn/main/news/viewInfoCommon/588796dc6f1dc65265e31728f6c451e4 |
| 69 | China | 2023 | CDE | 《Notice on Soliciting Public Comments Regarding the "Guidance Principles for Non-Clinical Research of Enzyme Replacement Therapy Drugs for Rare Diseases (Draft for Comment)"》 | R&D & Registration | https://www.cde.org.cn/main/news/viewInfoCommon/c48f039d2d540308da2c5261785bafff |
| 70 | China | 2024 | CDE | 《Technical Guidance Principles for the Application of Decentralized Clinical Trials in the Clinical Development of Orphan Drugs》 | R&D & Registration | https://www.cde.org.cn/main/news/viewInfoCommon/e5b3409ea38fbc8254bb0635d004c73d |
| 71 | China | 2024 | CDE | 《Pilot Program for Patient-Centered Rare Disease Drug Development ("Care Initiative")》 | R&D & Registration | https://www.cde.org.cn/main/news/viewInfoCommon/244dc3661a418359aa12d7cba9bacf5d |
| 72 | China | 2024 | CDE | 《Technical Guidelines for Model-Informed Drug Development for Rare Diseases (Draft for Comment)》 | R&D & Registration | [https://www.cde.org.cn/main/news/viewInfoCommon/258189515b8d3df9964e275b26ba901b](https://www.cde.org.cn/main/news/viewInfoCommon/258189515b8d3df9964e275b26ba901b) |
| 73 | China | 2024 | CDE | 《Technical Guidelines for Clinical Pharmacology Studies of Drugs for Rare Diseases (Draft for Comment)》 | R&D & Registration | <https://www.ydcdei.org.cn/news/show/1044586254156705792> |
| 74 | China | 2025 | NHSA | 《Work Plan for Adjusting the 2025 National Basic Medical Insurance, Maternity Insurance, and Work Injury Insurance Drug Directory and the Commercial Health Insurance Innovative Drug Directory》 | medical insurance | https://www.nhsa.gov.cn/art/2025/8/12/art_109_17559.html |
| 75 | China | 2025 | NHC of the PRC | 《Notice of the General Office of the National Health Commission on Issuing the Diagnosis and Treatment Guidelines for 86 Rare Diseases Including Achondroplasia (2025 Edition)》 | Diagnosis and Treatment | <https://www.gov.cn/zhengce/zhengceku/202507/content_7031426.htm> |
| 76 | China | 2025 | CDE | 《Guidance Principles for Pharmaceutical Research on Chemical Drugs for Rare Diseases (Draft for Comment)》 | R&D & Registration | https://www.cde.org.cn/main/news/viewInfoCommon/f980065f05956831a2cfd5a5f0f65b2a |
| 77 | Brazil | 2001 | Brazilian Ministry of Health | 《Decree - Law No. 822》 | Management, Prevention | [https://bvsms.saude.gov.br/bvs/saudelegis/gm/2001/prt0822_06_06_2001.html](https://bvsms.saude.gov.br/bvs/saudelegis/gm/2001/prt0822_06_06_2001.html) |
| 78 | Brazil | 2014 | Brazilian Ministry of Health | 《National Policy for Comprehensive Care for People with Rare Diseases》 | Management, Diagnosis and Treatment | [https://bvsms.saude.gov.br/bvs/saudelegis/gm/2014/prt0199_30_01_2014.html](https://bvsms.saude.gov.br/bvs/saudelegis/gm/2014/prt0199_30_01_2014.html) |
| 79 | Brazil | 2014 | Brazilian Ministry of Health | 《Decree No. 981》 | Management, Diagnosis and Treatment | [https://bvsms.saude.gov.br/bvs/saudelegis/gm/2014/prt0981_21_05_2014.html](https://bvsms.saude.gov.br/bvs/saudelegis/gm/2014/prt0981_21_05_2014.html) |
| 80 | Brazil | 2015 | Brazilian Ministry of Health | 《Prioritization of Protocols and Therapeutic Guidelines for Comprehensive Care for People with Rare Diseases》 | Diagnosis and Treatment | [relatrio_pcdt_doenasraras_cp_final_142_2015.pdf](https://docs.bvsalud.org/biblioref/2017/11/874983/relatrio_pcdt_doenasraras_cp_final_142_2015.pdf) |
| 81 | Brazil | 2016 | Federal Government of Brazil | 《House Bill No. 56》 | Diagnosis and Treatment, medical insurance | [https://www25.senado.leg.br/web/atividade/materias/-/materia/126956](https://www25.senado.leg.br/web/atividade/materias/-/materia/126956) |
| 82 | Brazil | 2017 | ANVISA | 《RESOLUTION - RDC No. 205》 | Management, R&D & Registration | [https://www.gdtbt.org.cn/html/note-178402.html](https://www.gdtbt.org.cn/html/note-178402.html) |
| 83 | Brazil | 2017 | ANVISA | 《Draft Resolution No. 355》 | Management, R&D & Registration | [https://www.gdtbt.org.cn/html/note-157304.html](https://www.gdtbt.org.cn/html/note-157304.html) |
| 84 | Brazil | 2017 | Brazilian Ministry of Health | 《RESOLUTION No. 563》 | Management, Diagnosis and Treatment | [https://www.gov.br/conselho-nacional-de-saude/pt-br/acesso-a-informacao/atos-normativos/resolucoes/2017/resolucao-no-563.pdf/view](https://www.gov.br/conselho-nacional-de-saude/pt-br/acesso-a-informacao/atos-normativos/resolucoes/2017/resolucao-no-563.pdf/view) |
| 85 | Brazil | 2020 | Brazilian Ministry of Health | 《Decree No. 1,111》 | Diagnosis and Treatment | [https://bvsms.saude.gov.br/bvs/saudelegis/Saes/2020/prt1111_11_12_2020.html](https://bvsms.saude.gov.br/bvs/saudelegis/Saes/2020/prt1111_11_12_2020.html) |
| 86 | Brazil | 2021 | Federal Government of Brazil | 《LAW No. 14,154》 | Management, Diagnosis and Treatment | [https://www.planalto.gov.br/ccivil_03/_ato2019-2022/2021/lei/L14154.htm](https://www.planalto.gov.br/ccivil_03/_ato2019-2022/2021/lei/L14154.htm) |
| 87 | Brazil | 2022 | ANVISA | 《Draft Resolution No. 1115》 | R&D & Registration | [https://www.gdtbt.org.cn/html/note-339345.html#wypy](https://www.gdtbt.org.cn/html/note-339345.html#wypy) |
| 88 | Brazil | 2023 | Federal Government of Brazil | 《LAW No. 14,593》 | Management, Other Rare Disease-Related Policies | [https://www.planalto.gov.br/ccivil_03/_Ato2023-2026/2023/Lei/L14593.htm#art2](https://www.planalto.gov.br/ccivil_03/_Ato2023-2026/2023/Lei/L14593.htm) |
| 89 | Brazil | 2024 | Brazilian Ministry of Health | 《GM/MS ADMINISTRATIVE RULE No. 3,132》 | Management, Diagnosis and Treatment | [https://bvsms.saude.gov.br/bvs/saudelegis/gm/2024/prt3132_20_02_2024.html](https://bvsms.saude.gov.br/bvs/saudelegis/gm/2024/prt3132_20_02_2024.html) |
| 90 | Brazil | 2024 | Brazilian Ministry of Health | 《GM/MS Ordinance No. 6,324》 | Management, medical insurance | <https://www.gov.br/saude/pt-br/composicao/sectics/rename> |
| 91 | Brazil | 2025 | Brazilian Ministry of Health | 《Clinical Protocols and Therapeutic Guidelines 》 | Diagnosis and Treatment | <https://www.gov.br/saude/pt-br/assuntos/pcdt> |
| 92 | Ethiopia | 2014 | EFDA | 《LIST OF ORPHAN MEDICINES FOR RARE AND DIFFICULT TO TREAT DISEASES IN ETHIOPIA》 | Management | [https://www.efda.gov.et/wp-content/uploads/2019/03/LIST-OF-ORPHAN-MEDICINES-FOR-ETHIOPIA-2014.pdf?utm_source=chatgpt.com](https://www.efda.gov.et/wp-content/uploads/2019/03/LIST-OF-ORPHAN-MEDICINES-FOR-ETHIOPIA-2014.pdf?utm_source=chatgpt.com) |
| 93 | Ethiopia | 2023 | EFDA | 《Guideline on Medical Products Special import permit》 | R&D & Registration | [https://www.efda.gov.et/wp-content/uploads/2025/02/2023.12.21-Guideline-on-Medical-Products-Special-Import-Permit.pdf?utm_source=chatgpt.com](https://www.efda.gov.et/wp-content/uploads/2025/02/2023.12.21-Guideline-on-Medical-Products-Special-Import-Permit.pdf?utm_source=chatgpt.com) |
| 94 | Ethiopia | 2024 | MOH | 《National Preconception Care Guideline》 | Prevention | [https://www.moh.gov.et/sites/default/files/2024-07/National%20Preconception%20care%20guideline_2024.pdf](https://www.moh.gov.et/sites/default/files/2024-07/National Preconception care guideline_2024.pdf" \o "https://www.moh.gov.et/sites/default/files/2024-07/National%20Preconception%20care%20guideline_2024.pdf) |
| 95 | Uganda | 2023 | MOH | 《Uganda Clinical Guidelines 2023: National Guidelines for Management of Common Health Conditions》 | Diagnosis and Treatment | <https://library.health.go.ug/uganda-clinical-guidelines-2023> |
| 96 | Uganda | 2024 | MOH | 《The National Essential Health Care Package for Uganda》 | Prevention | <https://cphl.go.ug/ministry-health-launches-lifesaving-sickle-cell-care-program-kayunga-regional-referral-hospital?utm_source=chatgpt.com> |
| 97 | Uganda | 2024 | NDPA | 《Conduct Clinical Trials Regulations》 | R&D & Registration | https://www.nda.or.ug/wp-content/uploads/2024/10/Conduct-of-Clinical-Trials-Regulation-2024-compressed.pdf |

**Abbreviations:** FDA, Food and Drug Administration; HHS, U.S. Department of Health and Human Services; EP, The European Parliament; EUC, The Council Of The European Union; EC, European Commission; EMA, EUROPEAN MEDICINES AGENCY; DG SANTE, Directorate-General for Health and Food Safety; NHSE, NHS England; DHSC, Department of Health and Social Care; NICE, National Institute for Health and Care Excellence; NHC of the PRC, National Health Commission of the People's Republic of China; NMPA, National Medical Products Administration; NPCSC, The National People's Congress Standing Committee; MOST of the PRC, Ministry of Science and Technology of the People's Republic of China; NHSA, National Healthcare Security Administration; CDE, Center for Drug Evaluation; ANVISA, Brazil's National Health Regulatory Agency; EFDA, Ethiopian Food and Drug Authority; MOH, Ministry of Health-Ethiopia; NDPA, The National Drug Policy and Authority.
